# Synthesis, Structural Characterization and Reactivity of Macrocyclic Cyclo[2]malonates

**DOI:** 10.1021/acsomega.6c03626

**Published:** 2026-06-08

**Authors:** Samuele Ruffoli, Andrea Vitale, Kevin D’Addazio, Alessandro Pispero, Daniele Sartore, Demetra Giuri, Claudia Tomasini, Simone D’Agostino

**Affiliations:** Dipartimento di Chimica Giacomo CiamicianCiamician and INSTM Research Unit, 9296 Università di Bologna, Via Piero Gobetti, 85, 40129 Bologna, Italy

## Abstract

We report the controlled synthesis of a new class of oxygen-bridged cyclo[2]­malonates via condensation of malonyl dichloride with substituted benzylic α,ω-diols. Optimization of reaction conditions enables selective access to dimeric macrocycles, overcoming the intrinsic tendency toward higher oligomer formation. Single-crystal X-ray diffraction reveals well-defined conformations, tunable cavity sizes, and substituent-dependent stereochemical preferences. The macrocycles assemble into interlocked columnar architectures driven by shape complementarity and stabilized by C–H···O hydrogen bonding, with additional halogen bonding in iodinated derivatives. While α-proton acidity is retained across the series, vanadyl (VO^2+^) binding is strongly governed by cavity size and conformational preorganization, suggesting a structure–function relationship. These findings introduce cyclo[2]­malonates as an accessible and versatile macrocyclic platform, combining structural tunability with predictable supramolecular behavior, and highlight their potential in molecular recognition and functional materials design.

## Graphical abstract

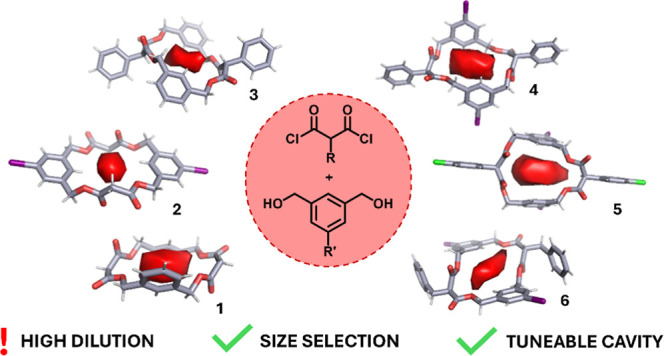

## 1 Introduction

Macrocycles constitute an important class of compounds in organic chemistry, with applications spanning medicinal chemistry,^1^ supramolecular chemistry,^2,3^ and catalysis.^4^ Their structural diversity and ability to adopt well-defined three-dimensional conformations make them valuable scaffolds for molecular recognition, host–guest chemistry, and drug design.^5–7^

Despite their broad utility, the synthesis of medium and large-sized macrocycles (≥8 atoms) remains a longstanding challenge due to the entropic and enthalpic barriers inherent to ring closure.^8–13^ To overcome these challenges, a wide range of macrocyclization strategies has been developed. Classical approaches include macrolactonization and macrolactamization in the synthesis of macrolide antibiotics and cyclic peptides,^14–23^ as well as ring-closing metathesis,^24^ which has expanded access to diverse olefin-containing macrocycles and cyclophanes. In recent years, click chemistry has emerged as a particularly powerful tool for constructing macrocyclic architectures.^25–27^ Notably, the synthesis of 24-membered triazine-based macrocycles has been achieved through click-based methodologies, providing access to rigid and well-defined structures.^28^

Amide macrocycles are versatile supramolecular hosts due to their directional hydrogen bonding, structural rigidity, and modularity.^29^ Cyclic peptides and aromatic derivatives dominate this class, offering tunable cavities and binding properties. These systems enable advanced functions, including molecular recognition, catalysis, ion transport, and the formation of higher-order architectures.^30–33^

Other examples of macrocycles include systems lacking heteroatoms, such as those reported by Alberts and Cram,^34^ who described the synthesis of macrocyclic acetylacetone ligands and systematically compared their metal–cation binding properties with those of acyclic analogues. Incorporation of multiple acetylacetone units into a macrocyclic framework result in a pronounced enhancement of complex stability, attributable to ligand preorganization.

The synthesis of purely carbocyclic macrocycles is intrinsically challenging, as macrocyclization reactions often proceed in low yields and suffer from limited reproducibility. The incorporation of oxygen atoms into the macrocyclic backbone can significantly facilitate cyclization, since carbon–oxygen bond formation is generally more favorable from both kinetic and thermodynamic perspectives and can be achieved through a broader range of well-established synthetic transformations.

More recently, Diederich and co-workers reported an efficient one-step synthetic approach to macrocyclic cyclo­[n]­malonates based on the condensation of malonyl dichloride with a series of α,ω-diols. In these systems, both the nature and the length of the spacer were shown to play a crucial role in determining the selectivity of the macrocyclization process, enabling the preferential formation of mono-, di-, or trimeric macrocycles. In addition to their synthetic accessibility, bismalonate macrocycles are characterized by a marked tendency to crystallize and by the adoption of well-defined conformations in the solid state, features that make them particularly attractive for structural and supramolecular investigations.^35–38^

Single-crystal X-ray analyses reveal columnar stacking stabilized by weak C–H···O interactions, with structural features enforced by the macrocyclic framework.

## 2 Results and Discussion

Among the various possible macrocyclic architectures,^35,36^ this study focuses on the design, synthesis, and structural characterization of oxygen-bridged macrocycles, together with the optimization of reaction conditions to achieve selective control over ring size. In particular, the macrocyclization of malonyl dichloride with 1,3-bis­(hydroxymethyl)­benzene was optimized to favor formation of the dimeric cyclo[2]­malonate. The synthesized compounds display a pronounced tendency to crystallize. Single-crystal X-ray diffraction (SCXRD) measurements and subsequent structural analyses reveal that bismalonate macrocycles adopt well-defined conformations in the solid state.

### 2.1 Synthesis of the Cyclo[2]­malonates

The synthesis of cyclo[2]­malonates **1**-**6** is achieved by a one-step condensation of malonyl dichloride with benzylic α,ω-diols. Using this strategy, six cyclo[2]­malonates sharing a common structural framework were prepared (Scheme 1), and their solid-state organization was investigated. The overall aim is 2-fold: to access structurally robust macrocycles suitable for molecular recognition and to introduce synthetically versatile substituents enabling further functionalization and incorporation into more complex molecular architectures.

**Scheme 1:**
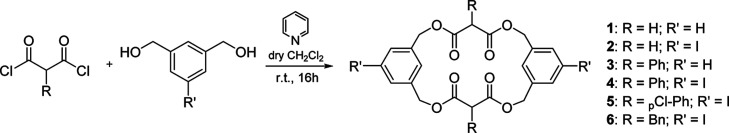
General Method of the Synthesis of cyclo[2]­malonate

The six derivatives were synthesized through systematic variations of substituents *R* and *R*′. All starting materials employed in the macrocyclization were either commercially available or readily prepared in a limited number of steps^39,40^ (see Supporting Information for details). A major synthetic challenge associated with this system is the control of macrocycle size, as the condensation reaction can lead to the concurrent formation of dimeric, trimeric, and tetrameric species. Consequently, reaction conditions must be carefully optimized to favor selective formation of the desired ring size.

Although condensation of malonyl dichloride with 1,3-bis­(hydroxymethyl)­benzene proceeds smoothly under basic conditions, the reaction is inherently equilibrated, resulting in the formation of higher macrocyclic homologues, particularly cyclo[3]­malonates.

The general procedure involves the dropwise addition of a solution of malonyl dichloride in anhydrous dichloromethane to a stirred solution of 1,3-bis­(hydroxymethyl)­benzene and pyridine in anhydrous dichloromethane at room temperature. Systematic optimization of reaction parameters, including reagent concentration, temperature, and base equivalents, minimized competing pathways and enabled the reproducible formation of the dimeric cyclo[2]­malonate in satisfactory yields.

Table 1 summarizes the effects of addition time and reagent concentration, which proved to be the most critical parameters for maximizing the yield of the cyclo[2]­malonates. The study was conducted using cyclo[2]­malonate **1** as a model substrate, and the optimized conditions were successfully applied to the synthesis of compounds **2**–**6**. Optimal results were obtained under high-dilution conditions combined with a prolonged dropwise addition of the malonyl dichloride solution.

**Table 1:** Optimization of the Dripping Time and Reagents Concentrations for the Formation of cyclo[2]­malonate **1**

entry	conc. *m*-C_6_H_4_(CH_2_OH)_2_ (mM)	conc. CH_2_(COCl)_2_ (mM)	conc. pyridine (mM)	dripping time (min)	yield (%)
1	100	100	200	120	7
2	50	50	100	40	15
3	50	50	100	120	20
4	25	25	50	180	15
5	12.5	12.5	25	150	18
6	12.5	12.5	25	240	22
7	12.5	13.8	29	240	25

On the basis of these optimized conditions, a small library of structurally related macrocycles bearing diverse aromatic and α-methylene substituents was subsequently prepared (Table 2), under the optimized conditions reported in Table 1, entry 7. Unfortunately, the yields of the purified products never exceed 35%. In addition, for compounds **5** and **6** the yields were largely unsatisfactory, probably due to bulkier substituents of the malonyl moieties that largely reduce their reactivity. Finally, cyclo[2]­malonates **3**–**6** bear substituents on the malonyl moieties; therefore, dimer formation can give rise to diastereomeric mixtures, which can be readily identified by ^1^H NMR analysis (see Supporting Information).

**Table 2:** Final Yield for the Synthesis of cyclo[2]­malonate **1**–**6** Under Optimized Conditions (Table 1, Entry 7) and After Purification

product	*R*	*R*′	yield (%)^a^
1	H	H	25
2	H	I	20
3	Ph	H	35
4	Ph	I	35
5	_p_Cl-Ph	I	15
6	Bn	I	19

a The yield of cycles **1**–**6** were obtained after purification by flash chromatography.

An additional factor affecting yield optimization is the formation of byproducts. HPLC–MS analysis of the crude reaction mixtures consistently revealed the presence of macrocyclic cyclo[3]­malonates and cyclo[4]­malonates as minor byproducts, although in variable amounts. Table 3 reports the relative ratios of the three species as determined by HPLC–MS analysis (see the Supporting Information for complete analyses). The ratios are normalized to a total of 100% and are not intended to reflect the final isolated yields of cyclo[2]­malonates **1**–**6**. Two factors contribute to this discrepancy: (i) the starting materials, which are small molecules, cannot be reliably detected by HPLC–MS under these conditions; and (ii) purification by silica gel chromatography is challenging, requiring prolonged purification times that inevitably reduce the isolated yields.

**Table 3:** Relative Ratios of Cyclo[2]-, Cyclo[3]-, and Cyclo[4]­malonates in Crude Reaction Mixtures for the Formation of Macrocycles **1**–**6**, Determined by HPLC–MS

product	*R*	*R*′	cyclo[2]malonate (%)^a^	cyclo[3]malonate (%)^a^	cyclo[4]malonate (%)^a^
1	H	H	54	26	20
2	H	I	67	25	8
3	Ph	H	65	25	10
4	Ph	I	71	29	n.d^b^
5	_p_Cl-Ph	I	72	28	n.d^b^
6	Bn	I	70	22	8

a The percentages of cyclo­[*n*]­malonates were obtained by with HPLC–MS analysis of the crudes.

b No peak with a mass analysis in agreement with the cyclo[4]­malonate structure was detected.

Overall, cyclo[2]­malonates are consistently the predominant products, with relative ratios ranging from 53.8% to 72.0%. With the exception of unsubstituted macrocycle **1**, the relative abundances of the other cyclo[2]­malonates show only minor variations, indicating that substitution has a limited influence on the formation of the dimeric species. This behavior suggests that the formation of cyclo[2]­malonates is primarily governed by favorable entropic and kinetic factors, which promote intramolecular ring closure over further oligomerization.

Similarly, the relative proportions of cyclo[3]­malonates remain largely unaffected by the nature of the substituents, consistent with a pathway that is less sensitive to steric and electronic effects. In contrast, the formation of cyclo[4]­malonates is strongly dependent on substitution, decreasing markedly from 20.1% in the unsubstituted system **1** to undetectable levels in more substituted derivatives. This trend can be rationalized by increased steric hindrance and reduced conformational flexibility in substituted systems, which disfavor the larger ring closure required for tetramer formation.

In addition, macrocycle **7** was prepared via Sonogashira cross-coupling,^41^ in which the aryl iodide substituents of **4** were replaced by ethynyl groups in excellent yield. This transformation demonstrates the robustness of the cyclo[2]­malonate scaffold toward postsynthetic modification without compromising molecular integrity (Scheme 2). Macrocycle **7** can be synthesized either through isolation of the intermediate trimethylsilyl-protected alkyne (see Supporting Information for details), followed by deprotection with tetrabutylammonium fluoride, or via a one-pot protocol combining both steps.^42^ The one-pot approach afforded macrocycle **7** in good yield after purification and was therefore adopted as the preferred method.

**Scheme 2:**
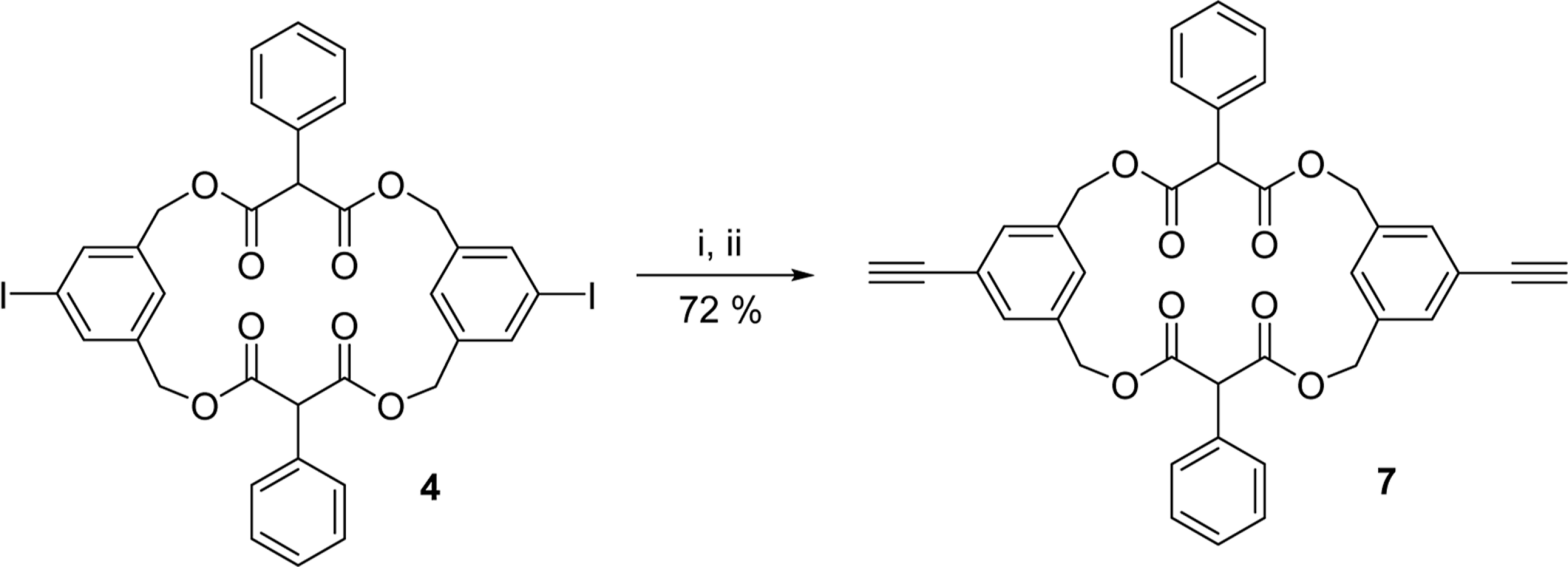
Reagents and Conditions (i) CH≡C–Si­(Me)_3_, HC­(PPh_3_)_2_PdCl_2_, Cul, TEA, THF, r.t., 16 h; (ii) TBAF, THF, r.t., 1.5 h

### 2.2 Structural Studies of the Cyclo[2]­malonates via Single-Crystal X-ray Diffraction

A notable feature of this class of macrocycles is their pronounced propensity to crystallize, readily affording well-defined crystals from a variety of solvents (see the Experimental Section for details). Therefore, to gain insight into their structural features, single-crystal X-ray diffraction (SCXRD) studies were undertaken. This technique enabled the analysis of their preferred conformations in the solid state, which are dictated by the type of substituents bound to the α position of the malonate residue and to the aromatic ring of the benzylic diol moiety (see Schemes 1 and 2). Structural analysis confirms the molecular structures of all synthesized macrocycles (see Figure 1) and reveals that they crystallize in either the monoclinic or triclinic crystal systems (see Table S1 for details).

**Figure 1:**
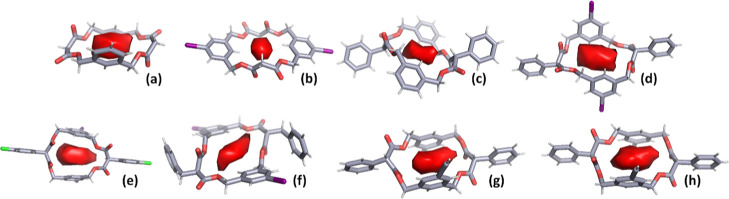
Molecular structures, as determined by SCXRD, and representation of inner cavity voids (red volumes) detected in crystalline compounds: (a) **1**, (b) **2**, (c) **3**, (d) **4**, (e) **5**, (f) **6**, (g) **7-cis**, and (h) **7-trans**. The cis and trans isomers are simultaneously present in the crystalline lattice of compound **7**, therefore, the voids for **7** *-*cis and **7** *-*trans were calculated from model crystal packings of the individual isomers.

It is worth noting that macrocyclic compounds bearing α-substituted malonate residues (**3**–**7**) may exhibit stereochemical isomerism arising from *cis/trans* orientations of the α substituent with respect to the macrocyclic framework, leading to two diastereoisomers. In contrast, compounds **1** and **2** do not exhibit such isomerism because they lack substitutions at the α position of the malonate moiety.

From crystallization experiments, compounds **3**, **4**, and **6** were isolated as *trans* isomers configuration, whereas compound **5** was obtained as the *cis* isomer. Interestingly, compound **7** crystallizes as a solid solution of diastereoisomers,^43–45^ i.e., forming a unique crystalline phase in which both the *cis* and *trans* conformations are simultaneously present (Figure S1) and evenly distributed, as confirmed by NMR spectroscopy measurements (Figure S2).

Beyond establishing the presence of diastereomeric forms, the type and nature of the substituents also influence the size of the inner cavity of each macrocycle (see Table 4 and Figure 1, where the red volumes represent the inner cavity voids).

**Table 4:** Estimated Central Cavity Volumes (Å^3^) and Main Intermolecular Interactions Detected in Crystalline Macrocycles **1**–**7**. Data for the *cis* and *trans* Isomers of Compound **7** are Reported Separately

	1	2	3	4	5	6	7-cis	7-trans
cavity size (Å^3^)	15.9	7.2	5.7	15.4	24.2	9.2	15.2	15.9
C_Ar_–H···O_CO_ (Å)	3.461(3)	-	3.481(4)	-	3.43(1)	3.441(4)	3.37(1)	3.48(1)
C_Me_–H···O_CO_ (Å)	3.353(4)	3.404(6)	3.353(4)	3.50(1)	3.38(1)	3.527(5)	3.33(2)	3.61(1)
C_Ar_–I···O_CO_ (Å)	-	3.459(4)	-	3.21(1)	-	-	-	-
C_Ar_–I···I–C_Ar_ (Å)	-	-	-	-	3.9891(8)	-	-	-

Structural analysis also reveals that, in each case, the macrocycles stack one into another by shape complementarity,^46^ i.e., interlocking themselves in a columnar fashion and forming tubular structures with almost occluded inner channels (see Figure S3 for the packing diagrams).

For all compounds, the crystal packings are primarily stabilized by dispersion forces and several weak intermolecular C–H···O hydrogen bonds^47^ between the C=O groups and the methylene groups of the malonates (C_Ar_–H···O_CO_), or between the malonate C=O groups and the benzylic diol aromatic moiety (C_Me_–H···O_CO_) and occur both within the stacks or between adjacent molecules (see Figure 2 and 3 and Table 4 for distances).

**Figure 2:**
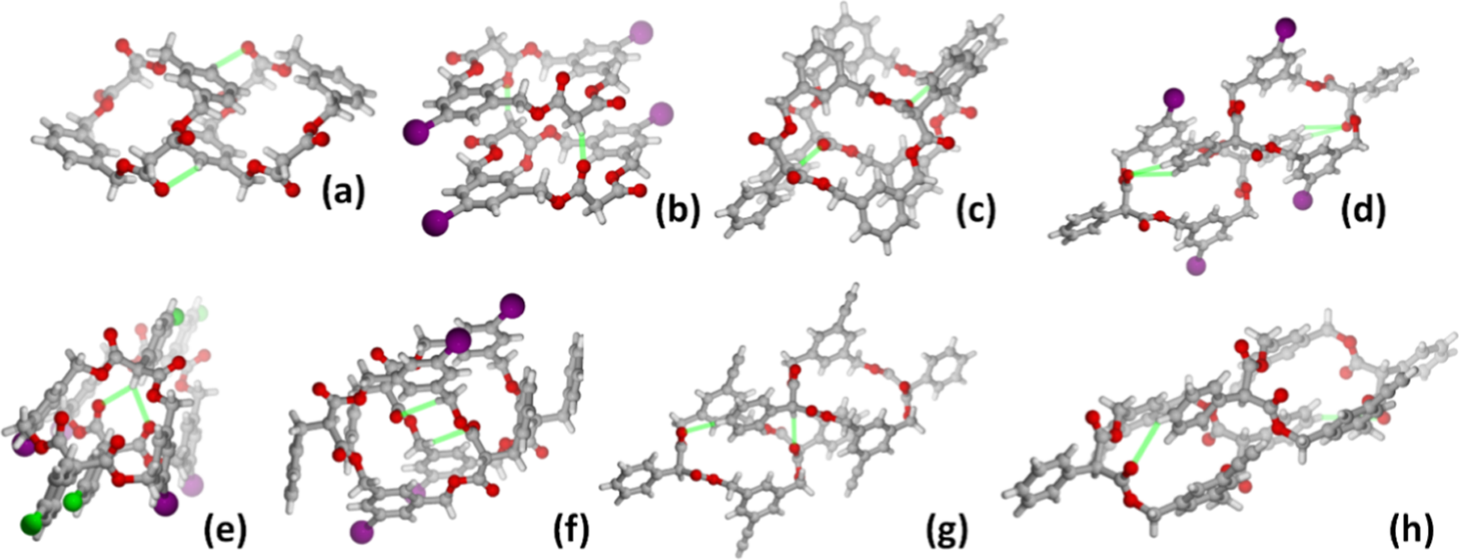
Weak C–H···O hydrogen bonds, depicted in green, detected within the columnar stacks of crystalline: (a) **1**, (b) **2**, (c) **3**, (d) **4**, (e) **5**, (f) **6**, (g) **7-** *cis*, and (h) **7-** *trans*.

**Figure 3:**
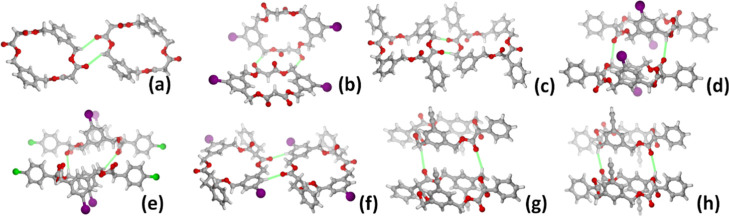
Weak C–H···O hydrogen bonds, depicted in green, detected between adjacent molecules in crystalline: (a) **1**, (b) **2**, (c) **3**, (d) **4**, (e) **5**, (f) **6**, (g) **7-cis**, and (h) **7-** *trans*.

As shown in Figure 4, in compounds **2**, **4**, and **5**, bearing iodo substituents, weak halogen-bonding interactions^48^ between adjacent columns are observed and further contribute to the stabilization of the overall crystal packing (see Table 4 for distances). In crystalline **2** and **4**, the halogen bonds occur between the iodine atom bound to the benzylic diol aromatic moiety and an oxygen atom of the malonate carbonyl group (C_Ar_–I···O_CO_), whereas in **5** and **6** they are expected to occur between iodine atoms bound to the benzylic diol aromatic moieties (C_Ar_–I···I–C_Ar_); however, in **6** the I···I distance accounts for 4.4456(5) Å,, which is significantly longer than the sum of the van der Waals radii and therefore cannot be regarded as a genuine halogen-bonding interaction.

**Figure 4:**
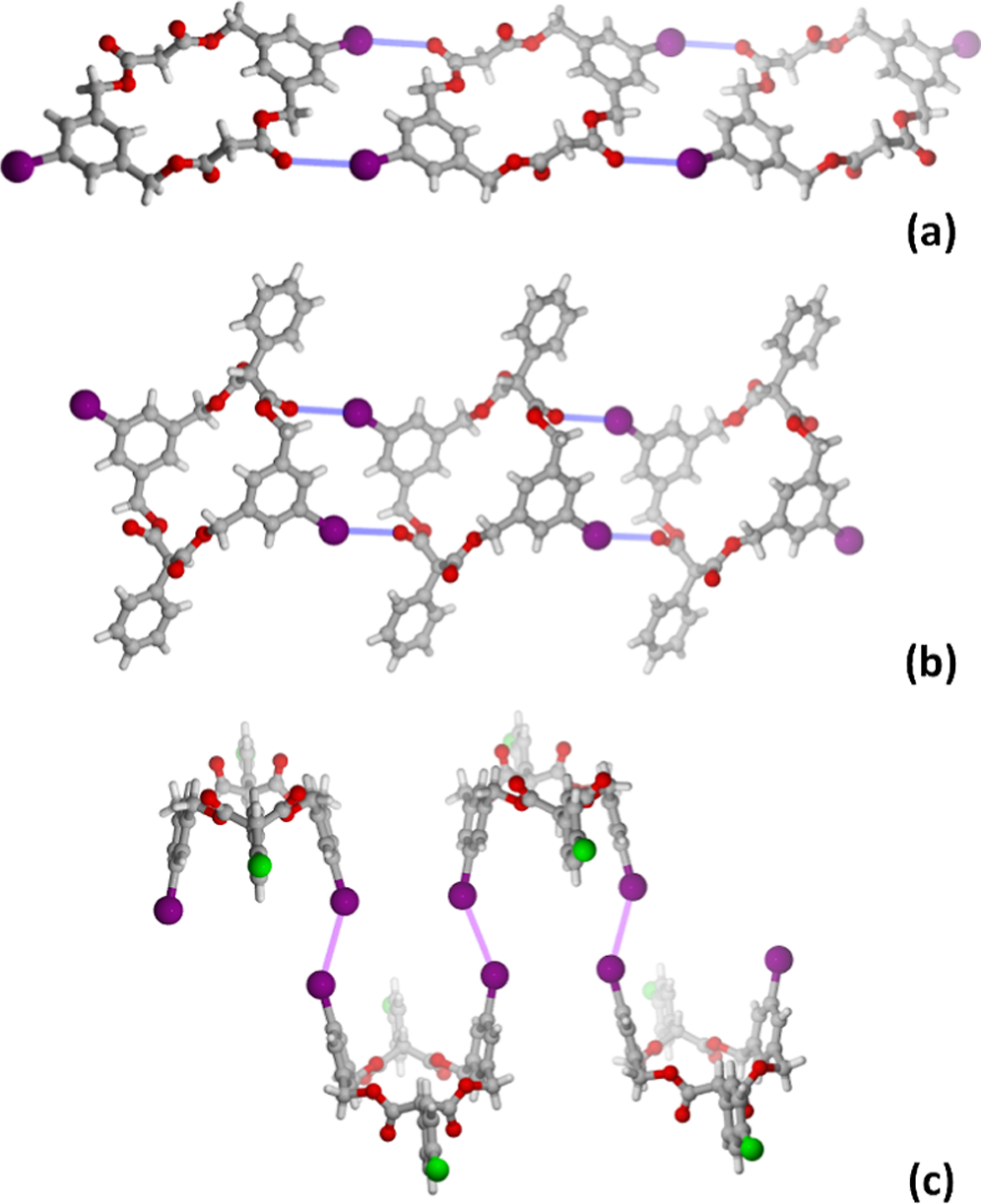
Representation of the weak halogen bonding interactions detected within crystalline: (a) **2**, (b) **4**, and (c) **5**. C_Ar_–I···O_CO_ and C_Ar_–I···I–C_Ar_ depicted in blue and violet, respectively.

Overall, the crystallization behavior for such compounds is consistent with previous observations in macrocyclic systems, where conformational strain and packing effects determine the final solid-state structure.^2,26,27^

Namely, the preference of compounds **3**, **4**, and **6** to crystallize as the *trans* isomer might be explained by concomitant conformational and packing effects. In such systems, the *trans* arrangement of the α-substituents is expected to minimize steric repulsion between substituents and the macrocyclic backbone, thus reducing overall conformational strain,^26,49^ and also a more efficient crystal packing by facilitating intermolecular interactions, providing, in this way, an additional driving force for the preferential crystallization of the *trans* isomer. On the other hand, the isolation of compound **5** as the *cis* isomer suggests that specific substituents, i.e., concomitant presence of chlorine and iodine atoms, may locally stabilize this configuration. Similarly, the formation of a *cis/trans* solid solution in compound **7** indicates that the two diastereoisomers are likely close in energy, allowing both to be accommodated within the same crystal lattice without significant disruption of the packing arrangement.

To test this hypothesis, the magnitude of intermolecular interactions in the two isomers was evaluated through Intermolecular Interaction Energies (IIEs) analyses;^50^ implemented in the CrystalExplorer package^51^ (see Experimental Section for details). In both cases, the IIE calculations (Figure 5 and Table S2) indicate that the strongest interactions occur within the columnar arrangements shown in Figure 3g,h, with total energies of −107.8 and −103.1 kJ·mol^–1^ for **7**-*cis* and **7**-*trans*, respectively. As expected, the IIEs are dominated by the dispersion component, with only a minor contribution from the electrostatic term; in both cases, the overall magnitudes remain comparable within columns and between adjacent molecules (see Table 4). These results further support the conclusion that, in these systems, crystal packing is primarily governed by shape complementarity.^46^

**Figure 5:**
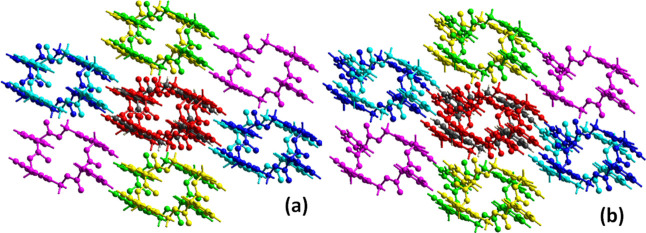
Pairs of interacting molecules that contribute most to the total interaction energy in crystalline: (a) **7-** *cis* and (b) **7-** *trans*.

### 2.3 Reactivity of the Cyclo[2]­malonates

To evaluate the ability of these macrocycles to host guest molecules within their cavities, we first investigated whether the cavity sizes determined by single-crystal X-ray diffraction (SCXRD) influence their binding behavior.

The α-proton acidity of the macrocycles was probed by treatment with potassium *tert*-butoxide under an inert atmosphere, followed by quenching with 35% DCl in D_2_O. When the α-position is sufficiently acidic, partial deprotonation–deuteration occurs, resulting in attenuation of the corresponding α-proton signal, as deuterium is not detected in ^1^H NMR spectra (Figures S4–S10).

Comparison of α-proton integrals with unaffected resonances indicates that, after deprotonation and deuteration, the α-hydrogen content decreases to approximately 40–50% of the initial value for all macrocycles examined. These results suggest that neither substitution on the macrocyclic framework nor cavity size significantly affects α-position reactivity under the applied conditions.

Macrocycles **1** and **2** each contain two chemically and magnetically equivalent α-hydrogens that do not couple with each other. Partial replacement of one α-hydrogen by deuterium leads to broadening of the remaining proton signal due to H–D coupling (Figures S4 and S5). In macrocycle **6** (Figure S9), the α-hydrogen is coupled to an adjacent methylene group; upon deuteration, this results in a more complex splitting pattern compared to the original doublet of doublets.

Reactivity toward VO_2_^+^ complex formation was further examined for macrocycles **1**–**7**. After treatment with potassium *tert*-butoxide in the selected solvent, an excess of VO­(NO_3_)_2_ was added under an argon atmosphere without prior quenching.^52^ After several trials, we selected dry 1,4-dioxane for products **1**–**6** and dry methanol for **7**. After 24–96 h, some reaction mixtures exhibited a color change from brown to olive green.

This color change suggested the possible formation of a vanadyl complex; therefore, the reaction mixture was analyzed by MALDI-TOF mass spectrometry as a preliminary screening method. Analysis of the crude reaction mixtures provided evidence of complex formation only for macrocycles **1, 3, and 7**, as indicated by the detection of peaks attributable to adducts between the macrocycles and the **VO**_**2**_^**+**^ cation. These results should be regarded as preliminary observations, intended to highlight the potential applications of this class of molecules. The outcomes of the MALDI-TOF experiments are summarized in Table 5. Unfortunately, attempts to obtain single crystals suitable for structural characterization have so far been unsuccessful, indicating that further optimization of the experimental conditions is required.

**Table 5:** Qualitative Results for the VO_2_^+^ Complex Formation from MALDI-TOF Analysis

product	*R*	*R*_1_	cavity size (Å^3^)	Mc mass	VO-Mc mass	selected MALDI-TOF peaks (*m/z*)	
**1**	H	H	15.9	412.12	477.04	451.08 (**1** + K^+^)	477.03 (**1** + VO^2+^)
**2**	H	I	7.2	663.91	728.83	702.86 (**2** + K^+^)	X
**3**	Ph	H	5.7	564.18	629.10	603.15 (**3** + K^+^)	X
**4**	Ph	I	15.4	815.97	880.89	854.95 (**4** + K^+^)	880.89 (**4**+VO^2+^)
**5**	4-Cl-Ph	I	24.2	883.89	948.82	922.87 (**5** + K^+^)	X
**6**	Bn	I	9.2	844.00	908.93	883.00 (**6** + K^+^)	X
**7**	Ph	ethyne	15.2	612.18	677.10	651.20 (**7** + K^+^)	709.00^a^ (**7**+VO^2+^+MeOH)
			15.9				

a This experiment was performed in dry methanol.

These preliminary results may be rationalized taking into consideration the ionic volume of the VO^2+^ ion, that was estimated from the effective ionic radii of its constituent ions reported by Shannon,^53,54^ and adopting the common assumption of additivity of ionic sizes. Therefore, using values of 0.53 Å and 1.40 Å for the radii of V^4+^ and O^2–^, respectively, the resulting volume of the VO^2+^ ion accounts for ca. 12.5 Å^3^. This simplified approach provides a reasonable first-order estimate; however, it neglects the intrinsic anisotropy associated with the V=O bond.

Macrocycles **2**, **3**, and **6** are unable to accommodate the VO^2+^ ion because their internal cavities are too small. In contrast, macrocycle **5** possesses a significantly larger cavity, which prevents the establishment of the weak interactions necessary to stabilize complex formation. Although the cavity sizes arise from the preferred conformations of the macrocycles, which are not rigidly constrained and could, in principle, undergo structural rearrangement, the energetic cost associated with altering these conformations remains too high for such changes to occur in solution without an external stimulus to promote complexation.

## 3 Conclusions

We report the rational design and controlled synthesis of a new class of oxygen-bridged cyclo[2]­malonates that provide access to structurally defined yet conformationally adaptable macrocyclic scaffolds. By systematically optimizing key reaction parameters, including reagent concentration and addition rate, we achieved selective formation of dimeric macrocycles from malonyl dichloride and substituted benzylic α,ω-diols, overcoming the inherent tendency of these systems to form higher oligomers. This study establishes a reliable synthetic strategy for accessing this underexplored family of macrocycles and demonstrates their tolerance to postfunctionalization through Sonogashira cross-coupling.

A distinctive feature of these cyclo[2]­malonates is their pronounced crystallinity, which enabled comprehensive structural elucidation by single-crystal X-ray diffraction across the entire series. The analysis reveals that subtle structural modifications translate into significant variations in cavity size, conformational preferences, and supramolecular organization. In particular, α-substitution induces diastereomerism and governs conformational selection, with trans isomers generally favored due to reduced steric strain and enhanced packing efficiency. Notably, specific substitution patterns can stabilize less common conformations or even generate solid solutions of diastereomers, highlighting the delicate interplay between molecular structure and crystal packing.

The macrocycles assemble into interlocked columnar architectures driven primarily by shape complementarity, representing a recurring and structurally robust motif. These assemblies are stabilized by a combination of weak but cooperative interactions, including C–H···O hydrogen bonding and, in iodinated derivatives, halogen bonding. This combination of predictable packing behavior and tunable cavity environments distinguishes cyclo[2]­malonates from more rigid or preorganized macrocyclic systems.

Importantly, we demonstrate that the functional properties of these macrocycles can be directly correlated with their structural features. Deuteration studies confirm that α-proton acidity is preserved across the series, indicating that electronic properties are largely insensitive to substitution and cavity size. In contrast, vanadyl (VO^2+^) binding appears to be strongly structure-dependent, suggesting a possible structure–function relationship. Under the present experimental conditions, evidence of complex formation was observed only for macrocycles possessing appropriately sized and conformationally preorganized cavities. In contrast, smaller cavities may sterically hinder guest inclusion, whereas larger cavities may not provide sufficiently strong stabilizing interactions. Although these findings should still be considered preliminary, they suggest that the conformational organization of the macrocycles—and, consequently, the resulting cavity geometry—plays a significant role in the observed host–guest recognition behavior. Further investigations are currently underway, including optimization of the complexation conditions and expansion of the analytical studies, in order to validate and further elucidate these preliminary observations.

Overall, this work introduces cyclo[2]­malonates as a versatile and previously underutilized platform for macrocycle design. Their combination of synthetic accessibility, structural tunability, and predictable supramolecular behavior opens new opportunities for their application in molecular recognition, adaptive host–guest systems, and the development of functional materials.

## 4 Experimental Section

### 4.1 General Remarks for the Synthetic Procedure

All reactions were carried out in dried glassware. The melting points of the compounds were determined in open capillaries and are uncorrected. All compounds were dried in vacuo and all the sample preparations were performed in a nitrogen atmosphere. High quality infrared spectra (64 scans) were obtained at 2 cm^–1^ resolution with an ATR-IR Agilent (Santa Clara, CA, USA) Cary 630 FTIR spectrometer. NMR spectra were recorded with Bruker NeoAvance 600 at 600 MHz (^1^H NMR) and at 125 MHz (^13^C NMR) and with a Varian (Palo Alto, CA, USA) Inova 400 spectrometer at 400 MHz (^1^H NMR). Chemical shifts are reported in δ values relative to the solvent peak. An Agilent (Santa Clara, CA, USA) 1260 Infinity II liquid chromatograph coupled to a Mass Spectrometer MSD/XT equipped with an electrospray ionization source and operating with a single quadrupole mass analyzer was used to check the purity of compounds. The HPLC was equipped with a Phenomenex Gemini C18 – 3 μ—110 Å column (40 °C) and H_2_O/CH_3_CN was used as solvent. The MS was used in positive ion mode, *m*/*z* = 50–2000, fragmentor 70 V. Milli-Q water (Millipore, resistivity = 18.2 mΩ cm) was used throughout. MALDI-TOF mass spectra were acquired on a Waters Synapt G2MALDI Q-TOF mass spectrometer. Samples were prepared by mixing the analyte with α-cyano-4-hydroxycinnamic acid (CHCA) matrix (typically in a 1:1 v/v ratio) in an appropriate solvent and spotted onto a stainless-steel target plate, followed by air drying. Spectra were recorded in the *m*/*z* range 100–2000 using a solid-state Nd/YAG laser (λ = 355 nm) operating at a repetition rate of 2.5 kHz. The products (5-iodo-1,3-phenylene)­dimethanol, 2-phenylmalonyl dichloride, 2-(4-chlorophenyl)­malonyl dichloride 2-benzylmalonyl dichloride were prepared according to known procedures described in the Supporting Information. Other chemicals and solvents were purchased from Sigma-Aldrich (St. Louis, MO, USA).

### 4.2 Synthesis of cyclo[2]­malonate **1**

In a three necks, round-bottom flask, dried under inert atmosphere (nitrogen or argon), 1,3-benzendimethanol (69.1 mg, 0.5 mmol) is added and dissolved in anhydrous CH_2_Cl_2_ (25 mL). Anhydrous pyridine (92 μL, 1.15 mmol) is added in the flask. The other anhydrous CH_2_Cl_2_ (15 mL) is added in the dropping funnel with malonyl dichloride (77.5 mg, 0.55 mmol). The solution is added dropwise within 4 h. After 16 h the reaction is washed three times with H_2_O. The organic phase is dried over Na_2_SO_4_ and evaporated. The crude reaction mixture is purified by flash column chromatography with cyclohexane-EtOAc 8:2 as mobile phase. The macrocyclic product is obtained as white solid with yield of 25%. Mp 158–162 °C; ^1^H NMR: (600 MHz, CDCl_3_): δ 7.33–7.25 (m, 8H, C-HAr), 5.13 (s, 8H, CH_2_O), 3.48 (s, 4H, CH_2_α); ^13^C NMR: (150 MHz, CDCl_3_): δ 166.19, 135.76, 128.86, 128.77, 128.45, 67.11, 42.01; IR-ATR: ν 2957, 2918, 2851, 1723, 1655, 1460, 1406, cm^–1^; HPLC-MS­(ESI): 8.21 min; mass calcd 412.12; found, 435.0 [M + Na]^+^, 451.0 [M + K]^+^.

### 4.3 Synthesis of cyclo[2]­malonate **2**

In a three necks, round-bottom flask, dried under inert atmosphere (nitrogen or argon), 5-iodo-1,3-benzendimethanol (132 mg, 0.5 mmol) is added and dissolved in anhydrous CH_2_Cl_2_ (25 mL). Anhydrous pyridine (92 μL, 1.15 mmol) is added in the flask. The other anhydrous CH_2_Cl_2_ (15 mL) is added in the dropping funnel with α-iodomalonyl dichloride (77.5 mg, 0.55 mmol). The solution is added dropwise within 4 h. After 16 h the reaction is washed three times with H_2_O. The organic phase is dried over Na_2_SO_4_ and evaporated. The crude reaction mixture is purified by flash column chromatography with cyclohexane-EtOAc 9:1 as mobile phase. The macrocyclic product is obtained as white solid with a yield of 20%. Mp 202–207 °C; ^1^H NMR: (600 MHz, CDCl_3_): δ 7.62 (s, 4H, CH Ar-_o_I), 7.28 (s, 2H, CH Ar-_p_I), 5.08 (s, 8H, CH_2_), 3.50 (s, 4H, CH_2_α); ^13^C NMR: (150 MHz, CDCl_3_): δ 165.92, 137.80, 137.46, 127.63, 94.13, 65.94, 41.82; IR-ATR: ν 2951, 2921, 2853, 1730, 1655, 1602, 1570, 1446 cm^–1^; HPLC-MS­(ESI): 7.68 min; mass calcd 663.91; found, 686.8 [M + Na]^+^, 702.9 [M + K]^+^.

### 4.4 Synthesis of cyclo[2]­malonate **3**

In a three necks, round-bottom flask, dried under inert atmosphere (nitrogen or argon), 1,3-benzendimethanol (69.1 mg, 0.5 mmol) is added and dissolved in anhydrous CH_2_Cl_2_ (25 mL). Anhydrous pyridine (92 μL, 1.15 mmol) is added in the flask. The other anhydrous CH_2_Cl_2_ (15 mL) is added in the dropping funnel with 2-phenylmalonyl dichloride (120 mg, 0.55 mmol). The solution is added dropwise within 4 h. After 16 h the reaction is washed three times with H_2_O. The organic phase is dried over Na_2_SO_4_ and evaporated. The crude reaction mixture is purified by flash column chromatography with cyclohexane-EtOAc 9:1 as mobile phase. The macrocyclic product is obtained as white solid with a yield of 35%. Mp 182–187 °C; ^1^H NMR (600 MHz, CDCl_3_): δ 7.45–7.24 (m, 18H, CH Ar), 5.23 and 5.21 (d, *J* = 12.3 Hz, 4 H, CH_2_O, mixture of diastereoisomers), 5.03 and 5.01 (d, *J* = 12.3 Hz, 4 H, CH_2_O, mixture of diastereoisomers), 4.75 and 4.76 (s, 2H, COCHPhCO, mixture of diastereoisomers); ^13^C NMR (150 MHz, CDCl_3_): δ 167.90, 135.74, 132.37, 129.65, 129.62, 128.88, 128.86, 128.81, 128.75, 128.64, 128.63, 128.42, 128.25, 67.37, 67.34, 58.09, 58.05; IR-ATR: ν 3022, 2928, 2855, 1726, 1498, 1454 cm^–1^; HPLC-MS: *Rt* = 11.063 min, mass calcd 564.18, mass, found; 587.0 [M + Na]+, 603.0 [M + K]^+^.

### 4.5 Synthesis of cyclo[2]­malonate **4**

In a three necks, round-bottom flask, dried under inert atmosphere (nitrogen or argon), 5-iodo-1,3-benzendimethanol (132 mg, 0.5 mmol) is added and dissolved in anhydrous CH_2_Cl_2_ (25 mL). Anhydrous pyridine (92 μL, 1.15 mmol) is added in the flask. The other anhydrous CH_2_Cl_2_ (15 mL) is added in the dropping funnel with 2-phenylmalonyl dichloride (120 mg, 0.55 mmol). The solution is added dropwise within 4 h. After 16 h the reaction is washed three times with H_2_O. The organic phase is dried over Na_2_SO_4_ and evaporated. The crude reaction mixture is purified by flash column chromatography with cyclohexane-EtOAc 95:5 as mobile phase. The macrocyclic product is obtained, after washing with hexane, as white solid with a yield of 35%. Mp: 191–194 °C; ^1^H NMR: (600 MHz, CDCl_3_): δ 7.61 and 7.59 (d, *J* = 1.5 Hz, 4H, CH o-I Ar, mixture of diastereoisomers), 7.42–7.39 (m, 10H, PhCH) 7.30 and 7.29 (s, 2H, CHAr, mixture of diastereoisomers), 5.19 and 5.17 (d, *J* = 12.5 Hz, 4H, CH_2_O, mixture of diastereoisomers), 4.99 and 4.97 (d, *J* = 12.5 Hz, 4H, CH_2_O, mixture of diastereoisomers), 4.77 and 4.75 (s, 2H, CHα, mixture of diastereoisomers); ^13^C NMR: (150 MHz, CDCl3): δ 166.19, 135.76, 128.86, 128.77, 128.45, 67.11, 42.01; IR-ATR: ν 2956, 2919, 2850, 1750, 1655, 1570, 1449 cm^–1^; HPLC-MS­(ESI): 10.05 min, mass calcd 815.97, found; 838.8 [M + Na]^+^

### 4.6 Synthesis of cyclo[2]­malonate **5**

In a three necks, round-bottom flask, dried under inert atmosphere (nitrogen or argon), 5-iodo-1,3-benzendimethanol (132 mg, 0.5 mmol) is added and dissolved anhydrous CH_2_Cl_2_ (25 mL). Anhydrous pyridine (92 μL, 1.15 mmol) is added in the flask. The other anhydrous CH_2_Cl_2_ (15 mL) is added in the dropping funnel with 2-(4-chlorophenyl)­malonyl dichloride (138 mg, 0.55 mmol). The solution is added dropwise within 4 h. After 16 h the reaction is washed three times with H_2_O. The organic phase is dried over Na_2_SO_4_ and evaporated. The crude reaction mixture is purified by flash column chromatography with cyclohexane-EtOAc 95:5 as mobile phase. The macrocyclic product is obtained as pale-yellow solid with yield of 15%. Mp 202–204 °C; ^1^H NMR: (600 MHz, CDCl_3_): δ 7.60 and 7.58 (d, 4H, *J* = 1.3 Hz, C–H Ar, mixture of diastereoisomers), 7.39–7.33 (m, 8H, C–H *p*Cl–Ar), 7.28 and 7.26 (s, 2H, C–H Ar, mixture of diastereoisomers), 5.19 and 5.17 (d, 4H, *J* = 12.4, CH_2_O, mixture of diastereoisomers), 4.97 and 4.95 (d, 4H, *J* = 12.4, CH_2_O, mixture of diastereoisomers), 4.72 and 4.71 (s, 2H, CHα, mixture of diastereoisomers); ^13^C NMR: (150 MHz, CDCl_3_): δ 167.17, 137.52, 137.46, 137.33, 134.82, 130.83, 130.31, 129.06, 127.48, 94.07, 66.21, 57.15; IR-ATR: ν 2955, 2920, 2851, 1750, 1724, 1654, 1570, 1490, 1459 cm^–1^; HPLC-MS­(ESI): 11.24 min; mass calcd 883.9, found; 906.8 [M + Na]^+^, 922.6 [M + K]^+^.

### 4.7 Synthesis of cyclo[2]­malonate **6**

In a three necks, round-bottom flask, dried under inert atmosphere (nitrogen or argon), 5-iodo-1,3-benzendimethanol (132 mg, 0.5 mmol) is added and dissolved in anhydrous CH_2_Cl_2_ (25 mL). Anhydrous pyridine (92 μL, 1.15 mmol) is added in the flask. The other anhydrous CH_2_Cl_2_ (15 mL) is added in the dropping funnel with 2-benzylmalonyl dichloride (127 mg, 0.55 mmol). The solution is added dropwise within 4 h. After 16 h the reaction is washed three times with H_2_O. The organic phase is dried over Na_2_SO_4_ and evaporated. The crude reaction mixture is purified by flash column chromatography with cyclohexane-EtOAc 9:1 as mobile phase. The macrocyclic product is obtained as white solid with yield of 19%. Mp 179–183 °C; ^1^H NMR: (600 MHz, CDCl_3_): δ 7.53 (s, 4H, C-HAr), 7.32–7.30 (m, 4H, C–H ArBn), 7.26–7.24 (m, 2H, C–H ArBn), 7.23–7.21 (m, 4H, C–H ArBn), 7.08 (s, 2H, C–H Ar), 5.09 and 5.07 (d, 4H, *J* = 12.5 Hz, CH_2_O, mixture of diastereoisomers), 4.87 and 4.85 (d, 4H, *J* = 12.5 Hz, CH_2_O), 3.79 and 3.78 (t, 2H, *J* = 7.8 Hz, CHα, mixture of diastereoisomers), 3.27 and 3.26 (d, 4H, *J* = 7.8 Hz, CH_2_Bn, mixture of diastereoisomers); ^13^C NMR: (150 MHz, CDCl_3_): δ 168.16, 137.58, 137.45, 137.15, 128.85, 127.11, 127.00, 94.13, 65.71, 53.72, 34.28; IR-ATR: ν 3061, 3025, 2955, 2920, 2850, 1748, 1722, 1603, 1570, 1452 cm^–1^; HPLC-MS­(ESI): 10.932 min; mass calc. 844.44, found; 866.8 [M + Na]^+^, 882.8 [M + K]^+^.

### 4.8 Synthesis of cyclo[2]­malonate **7**

In a three necks, round-bottom flask, dried under inert atmosphere (nitrogen or argon), macrocycle **4** (130 mg, 0.16 mmol) is added, followed by Pd­(PPh_3_)_2_Cl_2_ (7 mg, 0.016 mmol) and CuI (6.1 mg, 0.032 mmol). After 3 rounds of vacuum-N_2,_ anhydrous THF (6 mL, 0.02 M), trimethylsilylacetylene (220 μL, 1.6 mmol) and anhydrous TEA (334 μL, 2.4 mmol) are added. The reaction is stirred for 16 h at rt. When the starting disappears (monitored by TLC) TBAF 1 M in THF (2.4 mL, 2.4 mmol) are slowly added. Precipitate is formed in the reaction mixture and it is stirred at rt for 1.5 h. The mixture is diluted with H_2_O until precipitate solubilization. EtOAc is added and the organic phase is washed first with NH_4_Cl (sat.) x2, then with H_2_Ox2. The mixture dried over Na_2_SO_4_ and purified by flash chromatography column with cyclohexane-EtOAc 9:1 as mobile phase, to obtain a white solid with 73% yield. Mp 186–190 °C; ^1^H NMR: (600 MHz, CDCl_3_): δ 7.44–7.37 (m, 14H, C–H Ar, C–H Ar_α_), 7.32 and 7.31 (s, 2H, C–H Ar, mixture of diastereoisomers), 5.21 and 5.2 (d, 4H, *J* = 12.4 Hz, CH_2_O, mixture of diastereoisomers), 5.01 (d, 4H, *J* = 12.4 Hz, CH_2_O), 4.77 and 4.75 (s, 2H, CHαPh, mixture of diastereoisomers), 3.10 and 3.09 (s, 2H, CCH, mixture of diastereoisomers); ^13^C NMR: (150 MHz, CDCl_3_): δ 167.61, 135.97, 132.14, 132.03, 129.52, 128.81, 128.65, 128.61, 122.81, 82.52, 78.31, 66.59, 57.92, 26.93; IR-ATR: ν 3275, 2955, 2920, 2850, 1735, 1560, 1456 cm^–1^; HPLC-MS­(ESI): 8.85 min; mass calcd 612.27, found; 635 [M + Na]^+^.

### 4.9 General Method for the Macrocycle Deuteration

A 10 mL two-neck round-bottom flask was charged with the macrocycle (10 mg, 1 equiv) and KO*t*Bu (4 equiv) dissolved in dry 1,4-dioxane (2 mL) under an argon atmosphere. The reaction mixture was stirred for 3 h, then 35% DCl in D_2_O (4.5 equiv) was added. The stirring continued for an additional 30 min, then the solvent was removed to dryness using a rotary evaporator followed by high vacuum. The crude reaction mixture was dissolved in CDCl_3_ to record the ^1^H NMR spectrum.

### 4.10 Preparation of VO­(NO_3_)_2_

In a 50 mL single-neck round-bottom flask, Ba­(NO_3_)_2_ (0.50 mmol, 130.67 mg) and VO­(SO_4_) (0.25 mmol, 63.27 mg) were combined and dissolved in deionized water (15 mL). The resulting mixture was stirred at room temperature for 1.5 h. Upon completion, the suspension was filtered to remove the insoluble byproduct, and the filtrate was concentrated to dryness under reduced pressure using a rotary evaporator. During solvent removal, a gradual color change from light blue to brown was observed, consistent with the formation of the desired vanadyl nitrate species. The resulting crude solid was further dried under high vacuum, employing a liquid nitrogen trap, to afford the product as a brownish solid (38.5 mg, 81% yield).

### 4.11 General Method for the Preparation of Macrocycles Vanadyl Complexes

A 10 mL two-neck round-bottom flask was flame-dried under vacuum, followed by three vacuum–argon cycles to ensure anhydrous and inert conditions. After cooling to room temperature, the flask was charged with the selected macrocycle (6 μmol), followed by the addition of anhydrous solvent (3.5 mL). Potassium *tert*-butoxide (KOtBu, 24 μmol, 2.70 mg) was then introduced under a dry argon atmosphere. The system was further degassed by two additional vacuum–argon cycles without heating and the reaction mixture was stirred at room temperature for 4 h. During this period, the initially pale-yellow solution gradually developed a slightly deeper yellow coloration. After 4 h, vanadyl nitrate (VO­(NO_3_)_2_) (8.0 equiv. 48 μmol, 9.26 mg) was added to the reaction mixture, causing an immediate color change to brown. The reaction was allowed to proceed under stirring 96 h at room temperature. Then the solvent was removed under reduced pressure using a rotavapor. The flask was purged again with argon through additional vacuum–argon cycles and sealed with parafilm to prevent exposure to air, as the product was found to be air and water-sensitive. MALDI-TOF analysis was performed to confirmed whether the formation of the vanadyl complex takes place.

Crystallization experiments:

- Crystals of macrocycle **1** suitable for analysis were obtained by slow evaporation of a solution of the compound (10 mg) in CH_2_Cl_2_ (5 mL).
- Macrocycle **2** (10 mg) was dissolved in hot EtOAc (5 mL) and allowed to stand for several days to afford crystals.
- Macrocycle **3** (10 mg) was dissolved in hot THF (5 mL), and crystals were obtained by slow evaporation of the solvent.
- Macrocycles **4** and **5** were purified by column chromatography and subsequently crystallized by slow evaporation from cyclohexane/EtOAc (9:1 and 95:5, respectively).
- Macrocycle **6** (10 mg) was dissolved in hot EtOAc (5 mL) and allowed to stand for several days to afford crystals.
- Macrocycle **7** was purified by column chromatography and crystallized by slow evaporation from cyclohexane/EtOAc (9:1).

### 4.12 Single Crystal X-ray Diffraction (SCXRD) and Structural Analysis

Single-crystal XRD data for compounds **1**-**4** were collected, at room temperature (RT), on an Oxford X’Calibur S CCD diffractometer equipped with a graphite monochromator (Mo Kα radiation, λ = 0.71073 Å), while data collections for compounds **5**-**7** were collected, at room temperature, on a Bruker D8 Venture diffractometer, equipped with a PHOTON III detector and an IμS 3.0 microfocus X-ray source (Mo Kα radiation, λ = 0.71073 Å). All the structures were solved with SHELXT by intrinsic phasing^55^ and refined on *F* ^*2*^ with SHELXL^56^ implemented in the Olex2 software^57^ by full-matrix least-squares refinement. H_CH_ atoms for all compounds were added in calculated positions and refined by riding on their on their respective carbon atoms. All non-hydrogen atoms were refined anisotropically, and rigid-bond RIGU restraints were applied.^58^ Data collection and refinement details are listed in Table Supporting Information 1. The Mercury^59^ and CylView^60^ programs were used for molecular graphics, while the CageCavityCalc (C3) software^61^ was used to evaluate the cavity size for each macrocycle; to this end, the grid size was adapted to 0.5 Å for better precision. For crystalline **7** (a solid solution of *cis*/*trans* isomers), two separate structural models were generated from the corresponding CIF by isolating the *cis* and *trans* isomers, and were subsequently used for the evaluation of the cavity size. The same approach was applied to the computation of Intermolecular Interaction Energies (IIEs),^50^ obtained as the sum of electrostatic, polarization, dispersion, and repulsion contributions, using CrystalExplorer package.^51^ The calculations were performed with the CE-B3LYP/6-31G­(d,p) energy model (*k*_ele_ = 1.019; *k*_pol_ = 0.651; *k*_disp_ = 0.901; *k*_rep_ = 0.811).

## Supplementary Material





## Data Availability

The data underlying this study are available in the published article and its online Supporting Information. Raw data comprising NMR FID, FT-IR and HPLC-MS of the compounds reported are openly available in AMSActa Institutional Research Repository DOI: https://doi.org/10.6092/unibo/amsacta/8989. Crystal data can be obtained free of charge via www.ccdc.cam.ac.uk/conts/retrieving.htmL (or from the Cambridge Crystallographic Data Centre, 12 Union Road, Cambridge CB21EZ, UK; fax: (+44)­1223-336-033; or e-mail: deposit@ccdc.cam.ac.uk). CCDC codes: 2532272–2532278.

## Notes

MC_1-7 (CIF)
Schemes and procedures for the synthesis of (5-iodo-1,3-phenylene)­dimethanol, malonyl dichlorides and trimethylsilyl macrocycle (Me_3_Si)_2_-**7**; ^1^H NMR, ^13^C NMR, COSY, HSQC, IR-ATR, spectra and HPLC-MS analysis of **1**–**7**; HPLC analysis of the crude reaction of macrocyclization for the formation of **1**–**6**; Details of the ^1^H NMR spectra of **1**–**6** before and after deuteration; Crystal data and refinement details for compounds **1**–**7** collected at room temperature (293–300 K); Asymmetric unit of compound **7**; ^1^H NMR spectra of dissolved crystals obtained from macrocycle **7**; Crystal packing diagrams of macrocycles **1**–**7** (PDF)
