# Supplementary material for: Synthesis, Structural Characterization and Reactivity of Macrocyclic Cyclo[2]malonates
Source: ACS Omega. 2026 Jun 8;11(24):36219–29. doi: 10.1021/acsomega.6c03626 (PMC13294901; doi:10.1021/acsomega.6c03626)
Supplement: Supplementary file 2 [file ao6c03626_si_002.pdf]

SUPPORTING INFORMATION

Synthesis, Structural Characterization and Reactivity of Macrocyclic Cyclo[2]malonates

Samuele Ruffoli,[a] Andrea Vitale,[a] Kevin D'Addazio,[a] Alessandro Pispero,[a] Daniele Sartore,[a] Demetra Giuri,[a]*Claudia Tomasini,[a]* Simone D'Agostino[a]*

[a] *Dipartimento di Chimica Giacomo Ciamician and INSTM Research Unit, Università di Bologna, Via Piero Gobetti, 85, 40129 Bologna, Italy*

email: demetra.giuri2@unibo.it; claudia.tomasini@unibo.it; simone.dagostino2@unibo.it

**Scheme S1.** Scheme of the synthesis of (5-iodo-1,3-phenylene)dimethanol	Page S3
Procedure for the synthesis of (5-iodo-1,3-phenylene)dimethanol	Page S3
**Scheme S2.** Scheme of the synthesis of 2-phenylmalonyl dichloride, 2-(4-chlorophenyl)malonyl dichloride and 2-benzylmalonyl dichloride	Page S4
Procedure for the synthesis of 2-phenylmalonyl dichloride, 2-(4-chlorophenyl)malonyl dichloride and 2-benzylmalonyl dichloride	Page S4
**Scheme S3.** Scheme of the synthesis of trimethylsylil macrocycle **$(Me_3Si)_2$-7**	Page S5
Procedure for the synthesis of trimethylsylil macrocycle **$(Me_3Si)_2$-7**	Page S5
**Figures S1-S6.** ^{1}H NMR, ^{13}C NMR, COSY, HSQC, IR-ATR, spectra and HPLC-MS analysis of **1**	Pages S6-S8
**Figures S7-S12.** ^{1}H NMR, ^{13}C NMR, COSY, HSQC, IR-ATR, spectra and HPLC-MS analysis of **2**	Pages S9-S11
**Figures S13-S18.** ^{1}H NMR, ^{13}C NMR, COSY, HSQC, IR-ATR, spectra and HPLC-MS analysis of **3**	Pages S12-S14
**Figures S19-S24.** ^{1}H NMR, ^{13}C NMR, COSY, HSQC, IR-ATR, spectra and HPLC-MS analysis of **4**	Pages S15-S17
**Figures S25-S30.** ^{1}H NMR, ^{13}C NMR, COSY, HSQC, IR-ATR, spectra and HPLC-MS analysis of **5**	Pages S18-S20
**Figures S31-S36.** ^{1}H NMR, ^{13}C NMR, COSY, HSQC, IR-ATR, spectra and HPLC-MS analysis of **6**	Pages S21-S23
**Figures S37-S39.** ^{1}H NMR, and COSY spectra of **$(Me_3Si)_2$-7**	Pages S24-S25
**Figures S40-S45.** ^{1}H NMR, ^{13}C NMR, COSY, HSQC, IR-ATR, spectra and HPLC-MS analysis of **7**	Pages S26-S28
**Tables S1-S6.** HPLC analysis of the crude reaction of macrocyclization for the formation of **1-6**	Pages S29-S34
**Table S7.** Crystal data and refinement details for compounds **1-7** collected at room temperature (293-300K).	Page S35
**Figure S46.** Asymmetric unit of compound **7**	Page S36

**Figure S47.** ^{1}H NMR spectra of dissolved crystals obtained from macrocycle **7**	Page S36
**Figure S48.** Crystal packing diagrams of macrocycles **1-7**	Page S37
**Table S8.** Electrostatic, polarization, dispersion, repulsion terms and total interaction energy within the pairs of molecules (a) **7-cis** and (b) **7-trans**.	Page S38
**Figure S49.** Details of the ^{1}H NMR spectra of **1** before and after deuteration.	Page S39
**Figure S50.** Details of the ^{1}H NMR spectra of **2** before and after deuteration.	Page S40
**Figure S51.** Details of the ^{1}H NMR spectra of **3** before and after deuteration.	Page S41
**Figure S52.** Details of the ^{1}H NMR spectra of **4** before and after deuteration.	Page S42
**Figure S53.** Details of the ^{1}H NMR spectra of **5** before and after deuteration.	Page S43
**Figure S54.** Details of the ^{1}H NMR spectra of **6** before and after deuteration.	Page S44
**Figure S55.** Details of the ^{1}H NMR spectra of **7** before and after deuteration.	Page S45
**Figures S56-S62.** MALDI-TOF analysis of macrocycles vanadyl complexes from **1-7**	Pages S46-S52

Synthesis of intermediates: all the intermediates used as starting material for the macrocycle synthesis are prepared following literature procedure and the data analysis match the literature data.

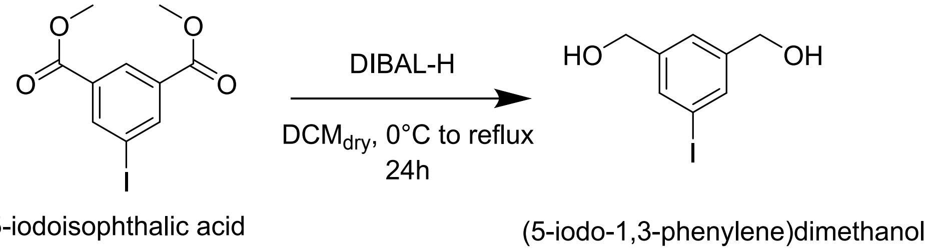

Scheme S1. Reagents and conditions: Dimethyl 5-iodoisophthalate (1 equiv.), DIBAL-H 1 M in hexane (5 equiv.), dry DCM, 0 °C to reflux, 24 h.

Procedure for the synthesis of (5-iodo-1,3-phenylene)dimethanol - A 250 ml three-neck round-bottom flask equipped with a dropping funnel and a reflux condenser was flame-dried and flushed with nitrogen. Inside the flask was placed 2 5-iodoisophthalic acid (6.21 mmol, 2.0 g) in dry CH_2Cl_2 (30 mL). In the dropping funnel, a 1 M solution of DIBAL–H in hexane (17.25 mL) was placed. The DIBAL–H solution was added dropwise to the reaction mixture over 1 hour while cooling with an ice–salt bath (-20 °C). After the addition was complete, the mixture was heated to reflux at 50 °C and stirred for 6 hours, then allowed to react at room temperature for an additional 16 hours. The solution became completely colorless. The reaction was quenched by the slow addition of 5 M hydrochloric acid under cooling with an ice bath, forming a fine solid residue in the flask. Organic solvents were removed under reduced pressure using a rotary evaporator (condensation observed in the bump trap). The aqueous phase was extracted with ethyl acetate, and the combined organic layers were washed with water, dried over anhydrous sodium sulfate, filtered, and concentrated under reduced pressure. The crude product was further dried under high vacuum over a silica plug. Purification was per formed by column chromatography on silica gel using cyclohexane/ethyl acetate (Cy:AcOEt = 3:2) as the eluent. Obtaining the desired product as white solid with yield of 89%.

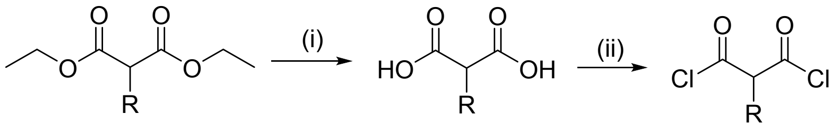

2-phenylmalonyl dichloride: R = Ph
2-(4-chlorophenyl)malonyl dichloride: R =4-Cl-Ph
2-benzylmalonyl dichloride: R = Bn

Scheme S2. Reagents and conditions: (i): Malonic ester (1 equiv.), NaOH 1M (4 equiv.), H_2O:MeOH 3:2, 16 h, rt. (ii): Malonic acid (1 equiv.) thionyl chloride (5 equiv.), reflux, 4 h.

Synthesis of malonic acids: In a 250 mL round bottomed flask, diethyl 2-phenylmalonate, diethyl 2-4-chlorophenyl)malonate, or diethyl 2-benzylmalonate (8 mmol) were dissolved in methanol (20 mL) and aqueous NaOH 1 M (32 mL). The mixture was stirred at room temperature for 16 hours. Methanol was then removed under reduced pressure using a rotary evaporator. The aqueous layer was washed with ethyl acetate (AcOEt) to remove unreacted starting material and acidified with 5 M hydrochloric acid (HCl) to pH ≈1. The precipitated acid was extracted with AcOEt, dried over a hydrous Na_2SO_4, filtered, and concentrated under reduced pressure, followed by high-vacuum drying. The corresponding malonic acids were obtained as white solids with quantitative yields.

Synthesis of malonyl dichlorides: In a two-neck round-bottom flask equipped with a reflux condenser, 2-phenylmalonic acid, 2-(4-chlorophenyl)malonic acid or 2-benzylmalonic acid (3 mmol) was dissolved in 1 mL of thionyl chloride ($SOCl_2$). The mixture was refluxed for 4 hours under gentle stirring. After completion, the reaction mixture was concentrated under reduced pressure using a rotary evaporator, followed by extended high-vacuum drying to remove residual $SOCl_2$. The resulting malonyl dichlorides were obtained as a yellow liquid and used directly in the subsequent reaction without further purification.

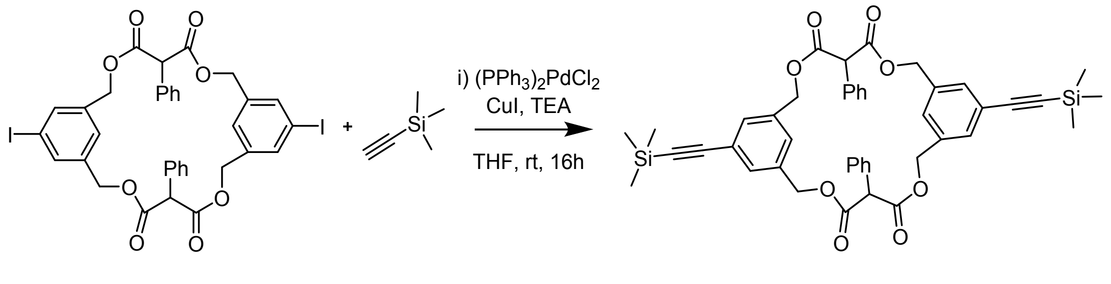

Scheme S3. Reagents and conditions: i) **4** (1 equiv.), alkine (10 equiv.), $(PPh_3)_2PdCl_2$ (0.1 equiv.), CuI (0.2 equiv.) TEA (15 equiv.) THF dry, rt, 16 h.

Synthesis of intermediate trimethylsylil macrocycle $(Me_3Si)_2$-7: In a three necks, round bottom flask, dried under N_2, 130 mg of macrocycle **4** (1 equiv. 816.38 g/mol, 0.16 mmol) are added, followed by 7 mg of $Pd(PPh_3)_2Cl_2$ (0.1 equiv. 439.16 g/mol, 0.016 mmol) and 6.1 mg of CuI, (0.2 equiv. 190.45 g/mol, 0.032 mmol). After 3 rounds of vacuum-N_2, 6 ml of THF (0.02 M), 220 µl of trimethylsilylacetylene (10 equiv. 98.22 g/mol, 1.6 mmol, 157.2 mg, d = 0.709 g/cm^3) and 334 µl of dry TEA (15 equiv. 101.19 g/mol, 2.4 mmol, 243 mg, d = 0.728 g/cm^3) are added. The reaction is stirred for 16 h at rt, then quenched with H_2O, diluted with AcOEt and washed first with NH_4Cl sat. x2, then with H_2O x2. After anidrification with Na_2SO_4, the solvent is evaporated and the crude purified by flash chromatography on silica gel with mobile phase 95:5 Cy:AcOEt. The product is obtained as pale brown film with yield of 80%. ^{1}H-NMR: (400 MHz, $CDCl_3$) δ = 7.45-7.36 (m, 14H, C-H Ar_α), 7.29 and 7.28 (t, 2H, J = 1.45 Hz, C-H Ar, mixture of diastereoisomers), 5.20 and 5.19 (d, 4H, J=12.34 Hz, CH_2O, mixture of diastereoisomers), 5.02 and 5.01 (d, 4H, J=12.34 Hz, CH_2O, mixture of diastereoisomers), 4.76 and 4.75 (s, 2H, CHPh, mixture of diastereoisomers), 0.25 (s, 9H, $Si(CH_3)_3$, mixture of diastereoisomers); ^{13}C-NMR: (150 MHz, $CDCl_3$) δ = 166.63, 134.67, 131.09, 130.64, 128.50, 127.73, 127.50, 126.33, 123.74, 90.63, 78.55, 65.78, 56.88, 29.67, 20.99, 18.03, 12.61; IR-ATR: ν = 3063, 3031, 2955, 2925, 2855, 1730, 1654, 1601, 1570, 1498, 1456 cm^{-1}.

Figure S1. ^{1}H-NMR spectrum of **macrocycle 1**

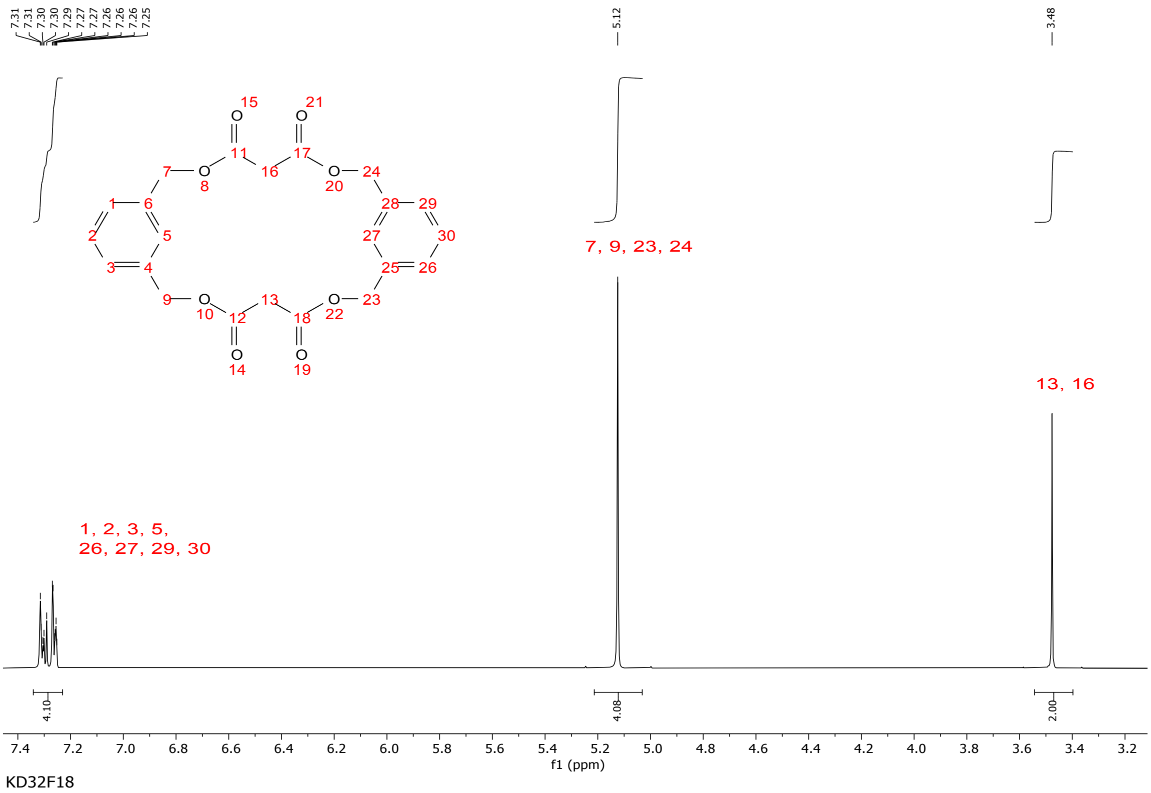

Figure S2. COSY-NMR spectrum of **macrocycle 1**

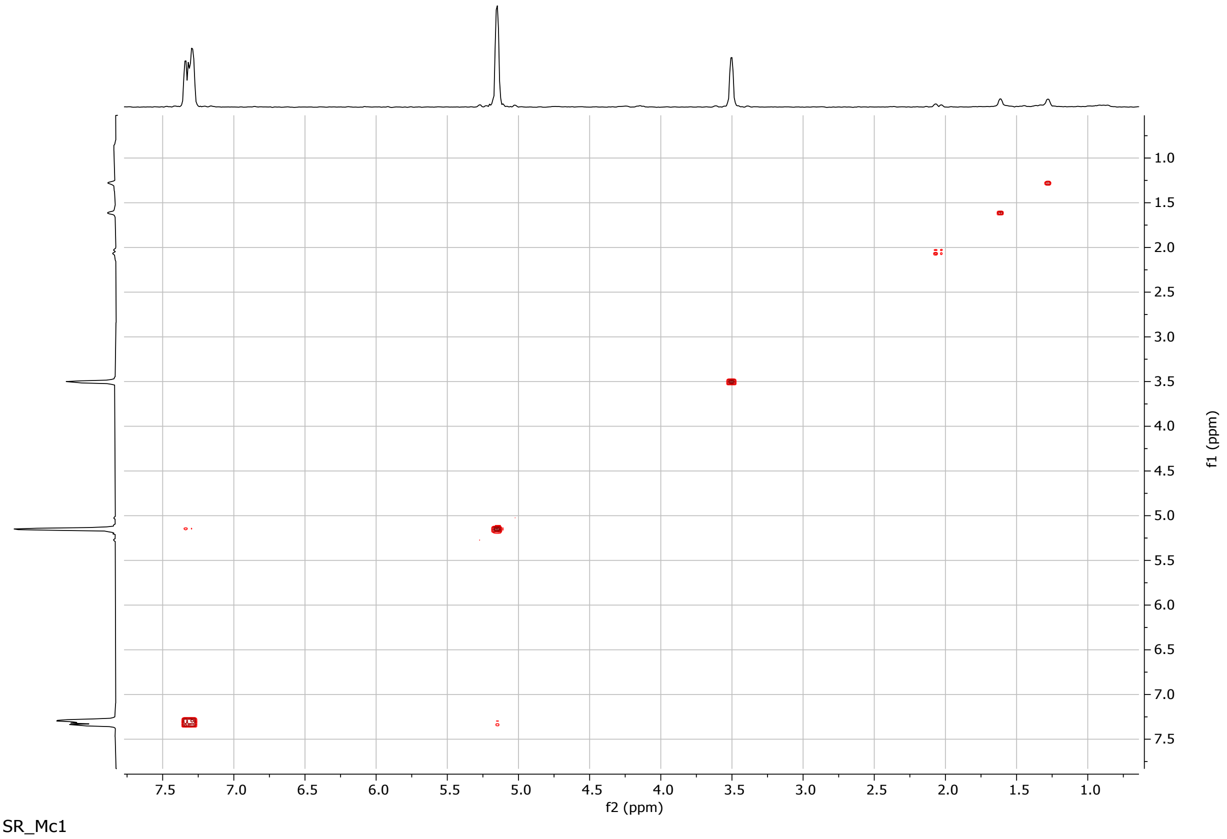

Figure S3. ^{13}C-NMR spectrum of **macrocycle 1**

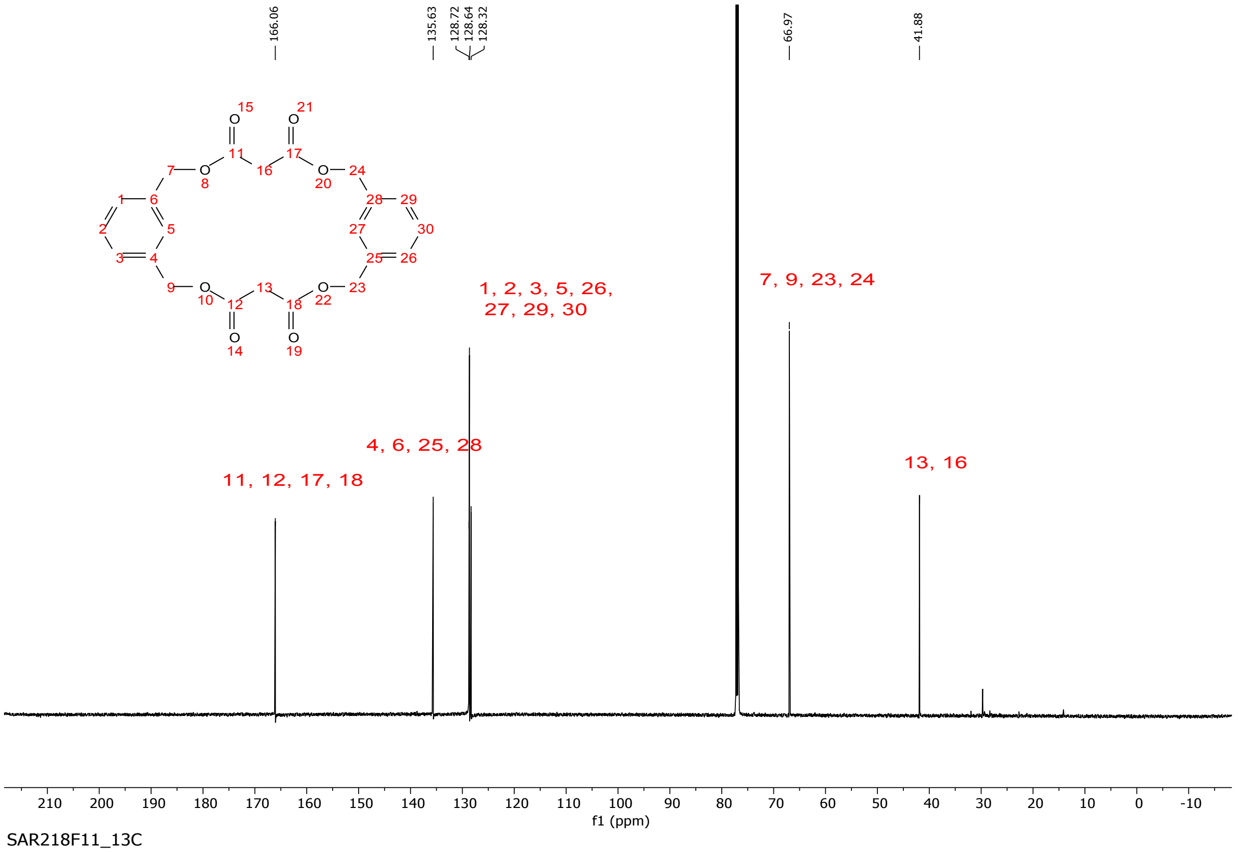

Figure S4. HSQC-NMR spectrum of **macrocycle 1**

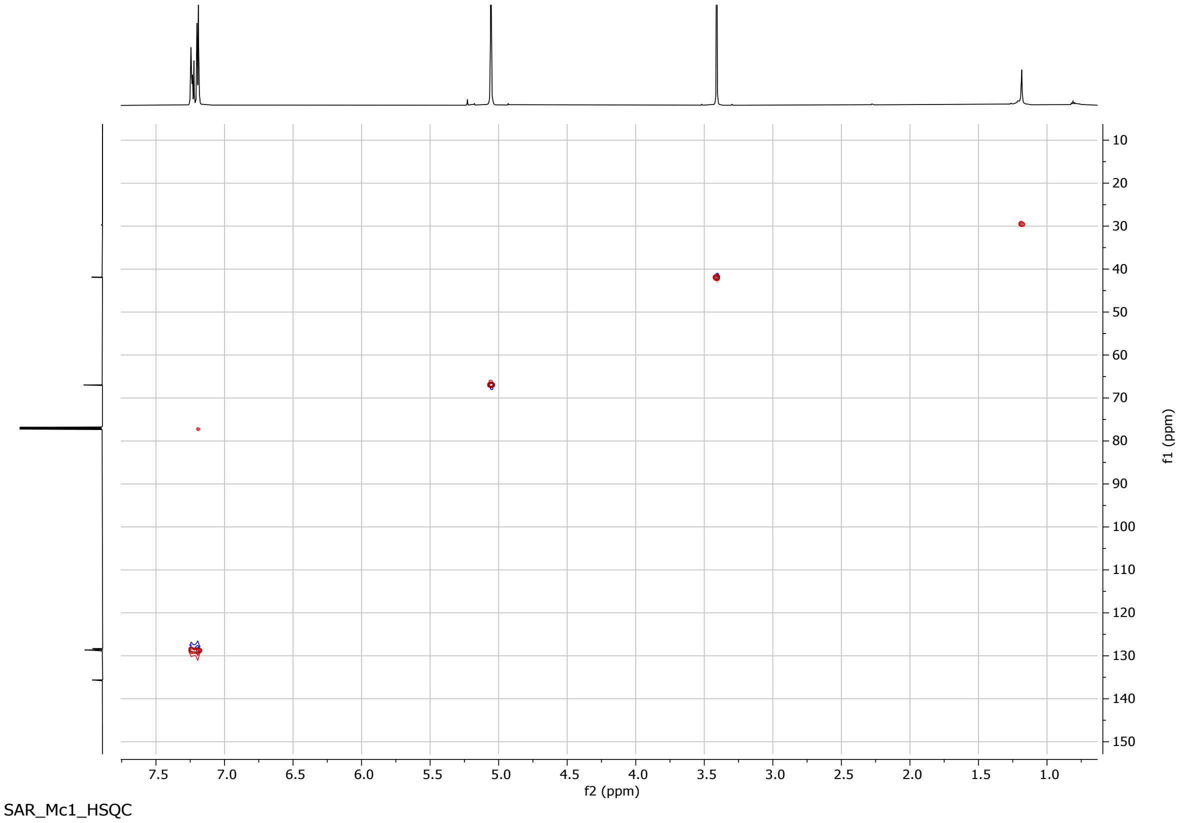

Figure S5. IR spectrum of **macrocycle 1**

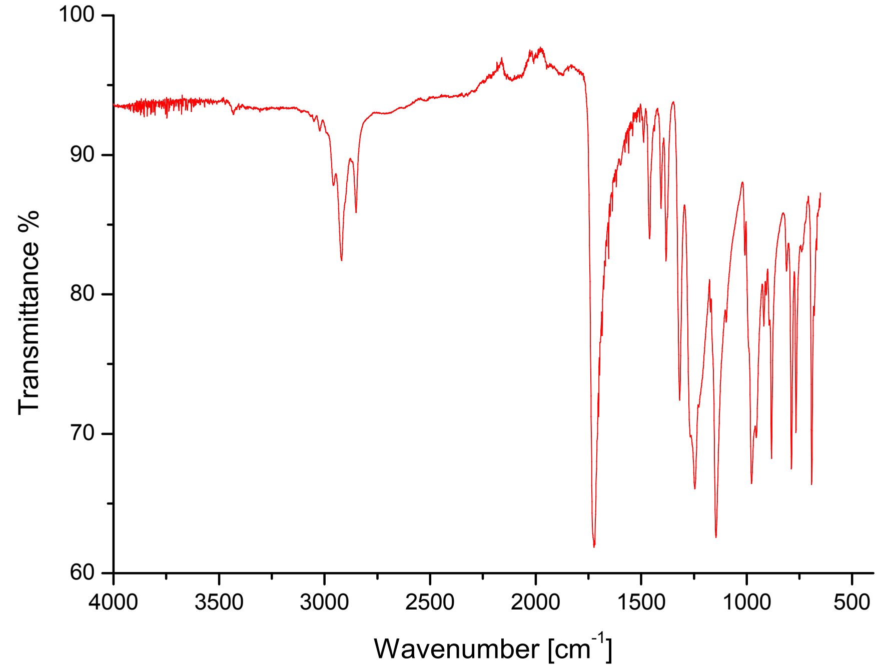

Figure S6. HPLC-MS analysis of **macrocycle 1**

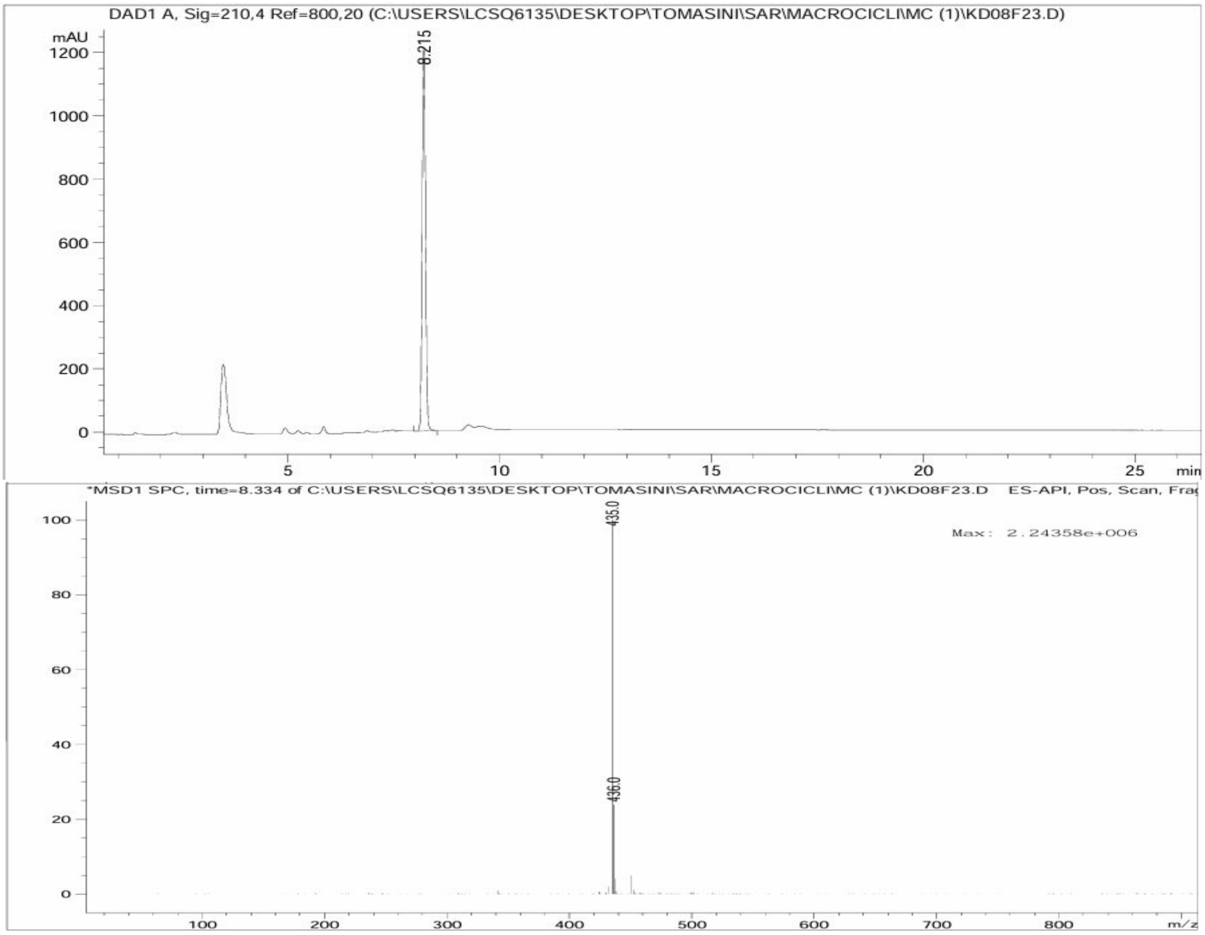

Figure S7. ^{1}H-NMR spectrum of **macrocycle 2**

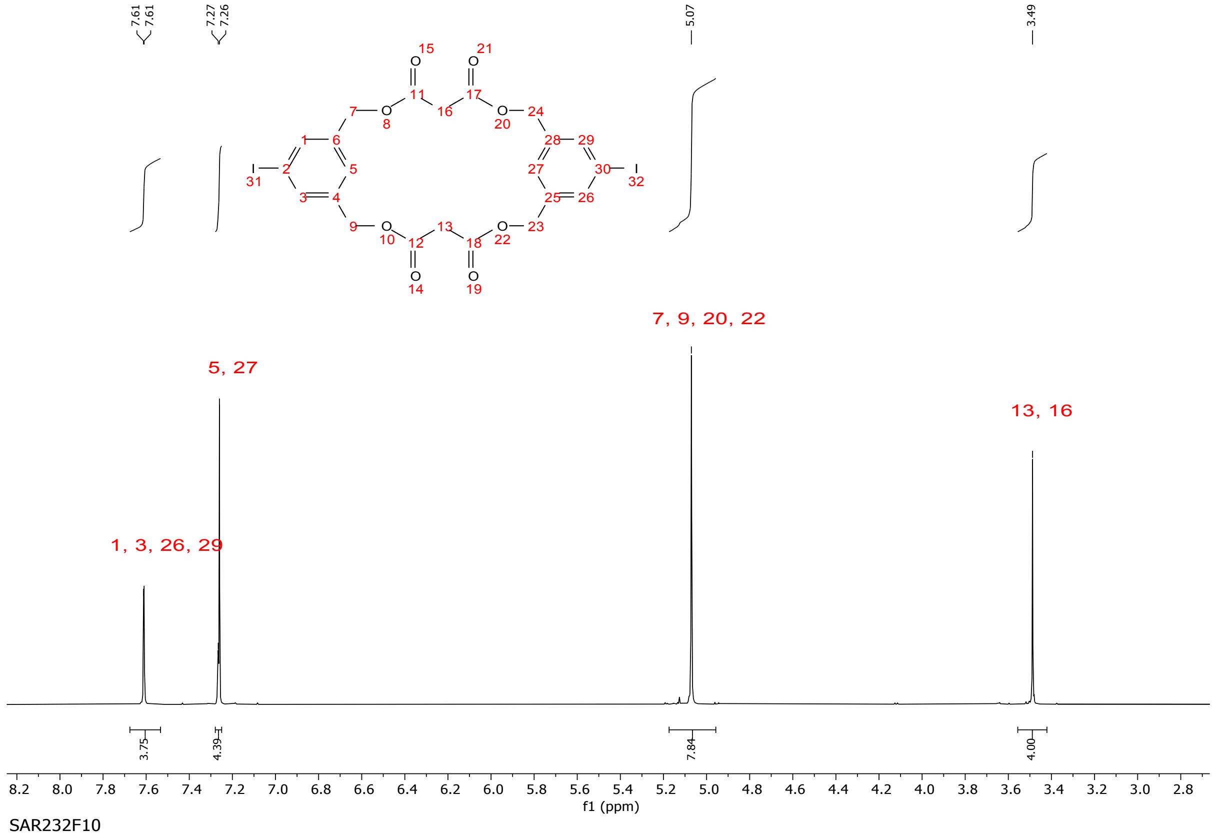

Figure S8. COSY-NMR spectrum of **macrocycle 2**

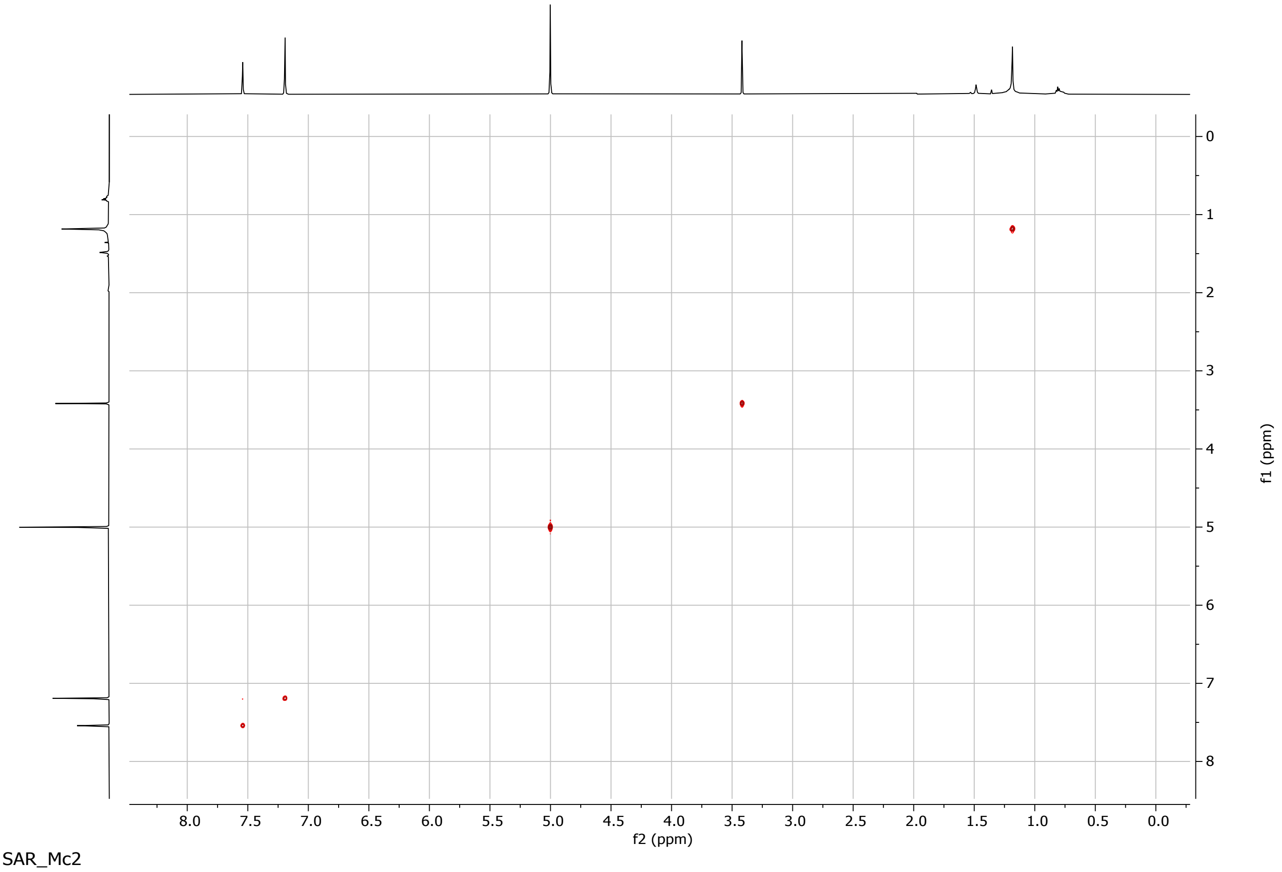

Figure S9. ^{13}C-NMR spectrum of **macrocycle 2**

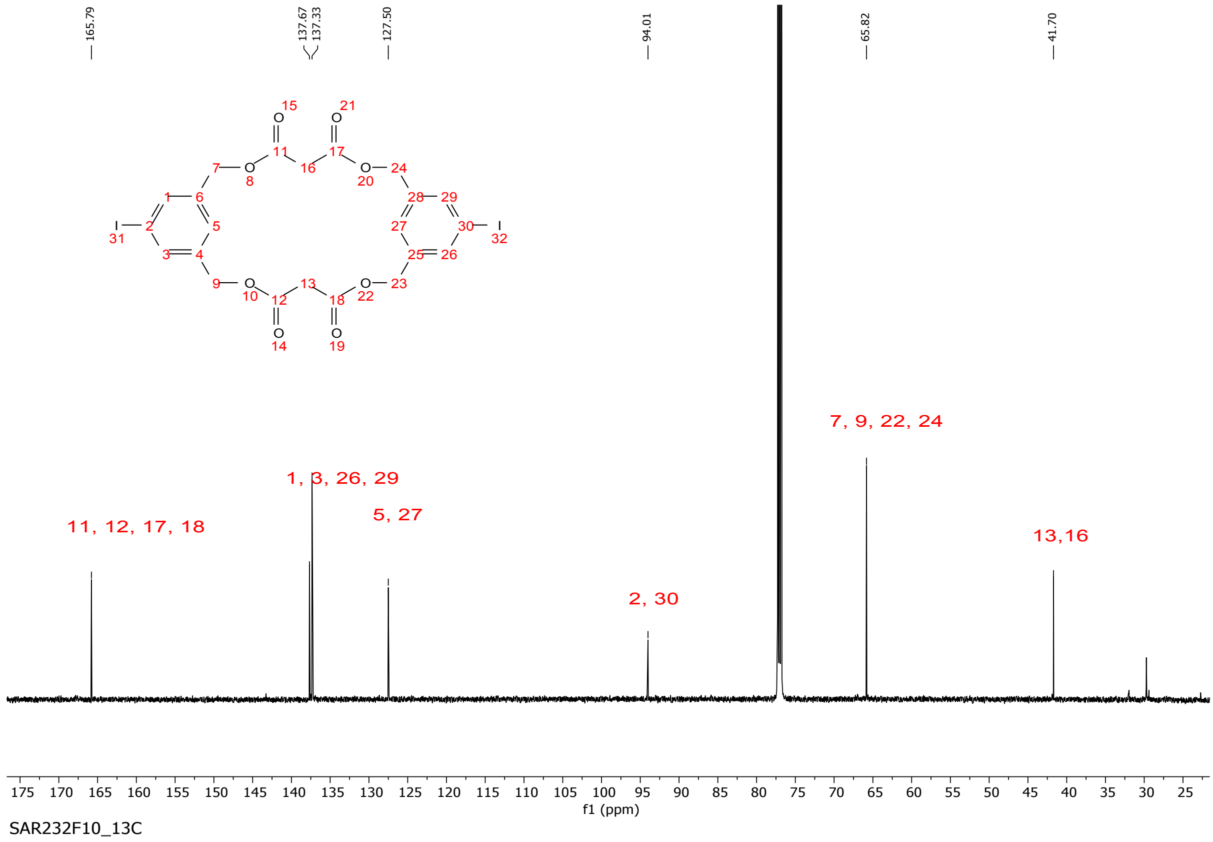

Figure S10. HSQC-NMR spectrum of **macrocycle 2**

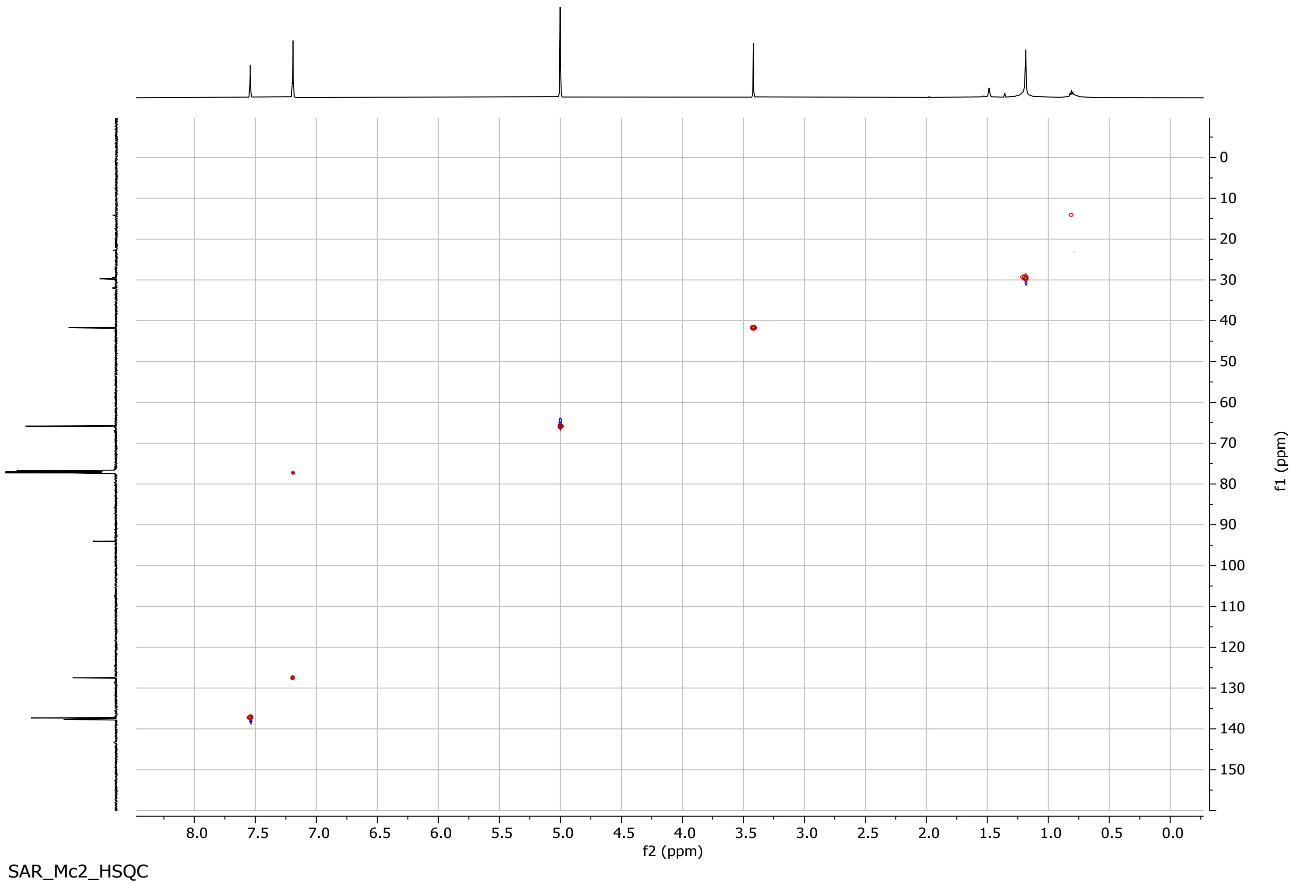

Figure S11. IR spectrum of **macrocycle 2**

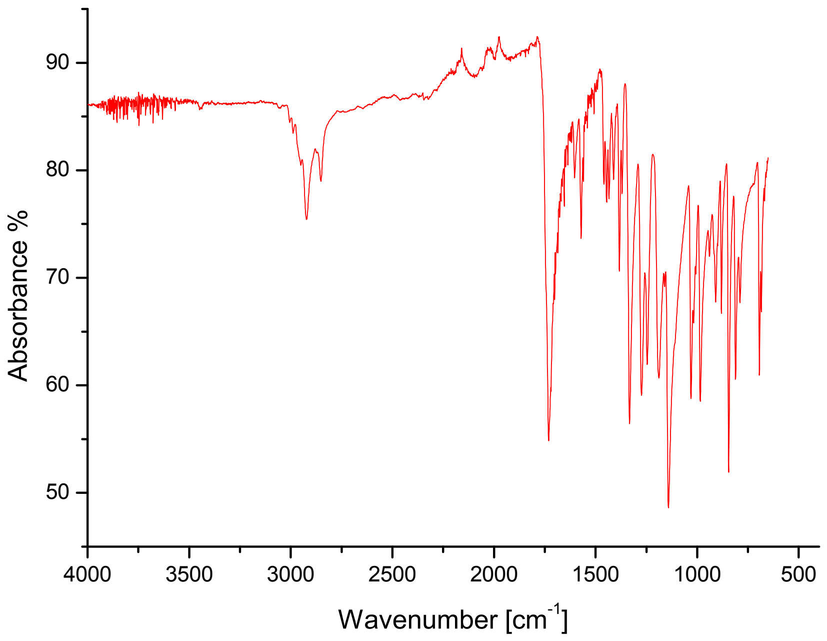

Figure S12. HPLC-MS analysis of **macrocycle 2**

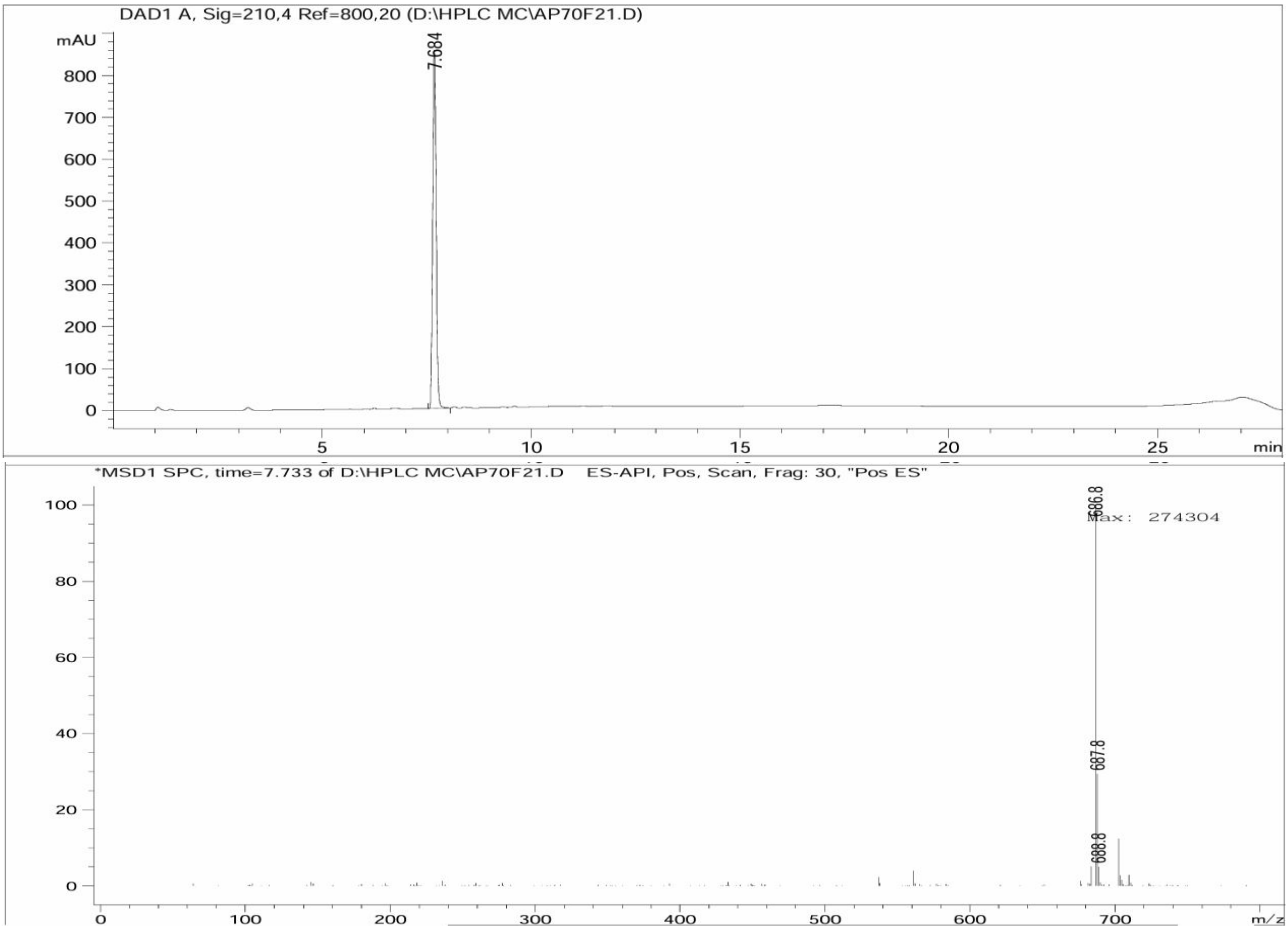

Figure S13. ^{1}H-NMR spectrum of **macrocycle 3**

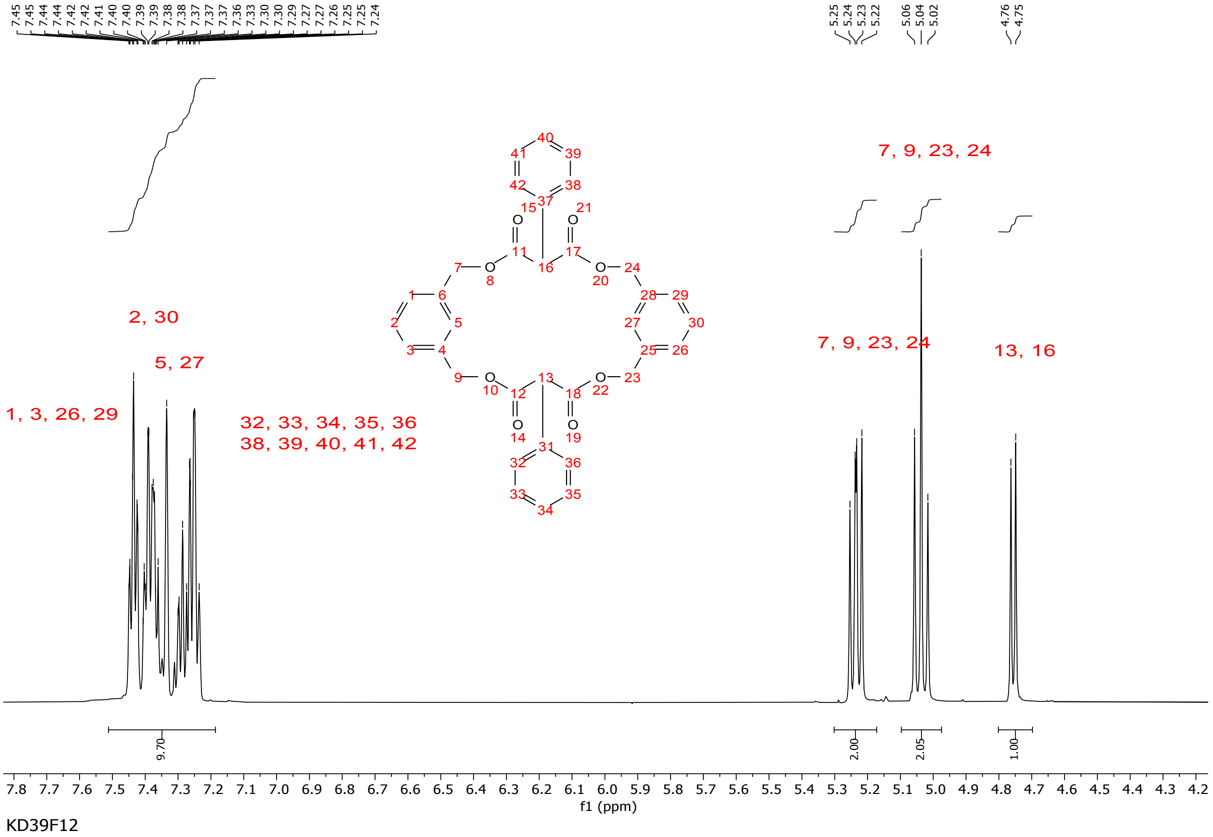

Figure S14. COSY-NMR spectrum of **macrocycle 3**

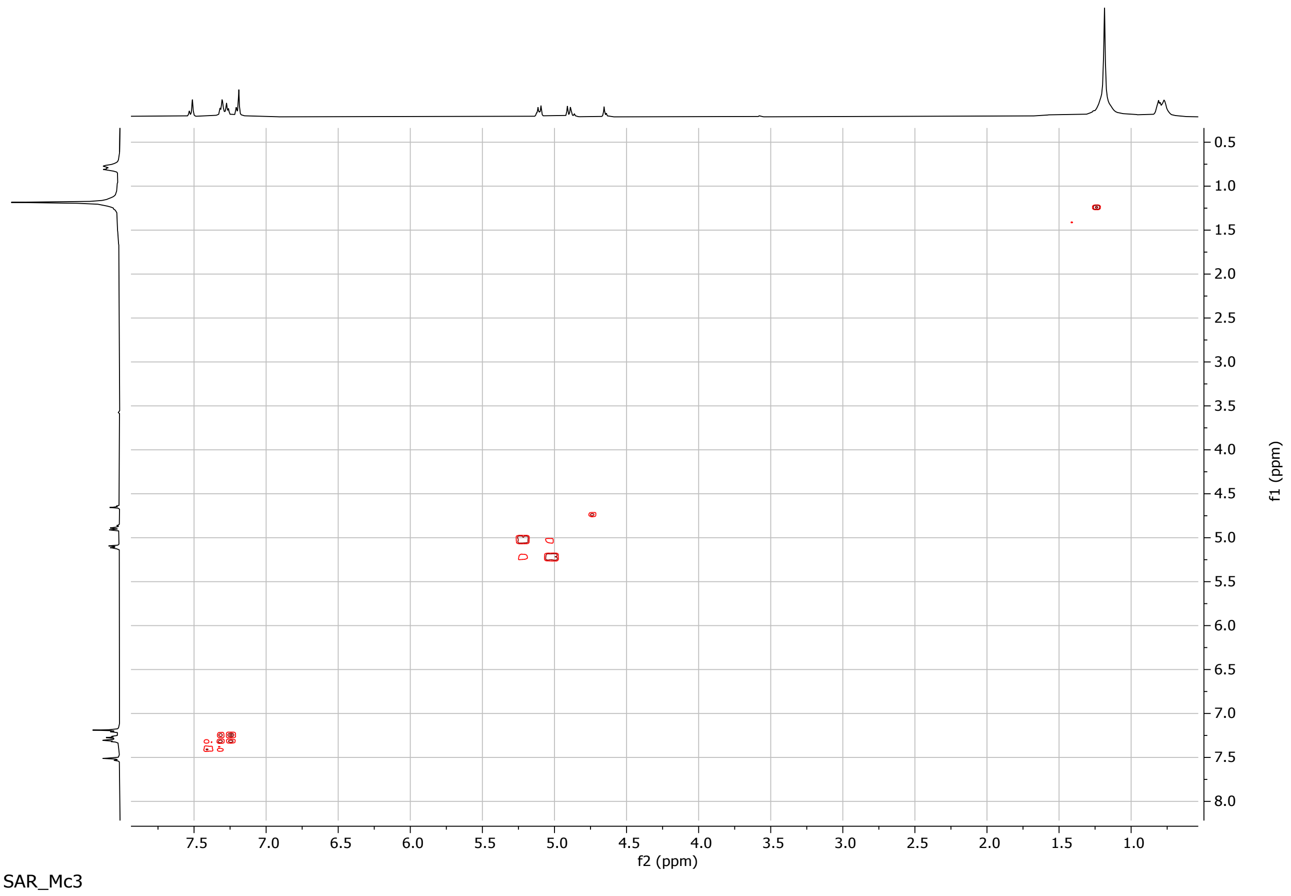

Figure S15. ^{13}C-NMR spectrum of **macrocycle 3**

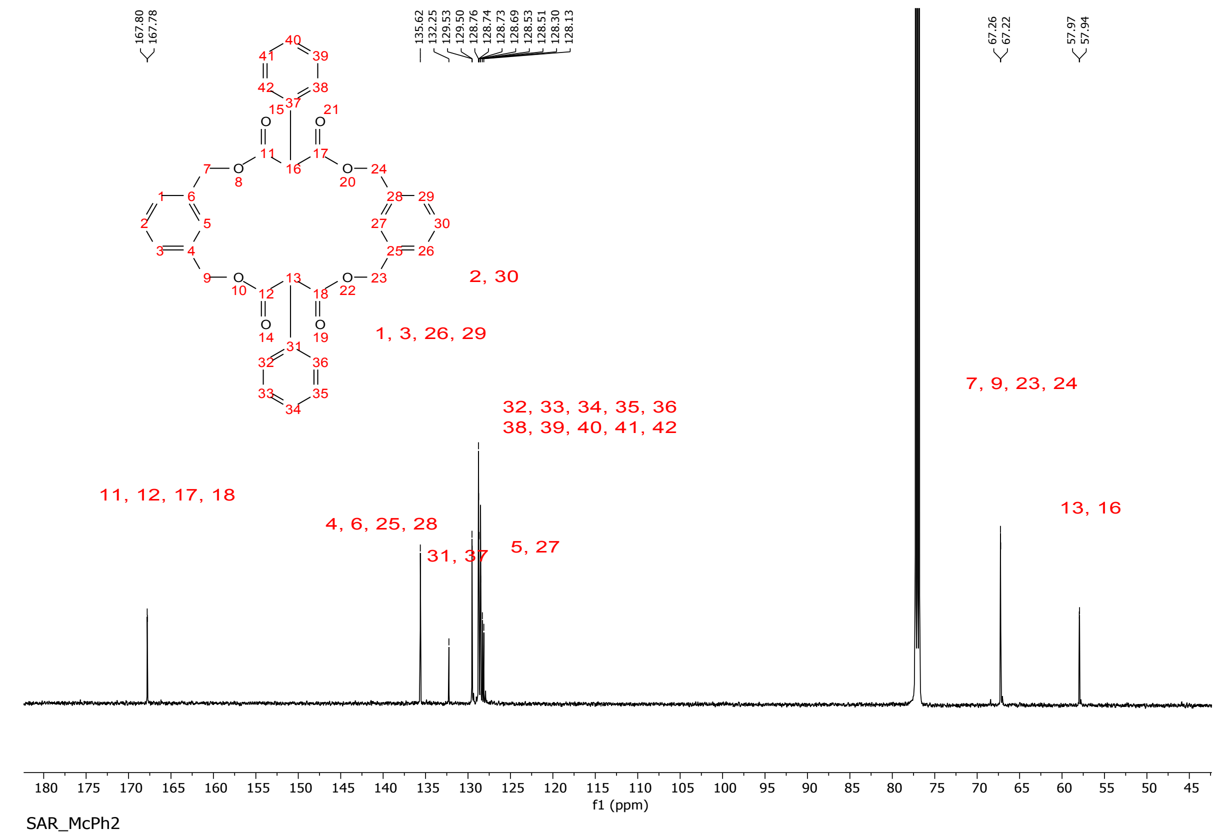

Figure S16. HSQC-NMR spectrum of **macrocycle 3**

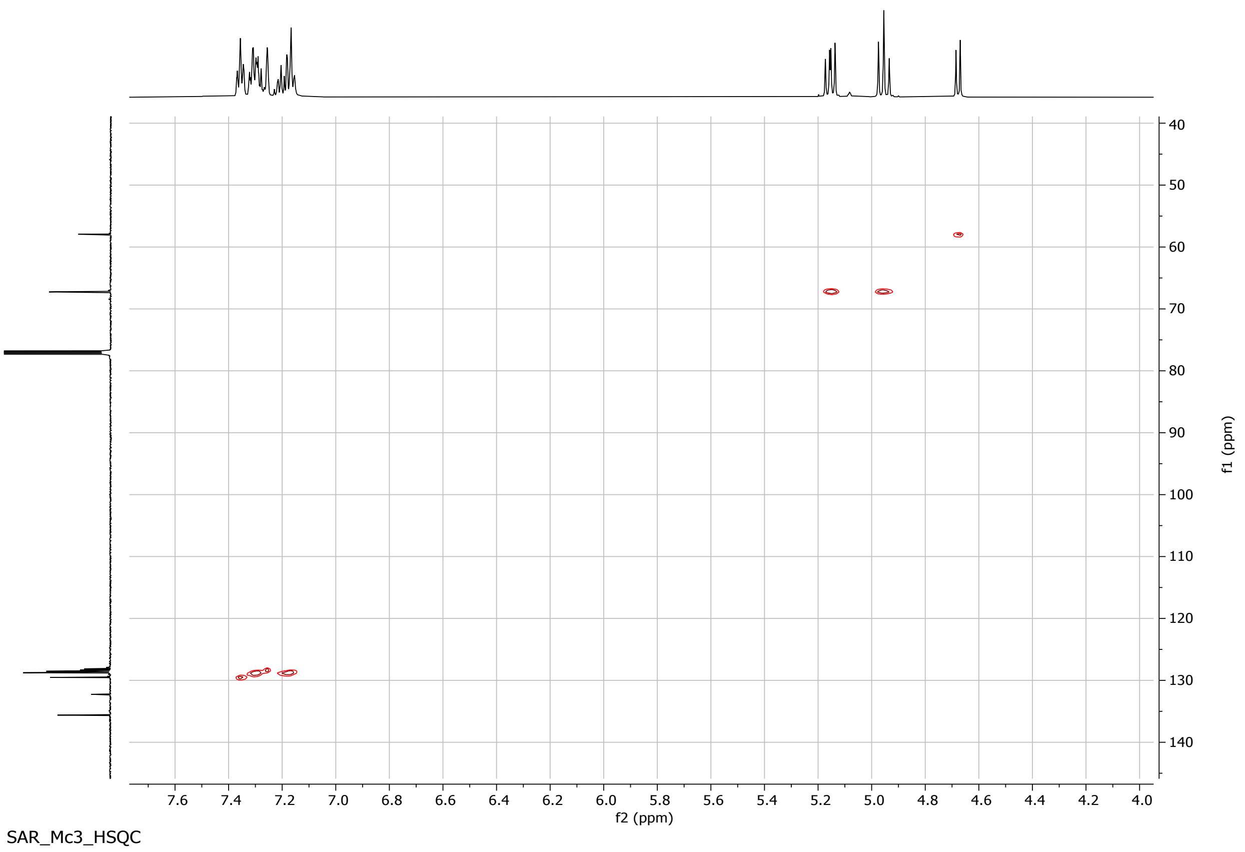

Figure S17. IR spectrum of **macrocycle 3**

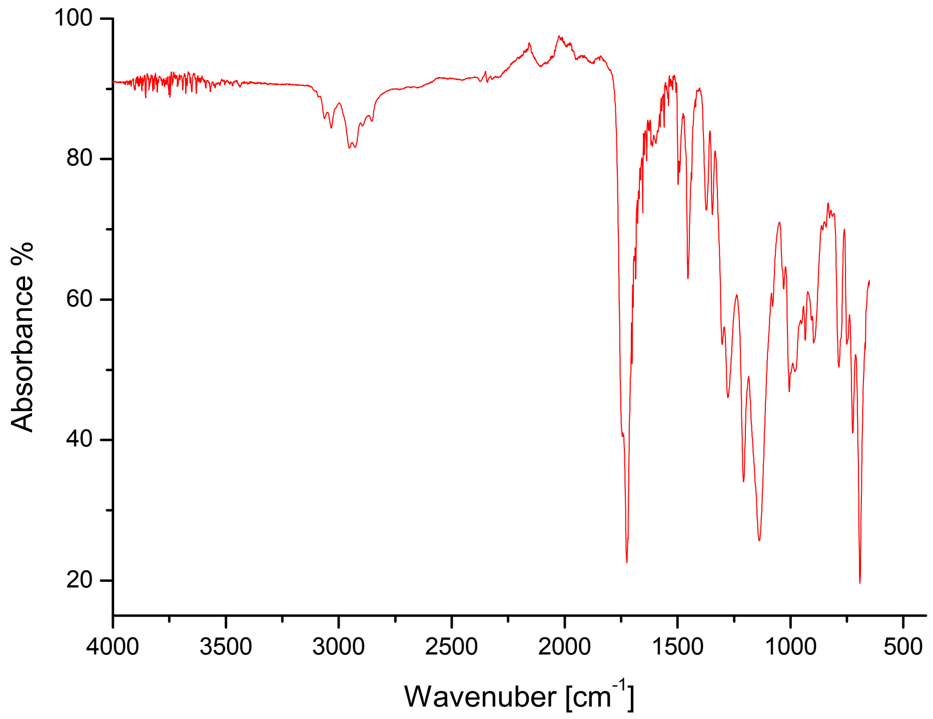

Figure S18. HPLC-MS analysis of **macrocycle 3**

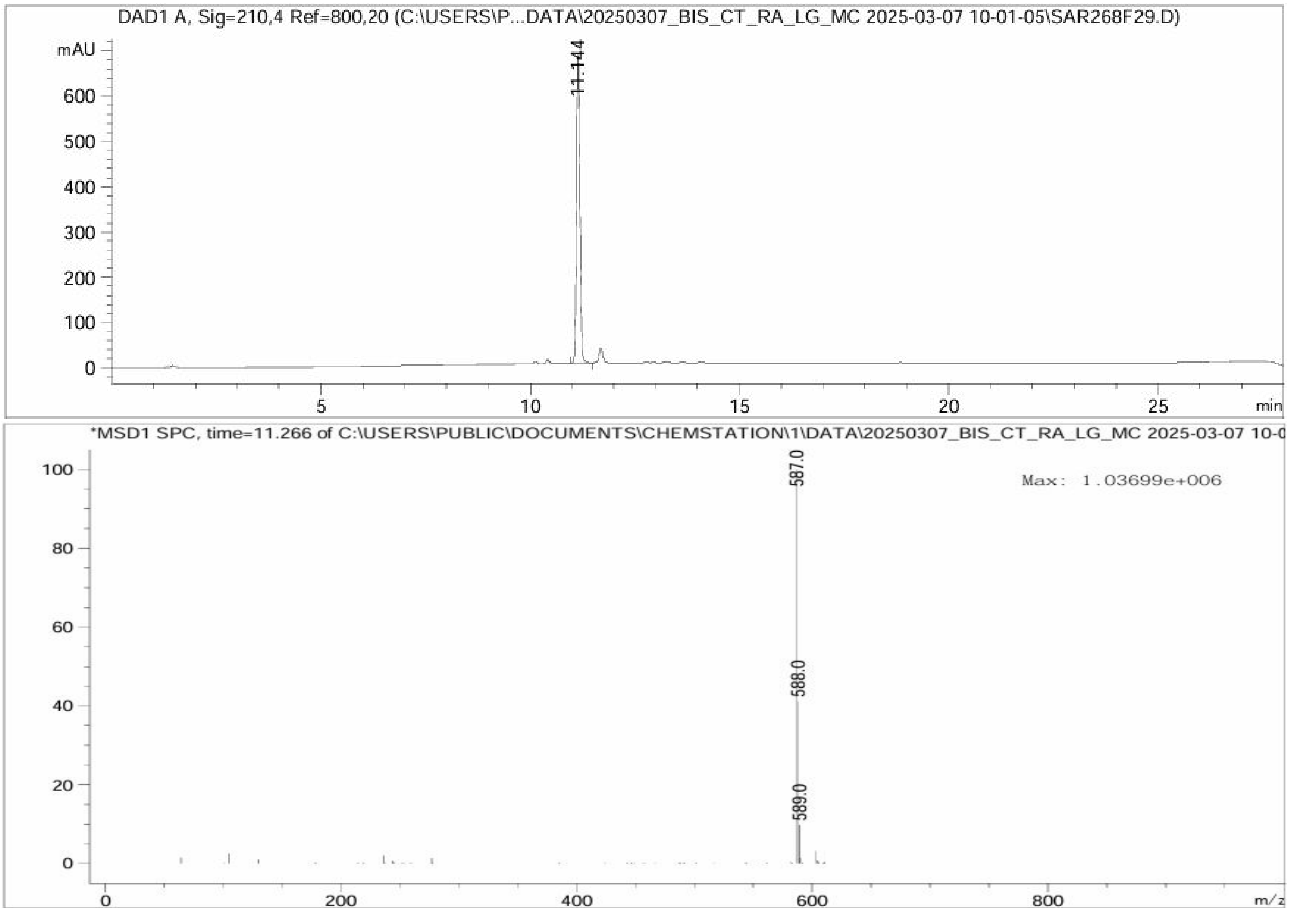

Figure S19. ^{1}H-NMR spectrum of **macrocycle 4**

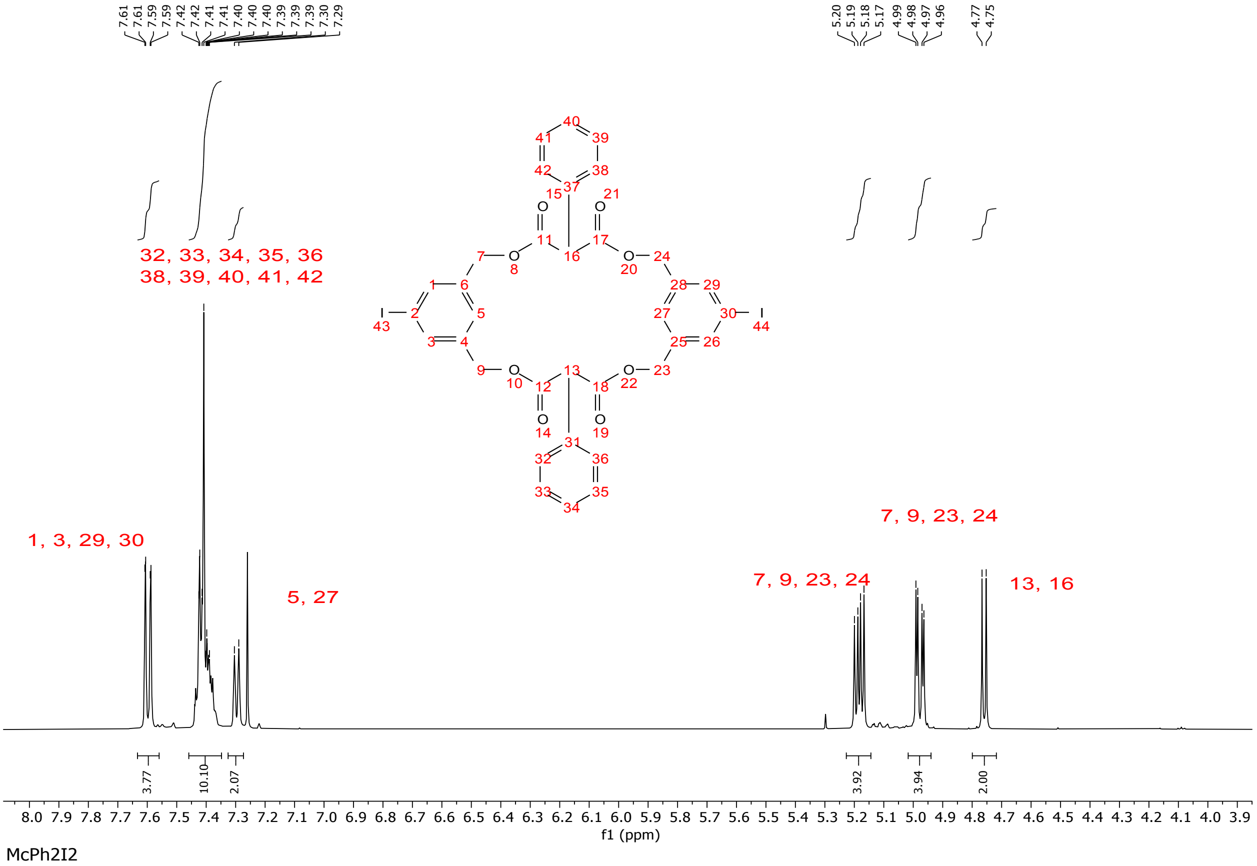

Figure S20. COSY-NMR spectrum of **macrocycle 4**

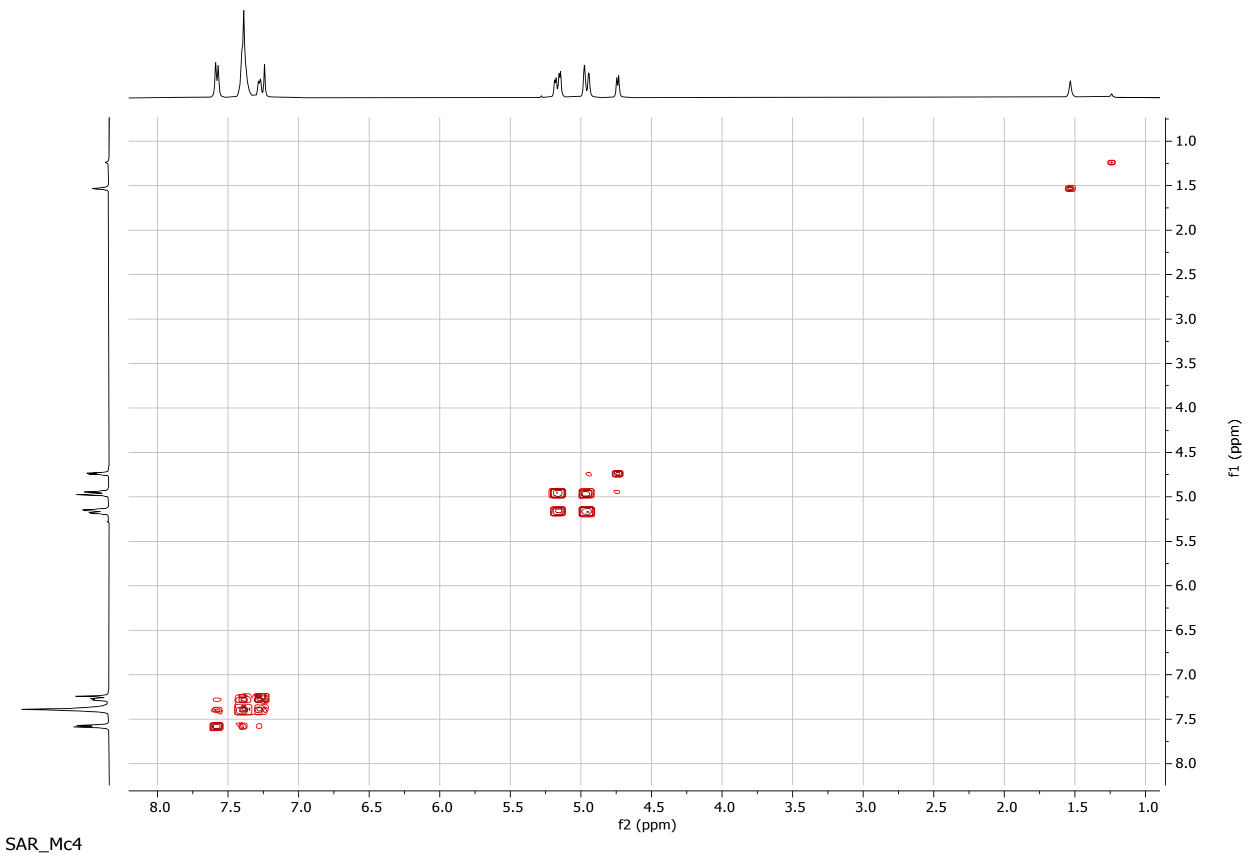

Figure S21. ^{13}C-NMR spectrum of **macrocycle 4**

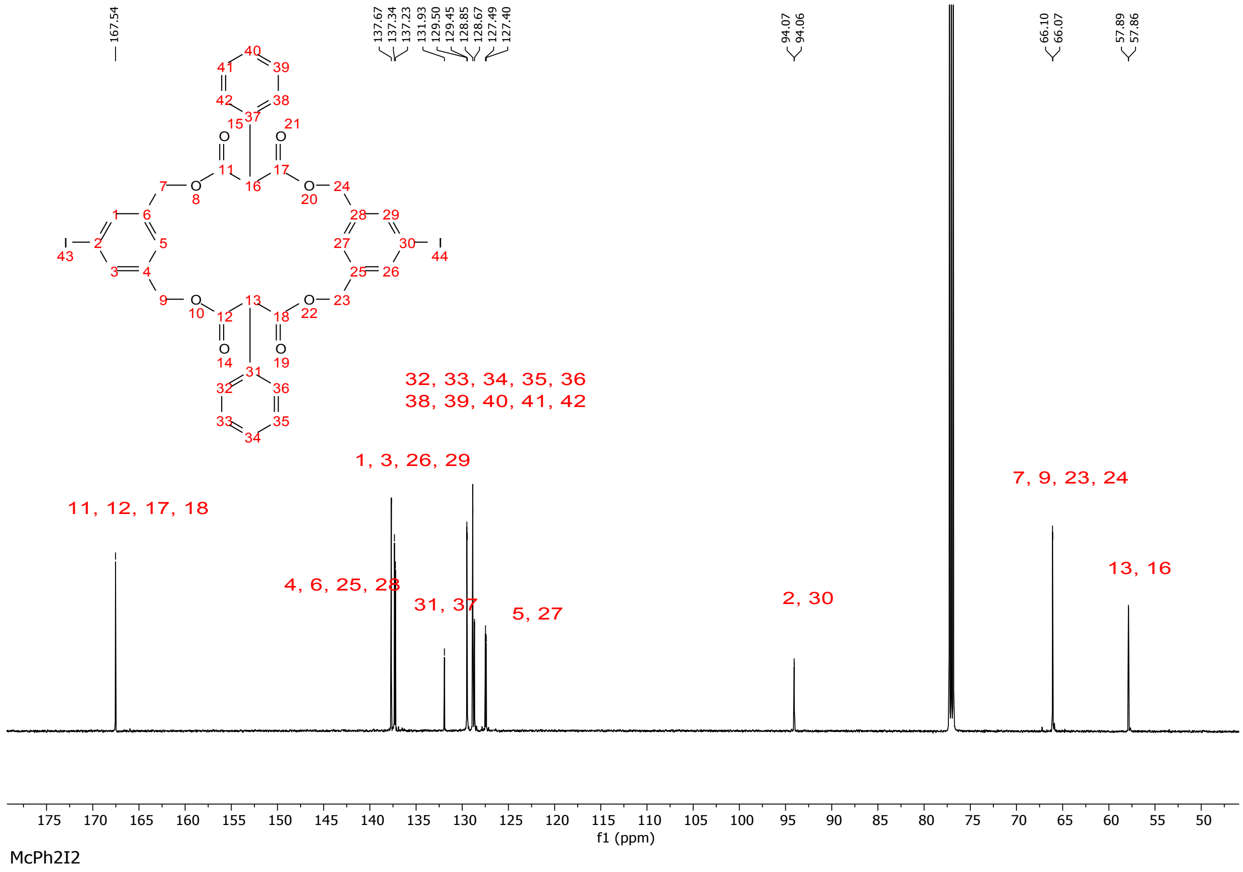

Figure S22. HSQC-NMR spectrum of **macrocycle 4**

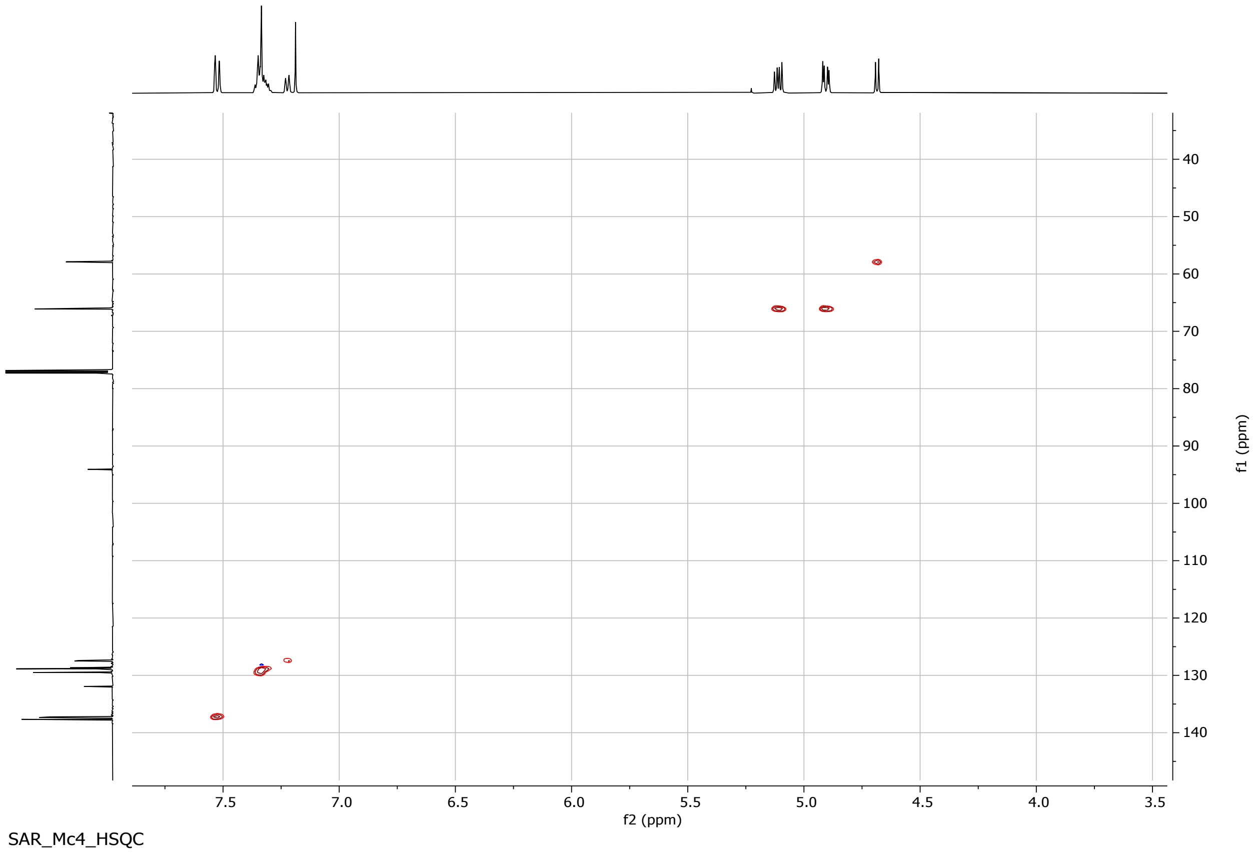

Figure S23. IR spectrum of **macrocycle 4**

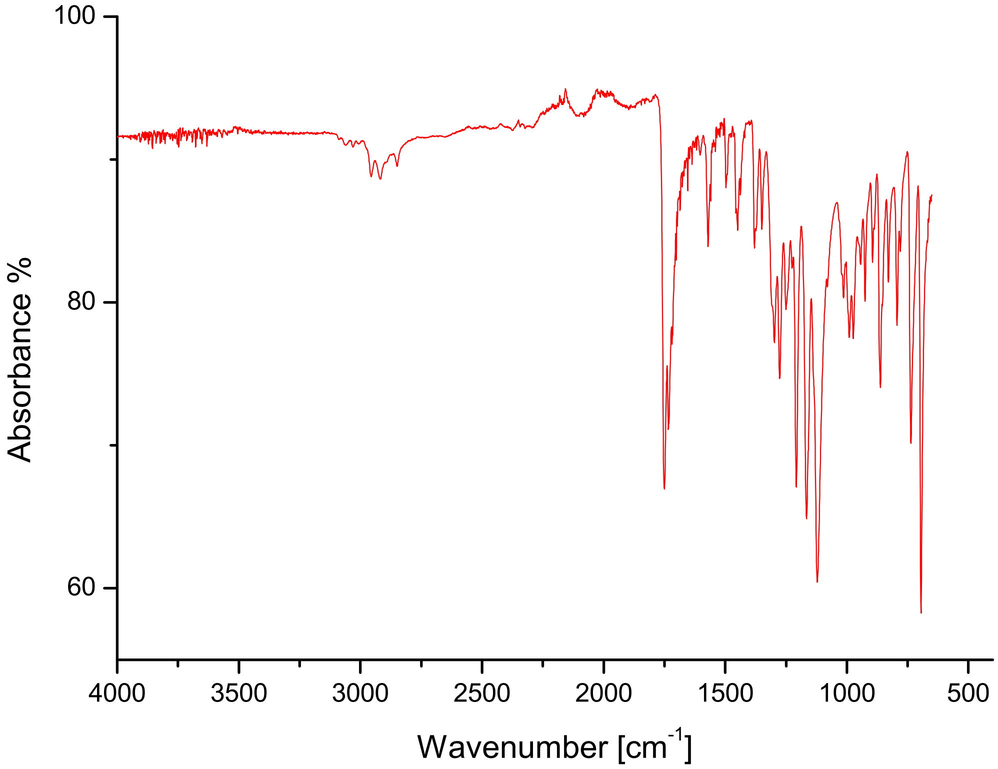

Figure S24. HPLC-MS analysis of **macrocycle 4**

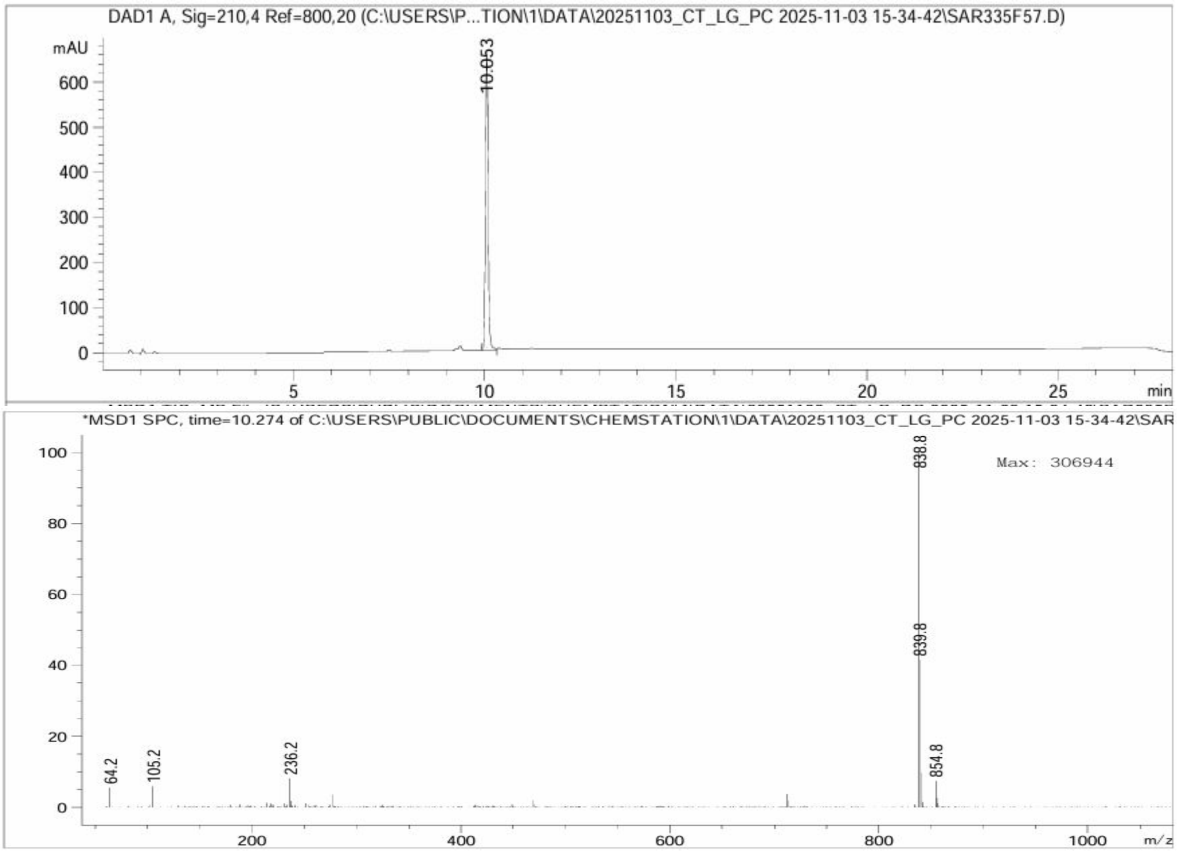

Figure S25. ^{1}H-NMR spectrum of **macrocycle 5**

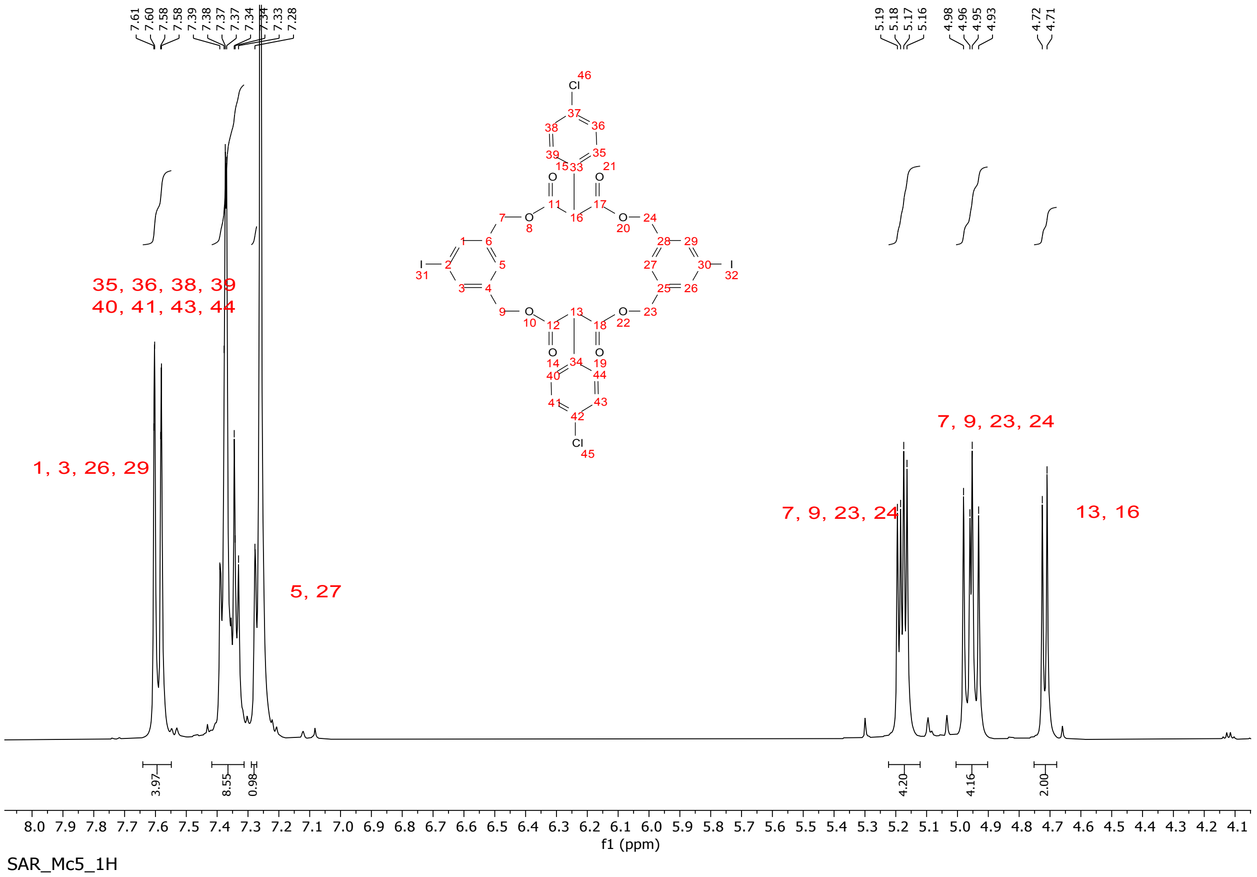

Figure S26. COSY-NMR spectrum of **macrocycle 5**

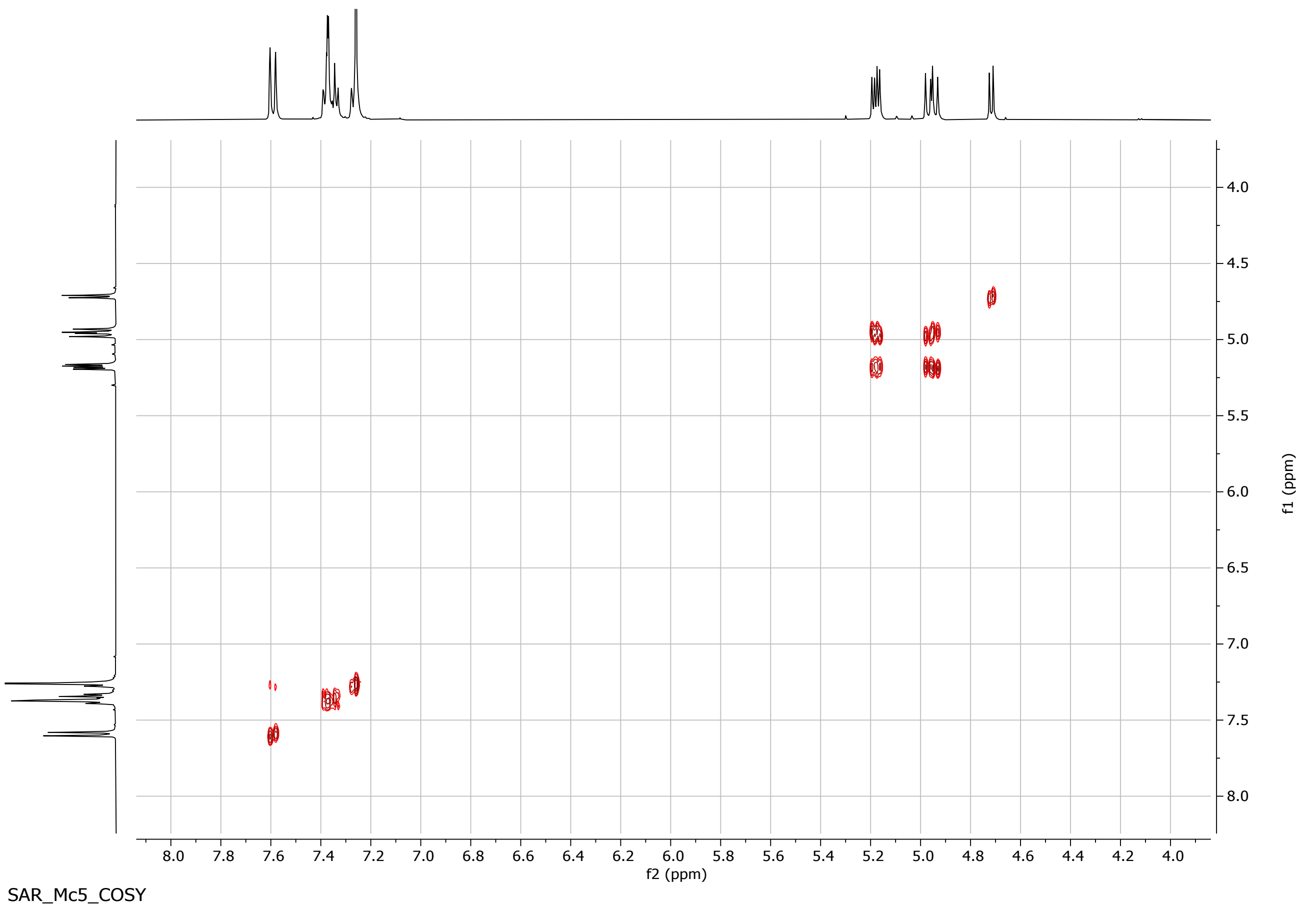

Figure S27. ^{13}C-NMR spectrum of **macrocycle 5**

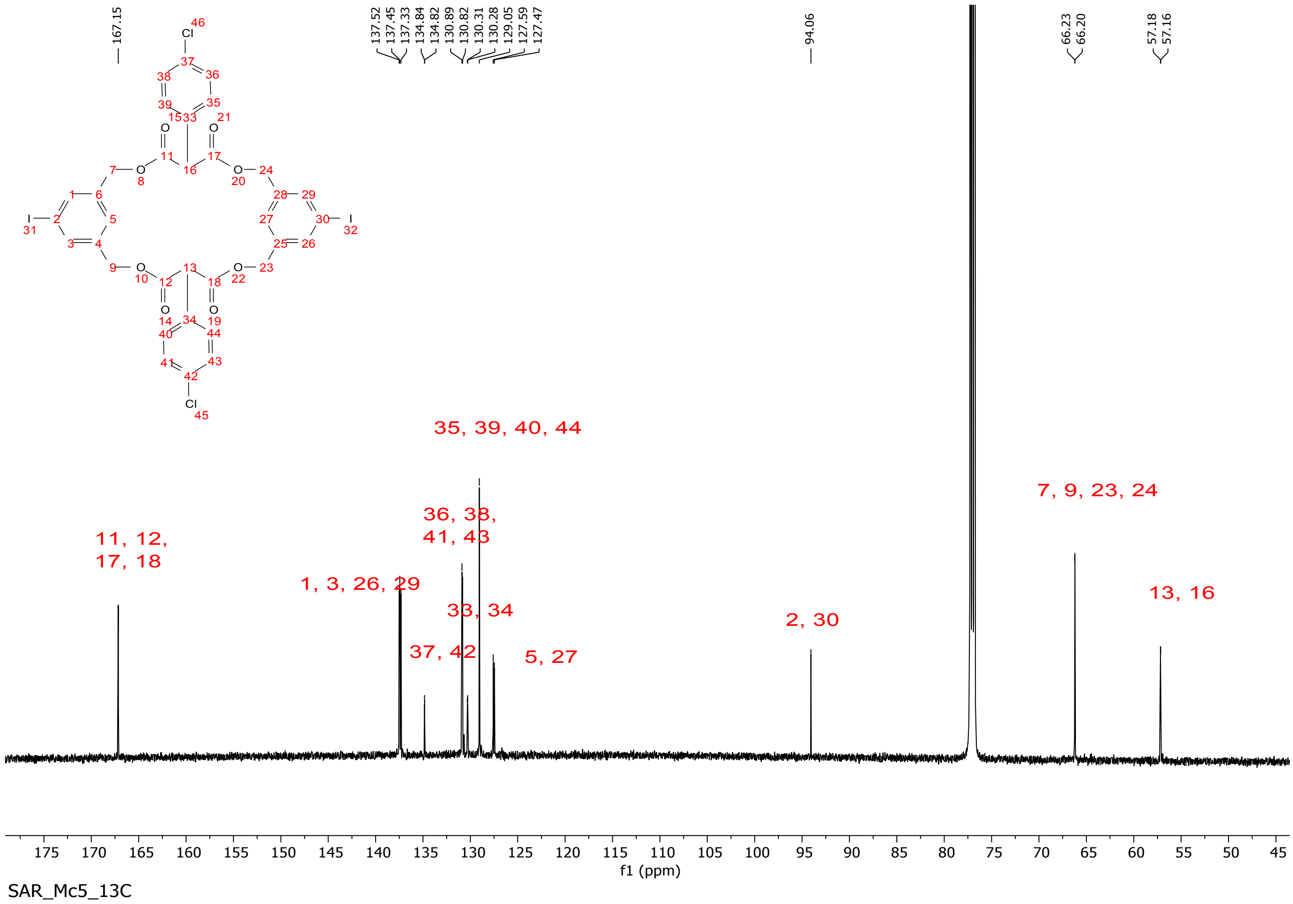

Figure S28. HSQC-NMR spectrum of **macrocycle 5**

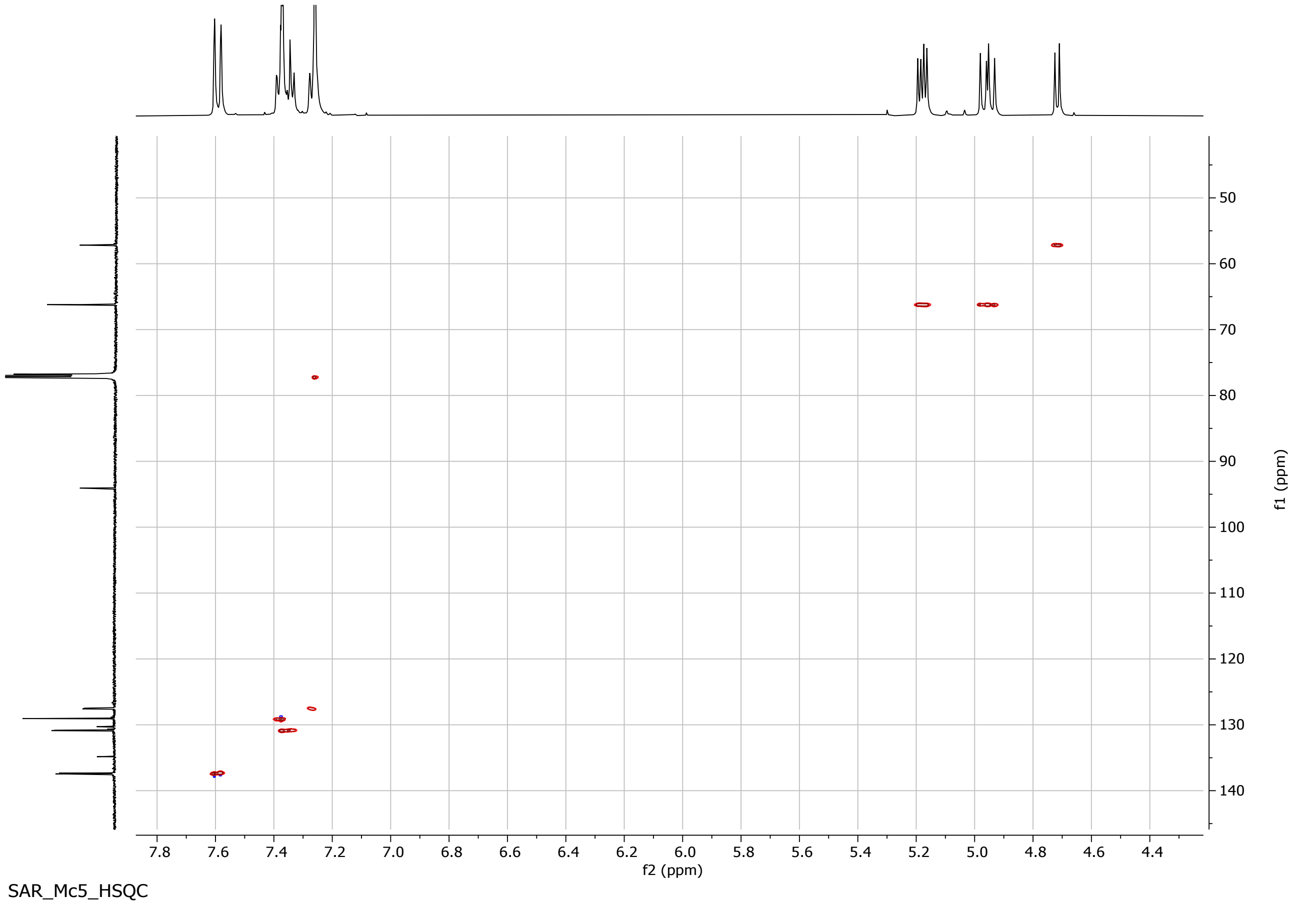

Figure S29. IR spectrum of **macrocycle 5**

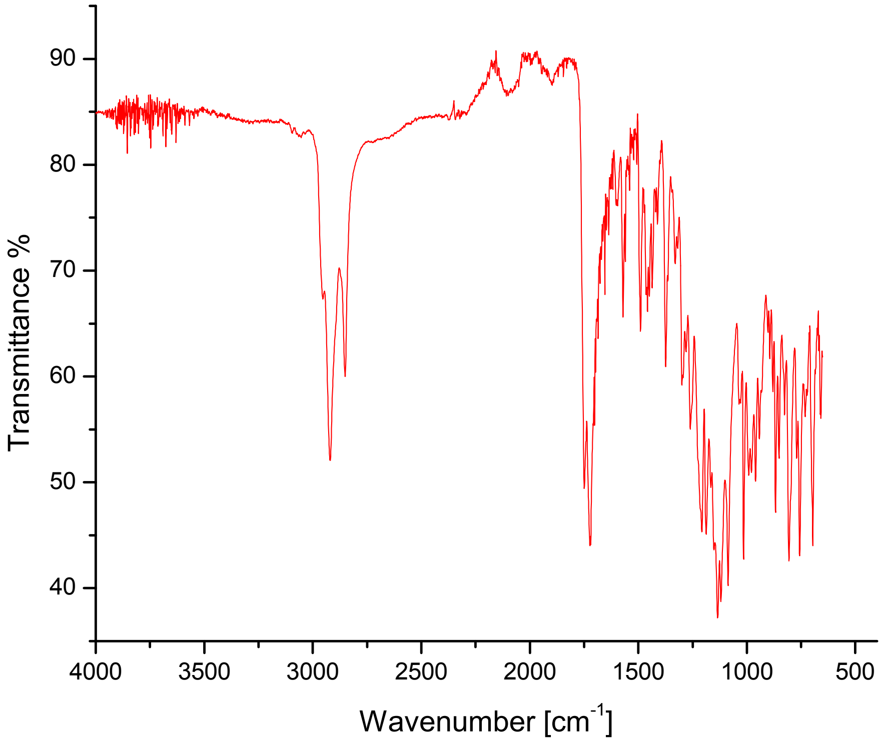

Figure S30. HPLC-MS analysis of **macrocycle 5**

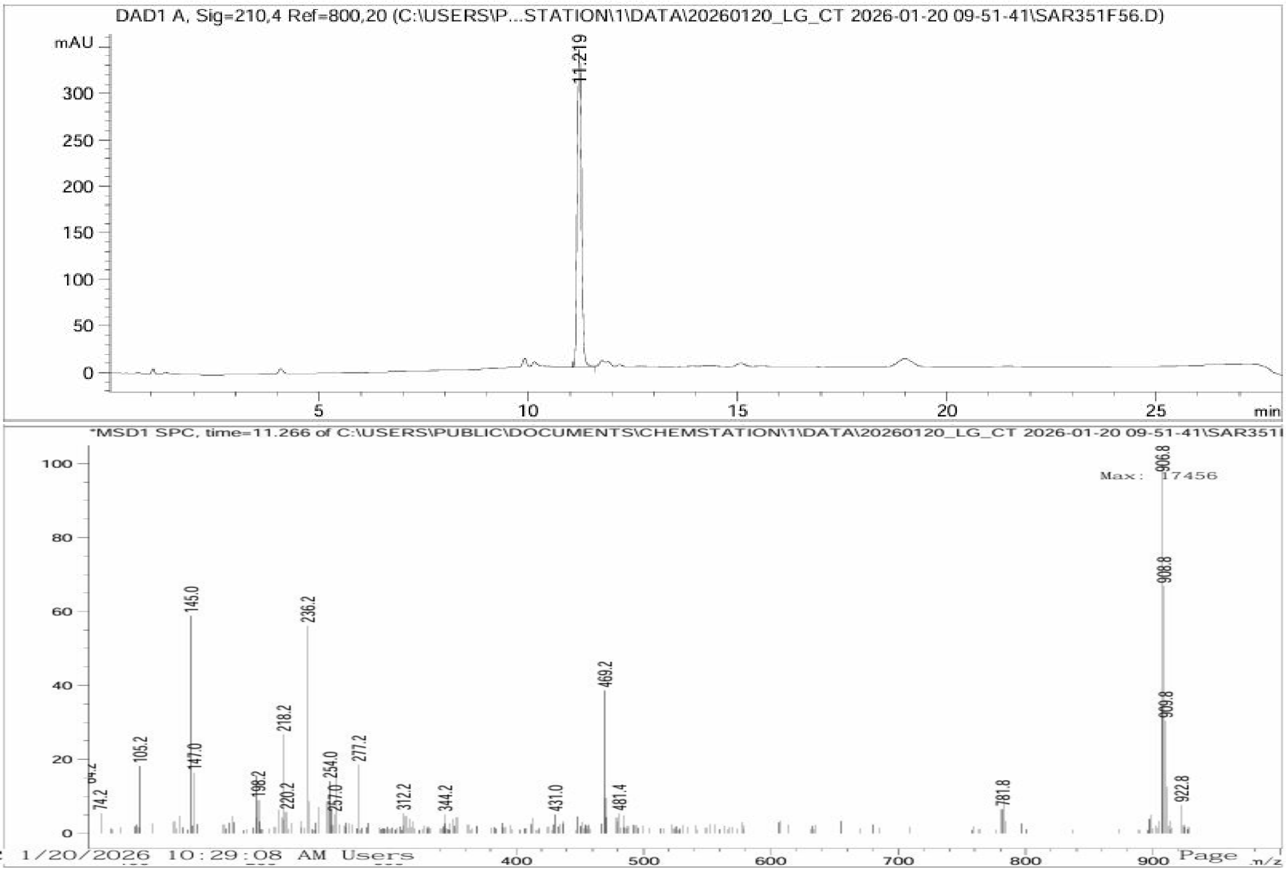

Figure S31. ^{1}H-NMR spectrum of **macrocycle 6**

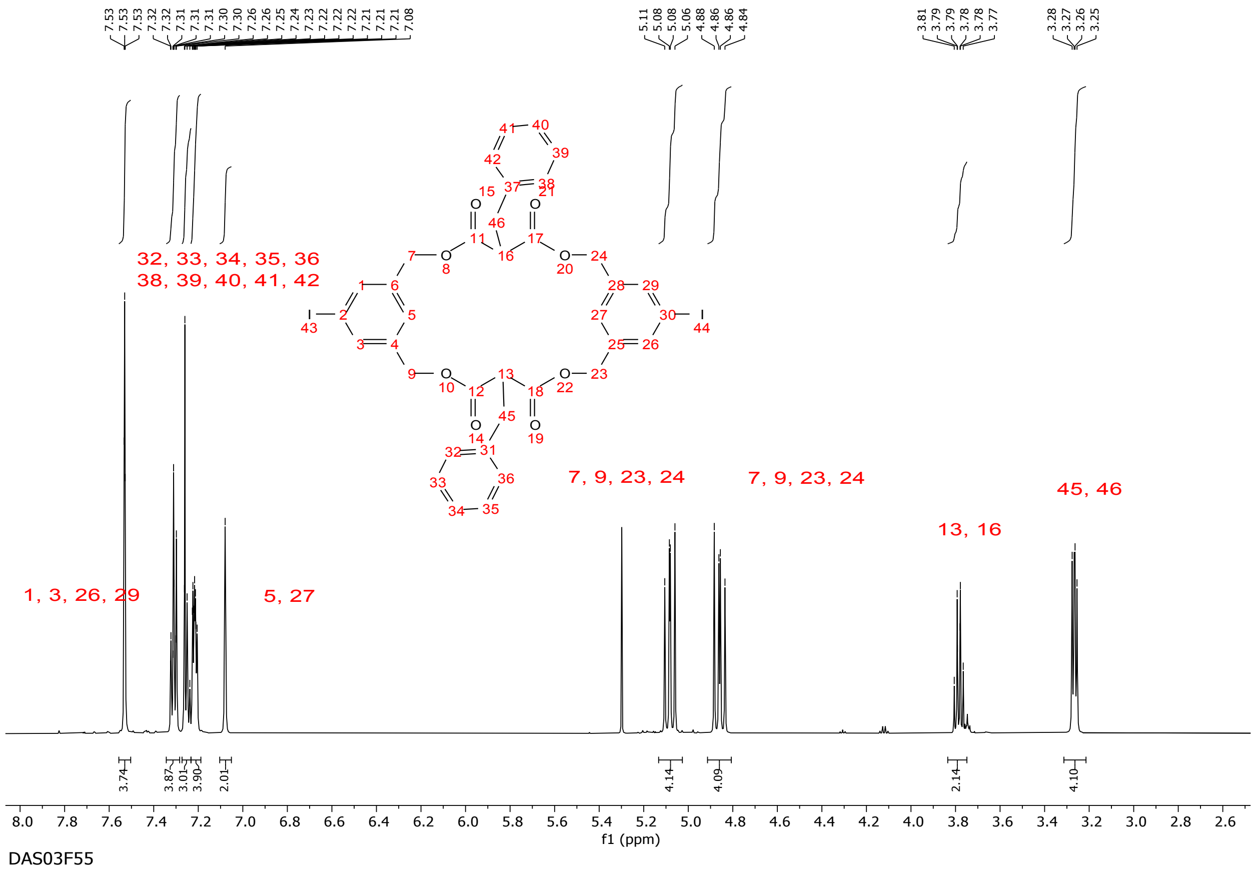

Figure S32. COSY-NMR spectrum of **macrocycle 6**

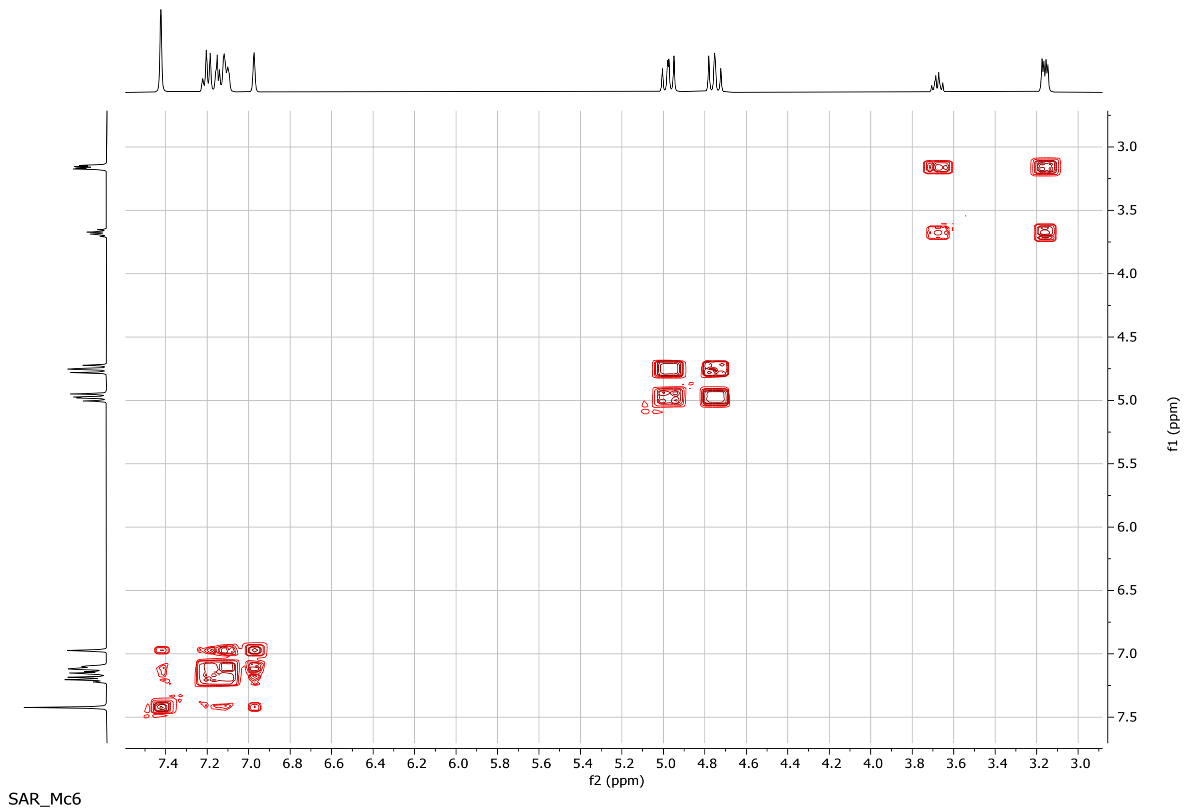

Figure S33. ^{13}C-NMR spectrum of **macrocycle 6**

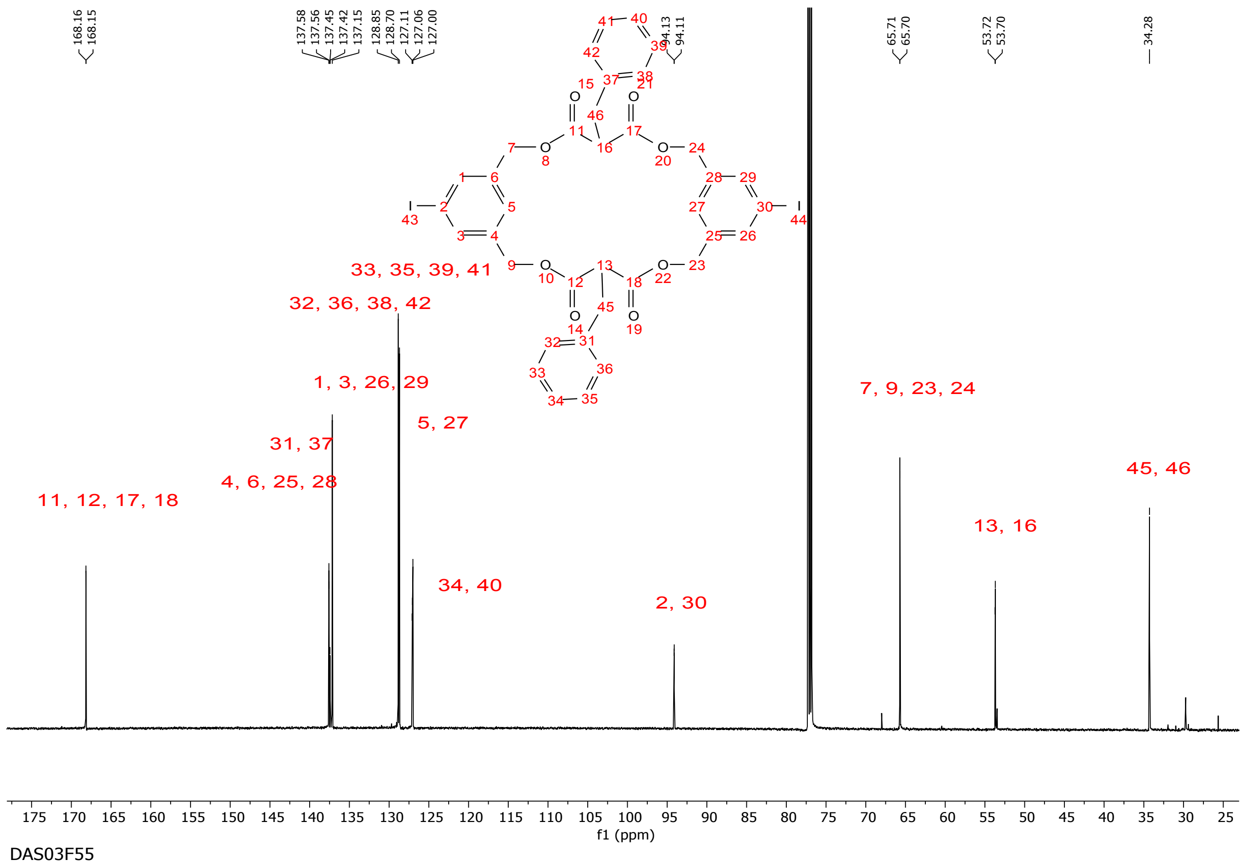

Figure S34. HSQC-NMR spectrum of **macrocycle 6**

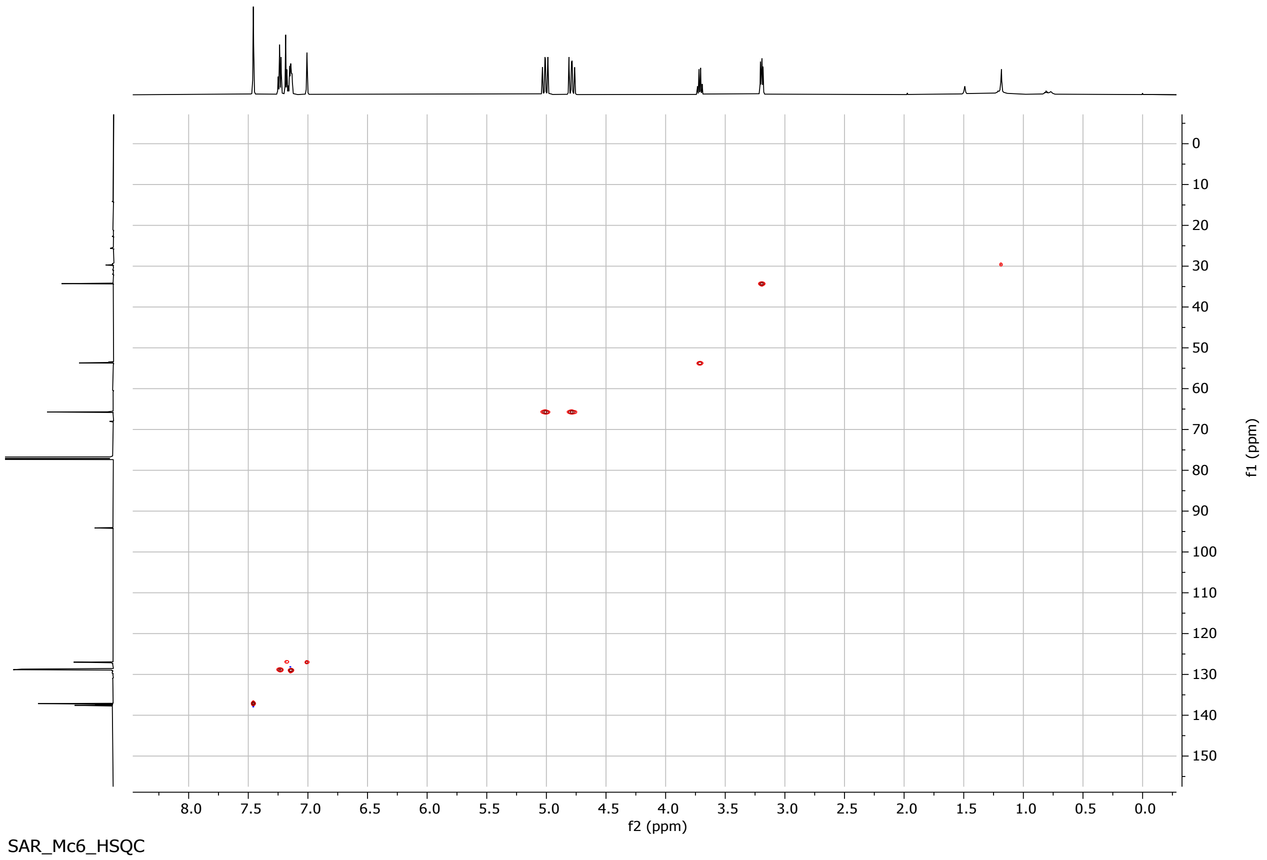

Figure S35. IR spectrum of **macrocycle 6**

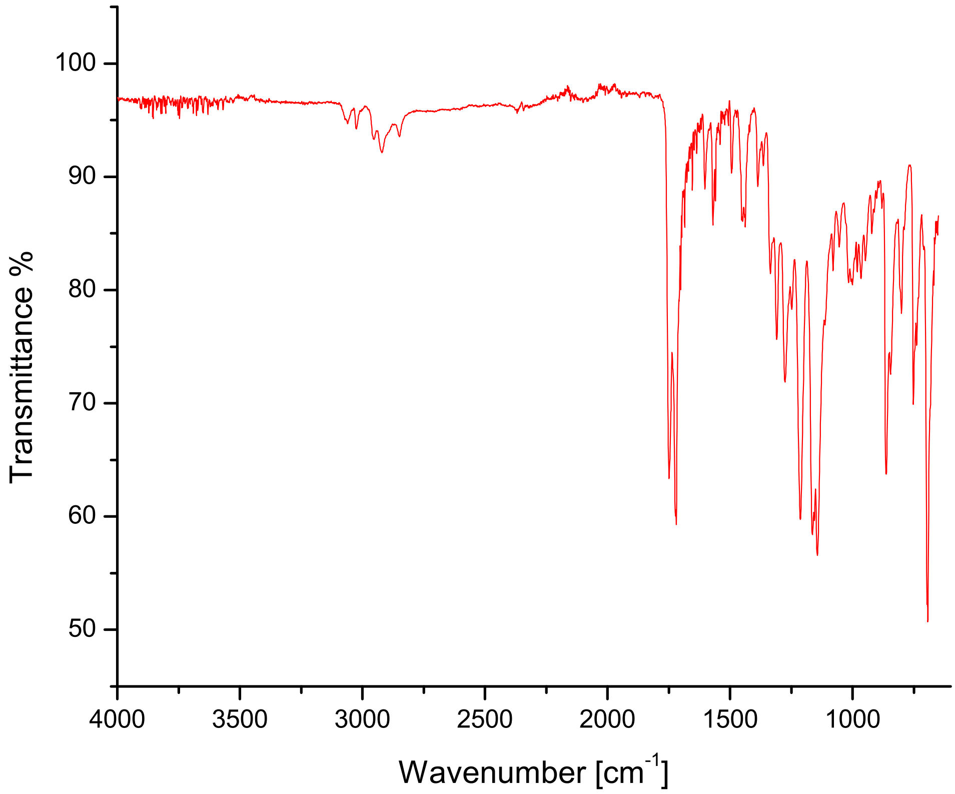

Figure S36. HPLC-MS analysis of **macrocycle 6**

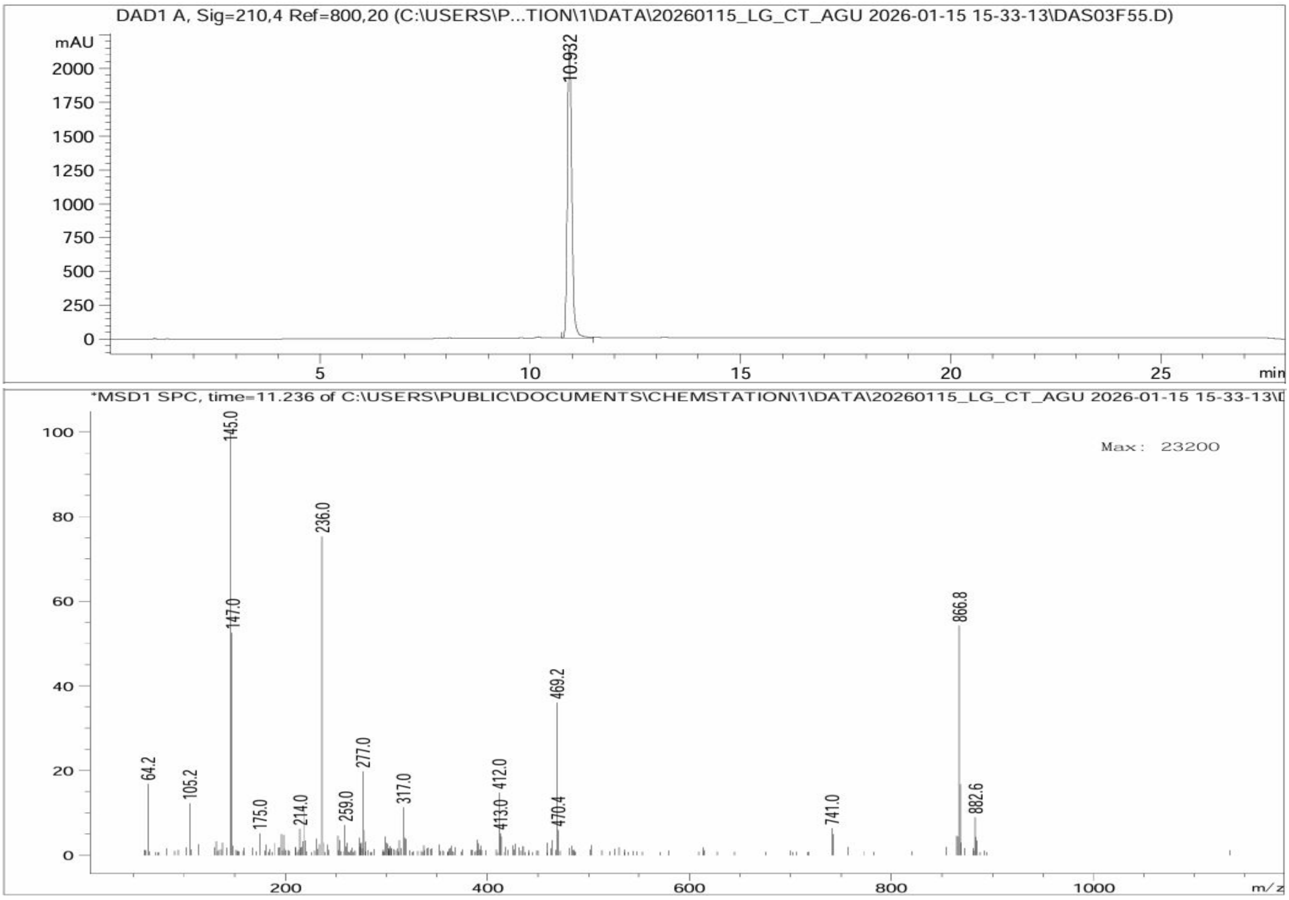

Figure S37. ^{1}H-NMR spectrum of **macrocycle** $(\mathbf{Me_3Si})_2$**-7**

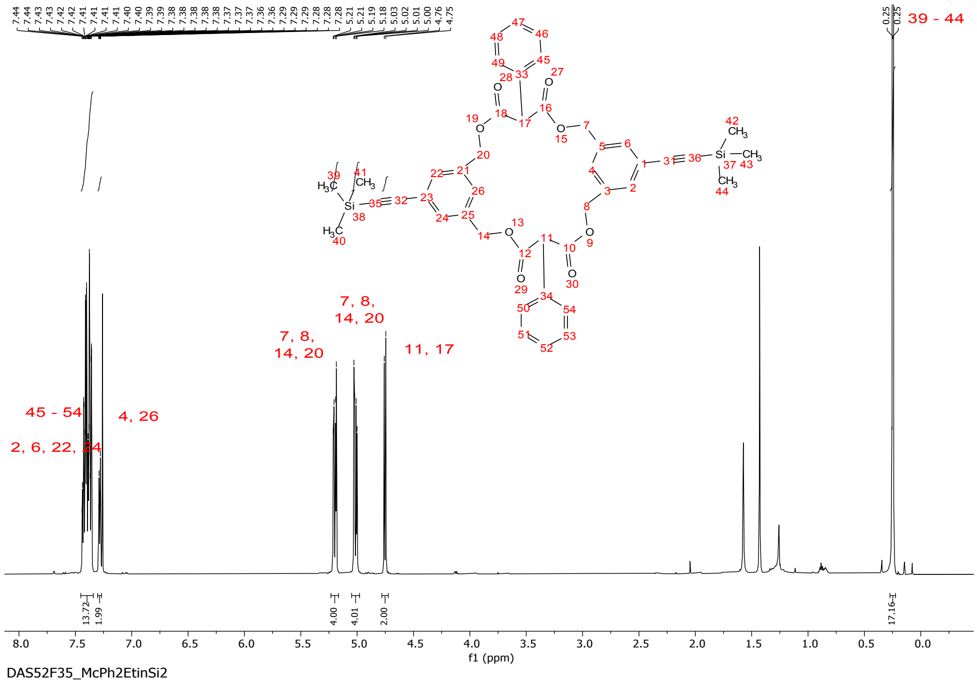

Figure S38. ^{13}C-NMR spectrum of **macrocycle** $(\mathbf{Me_3Si})_2$**-7**

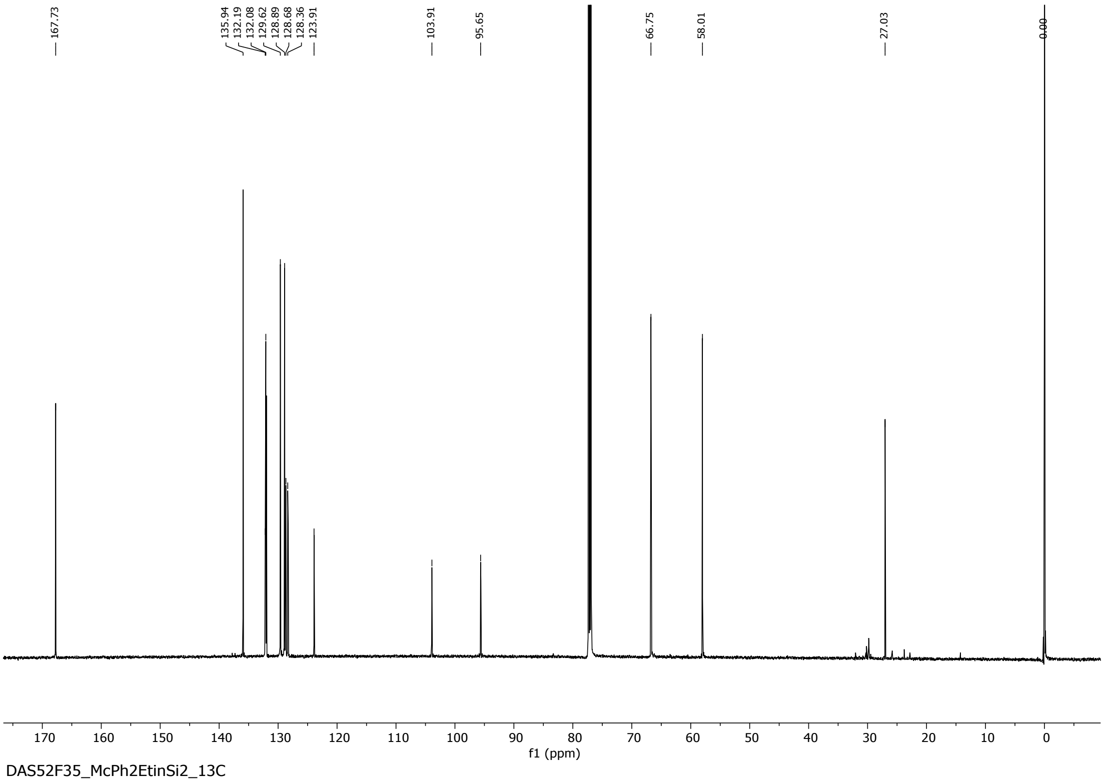

Figure S39. IR spectrum of **macrocycle** **($(Me_3Si)_2$-7**

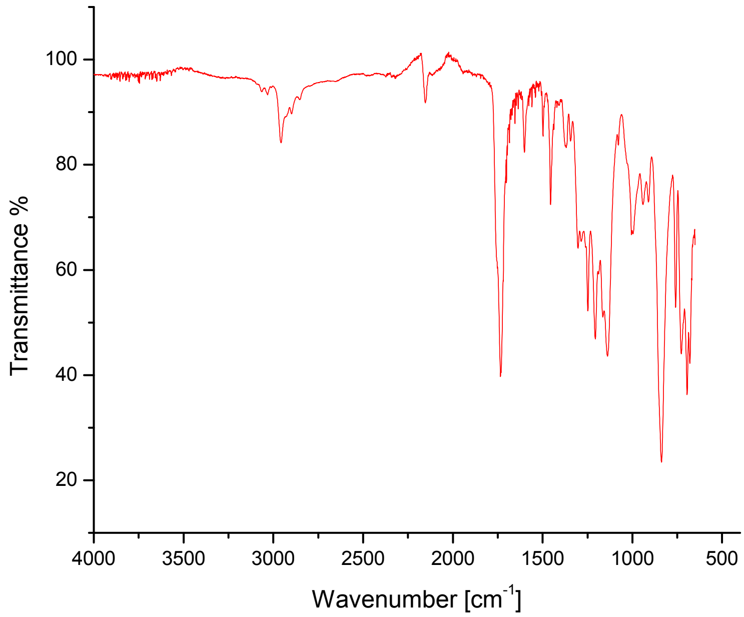

Figure S40. ^{1}H-NMR spectrum of **macrocycle 7**

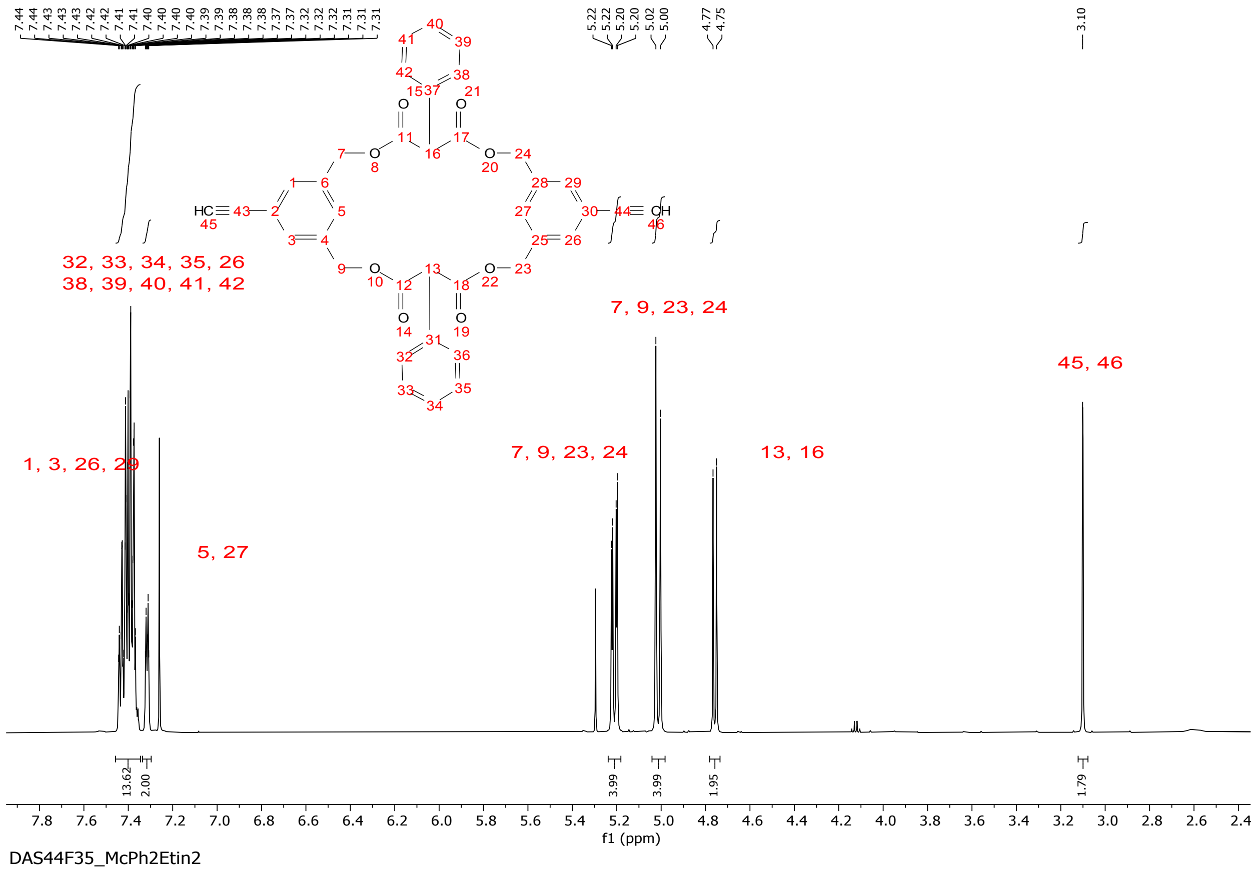

Figure S41. COSY-NMR spectrum of **macrocycle 7**

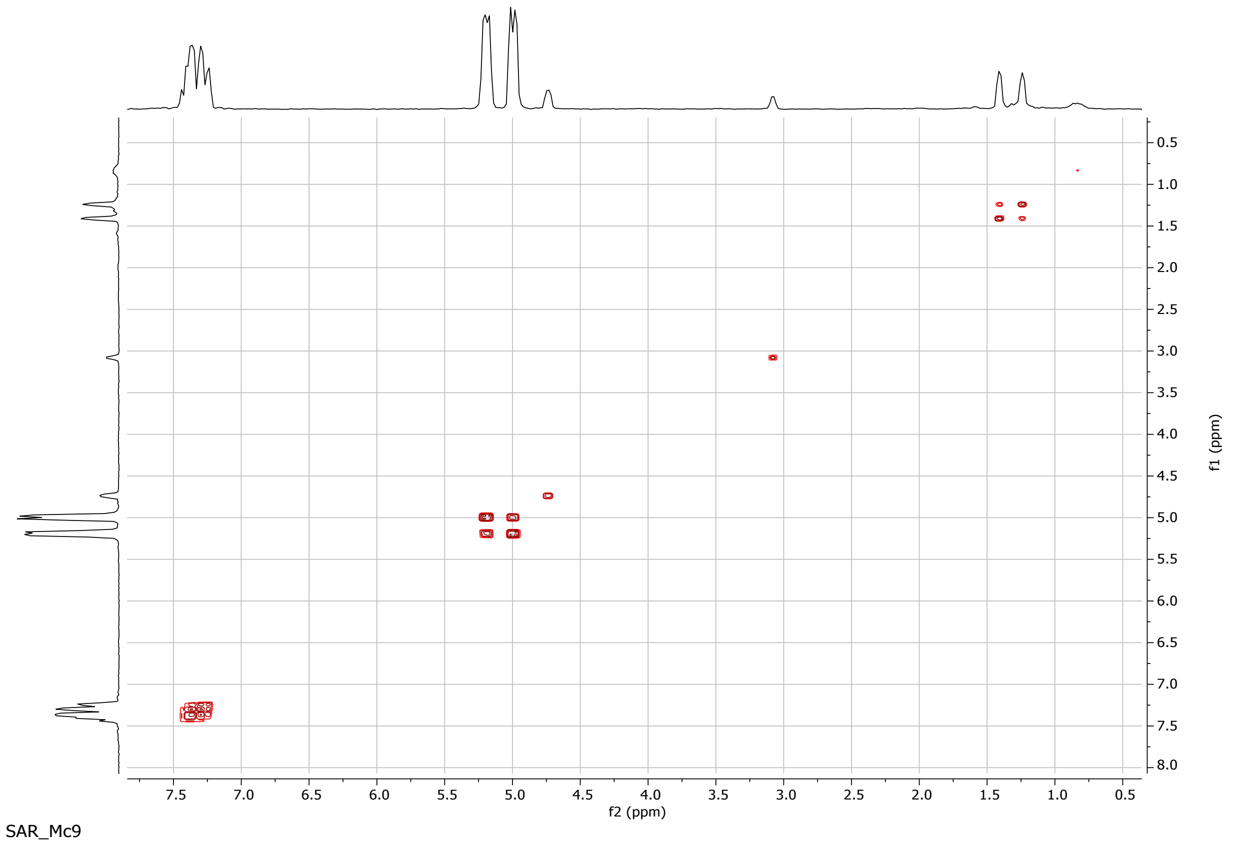

Figure S42. ^{13}C-NMR spectrum of **macrocycle 7**

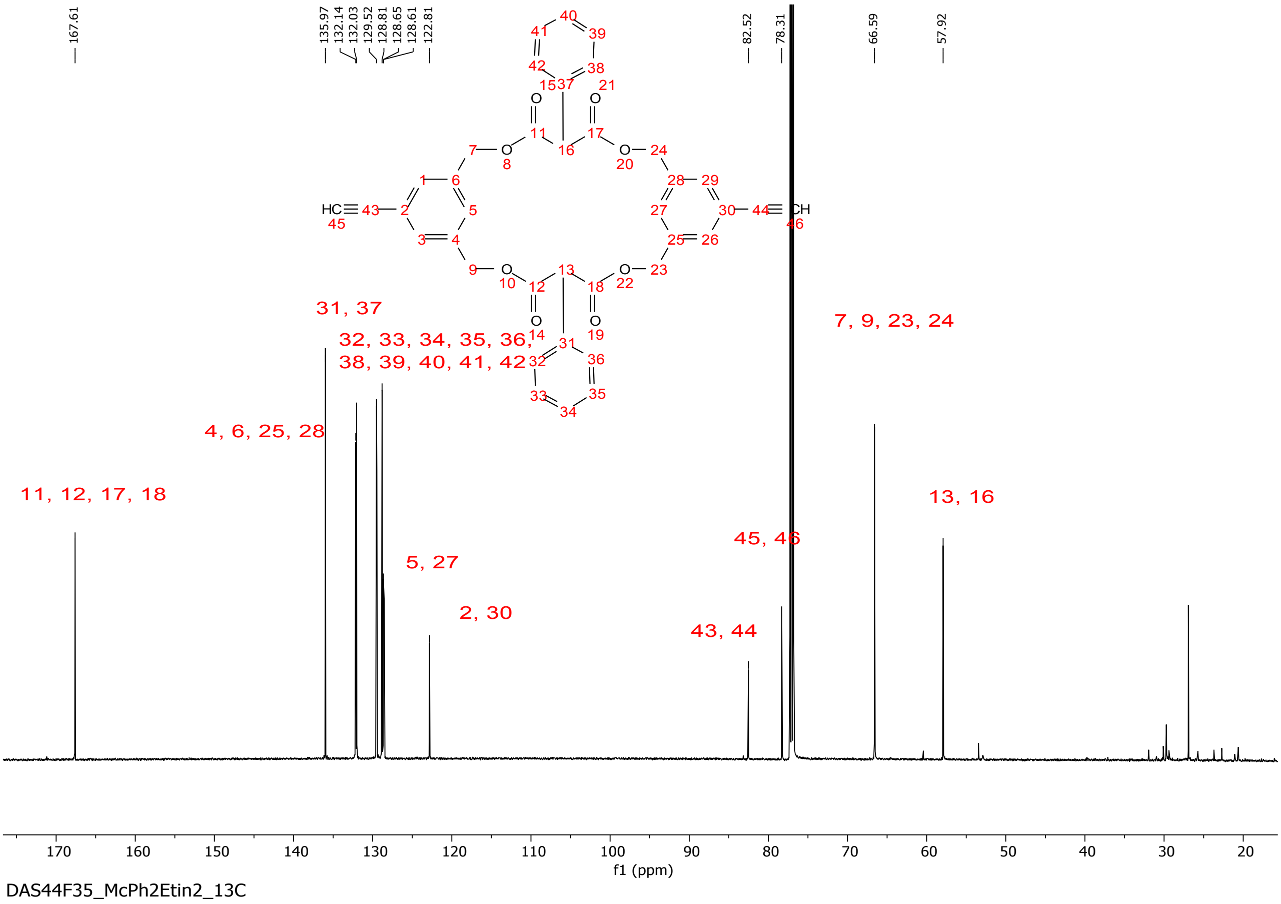

Figure S43. HSQC-NMR spectrum of **macrocycle 7**

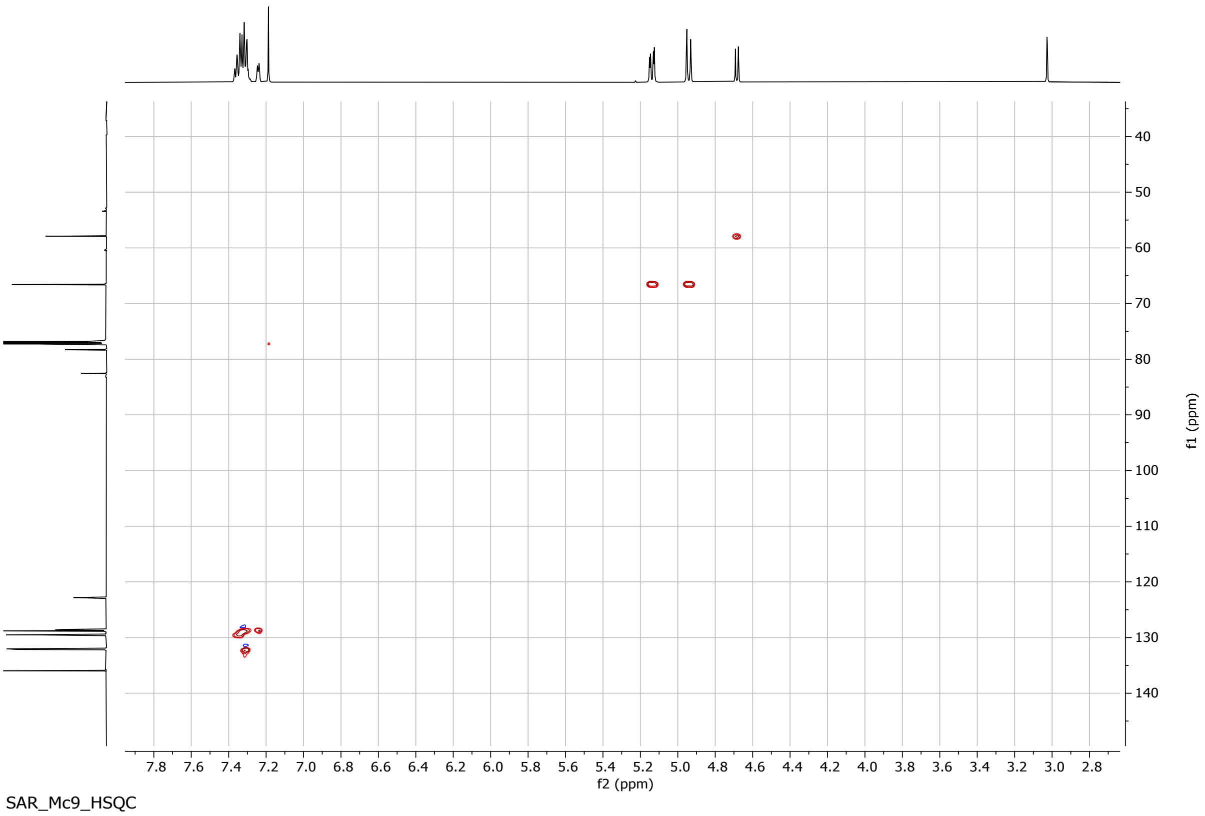

Figure S44. IR spectrum of **macrocycle 7**

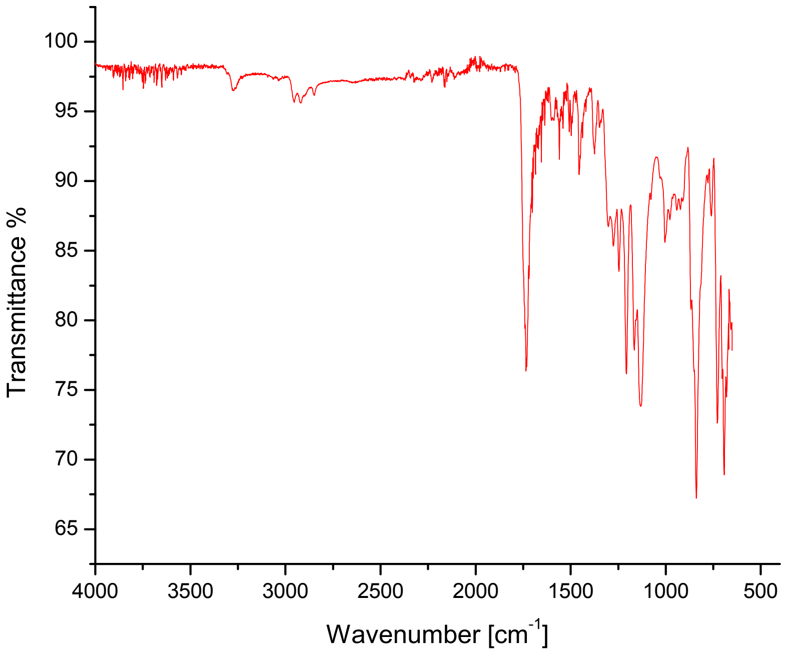

Figure S45. HPLC-MS analysis of **macrocycle 7**

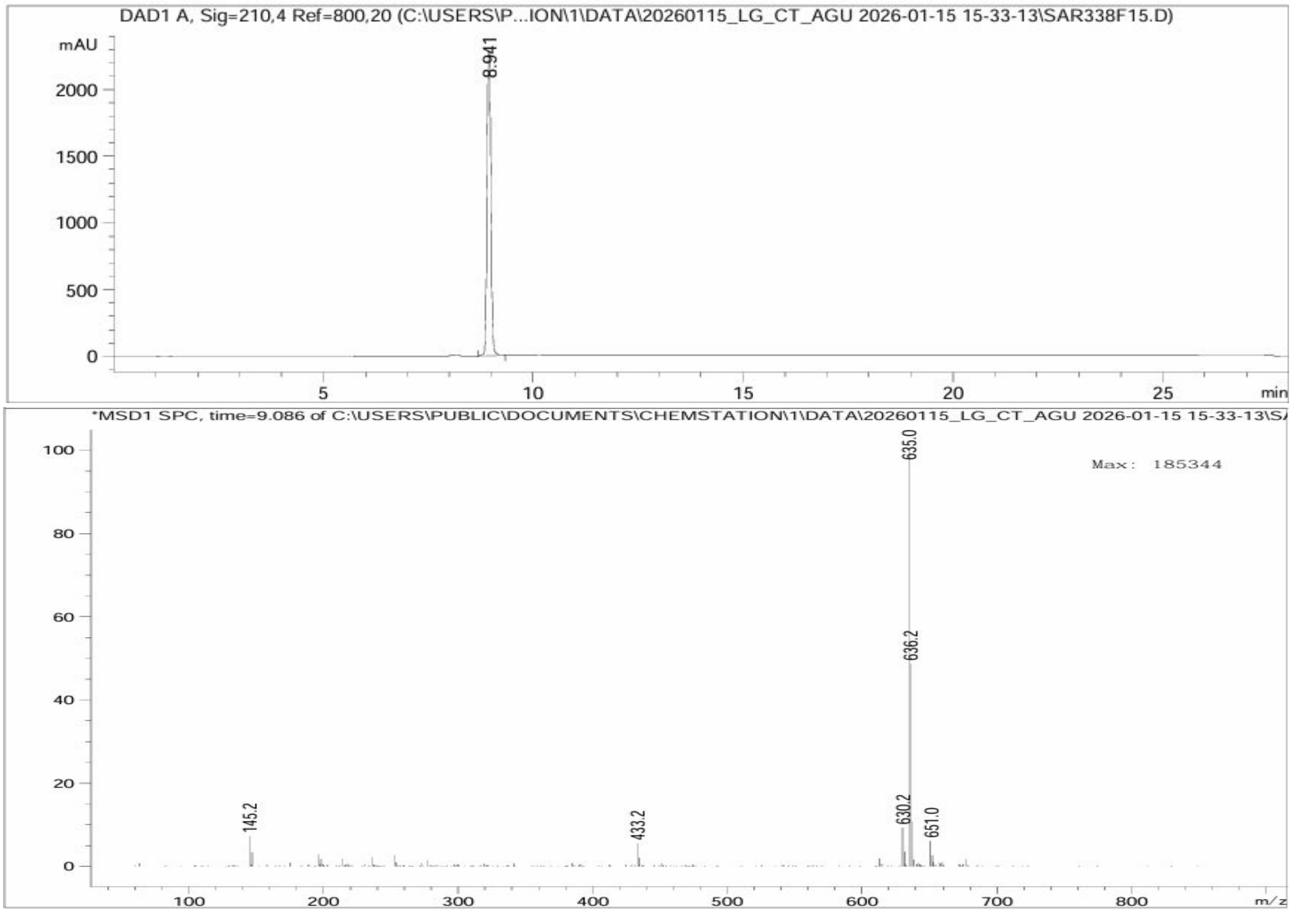

Table S1. HPLC-MS analysis of the crude reaction of macrocyclization for the formation of **1**

Peak Mc1	Macrocycle	Exact mass	Mass found	Ret. Time (min)	Area %
**1**	Dimeric	412.12	435 ($M+Na^+$), 451 ($M+K^+$)	8.009	53.8
**2**	Trimeric	618.17	641 ($M+Na^+$),	9.333	26.1
**3**	Tetrameric	824.23	647 ($M+Na^+$), 864 ($M+K^+$)	10.183	20.1

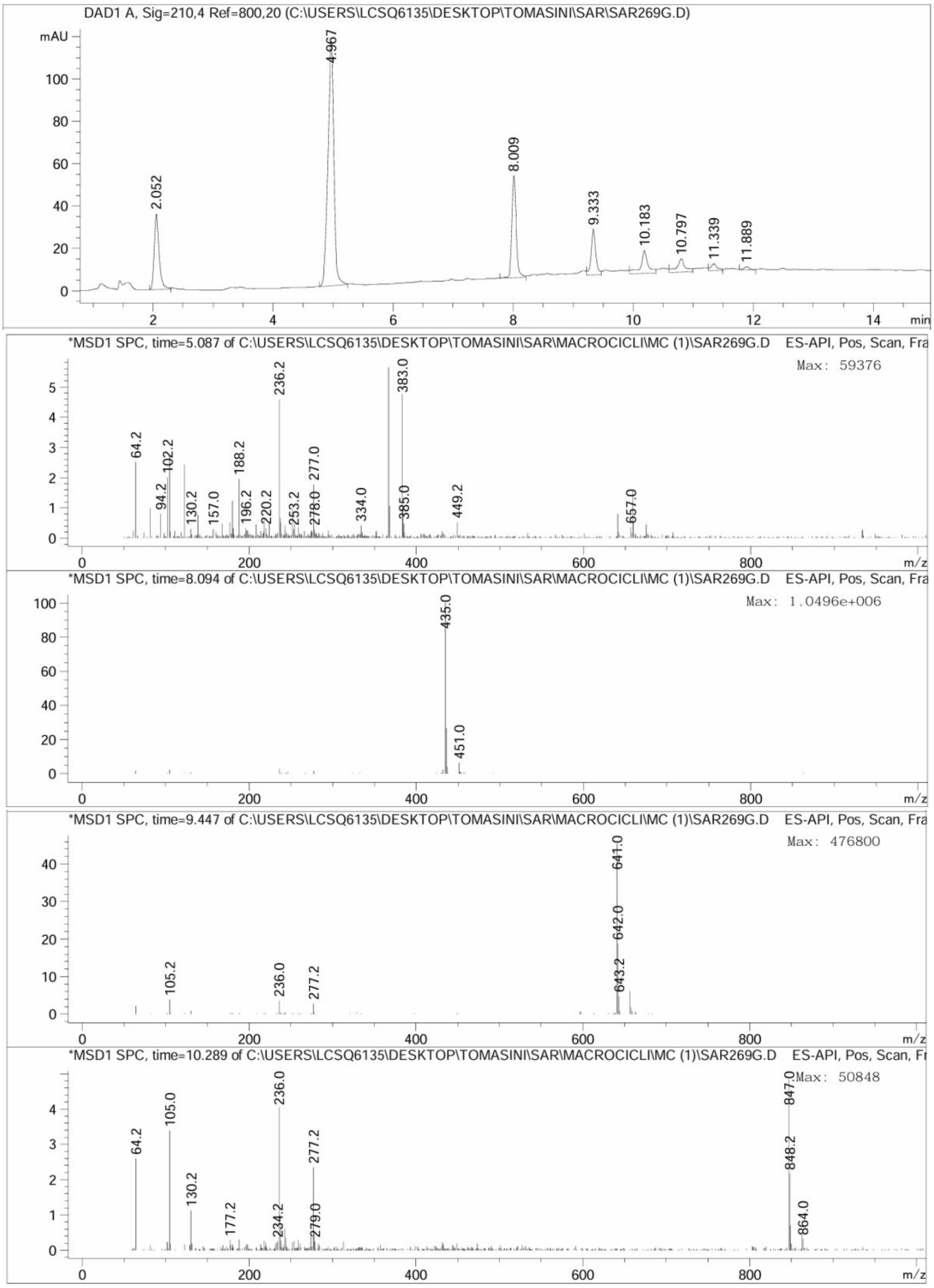

Table S2. HPLC-MS analysis of the crude reaction of macrocyclization for the formation of **2**

Peak Mc2	Macrocycle	Exact mass	Mass found	Ret. Time (min)	Area %
**1**	Dimeric	663.9	686.8 (M+Na$^+$), 702.8 (M+K$^+$),	7.596	67.1
**2**	Trimeric	995.9	1018.8 (M+Na$^+$)	9.289	25.4
**3**	Tetrameric	1327.8	1350.6 (M+Na$^+$)	10.417	7.5

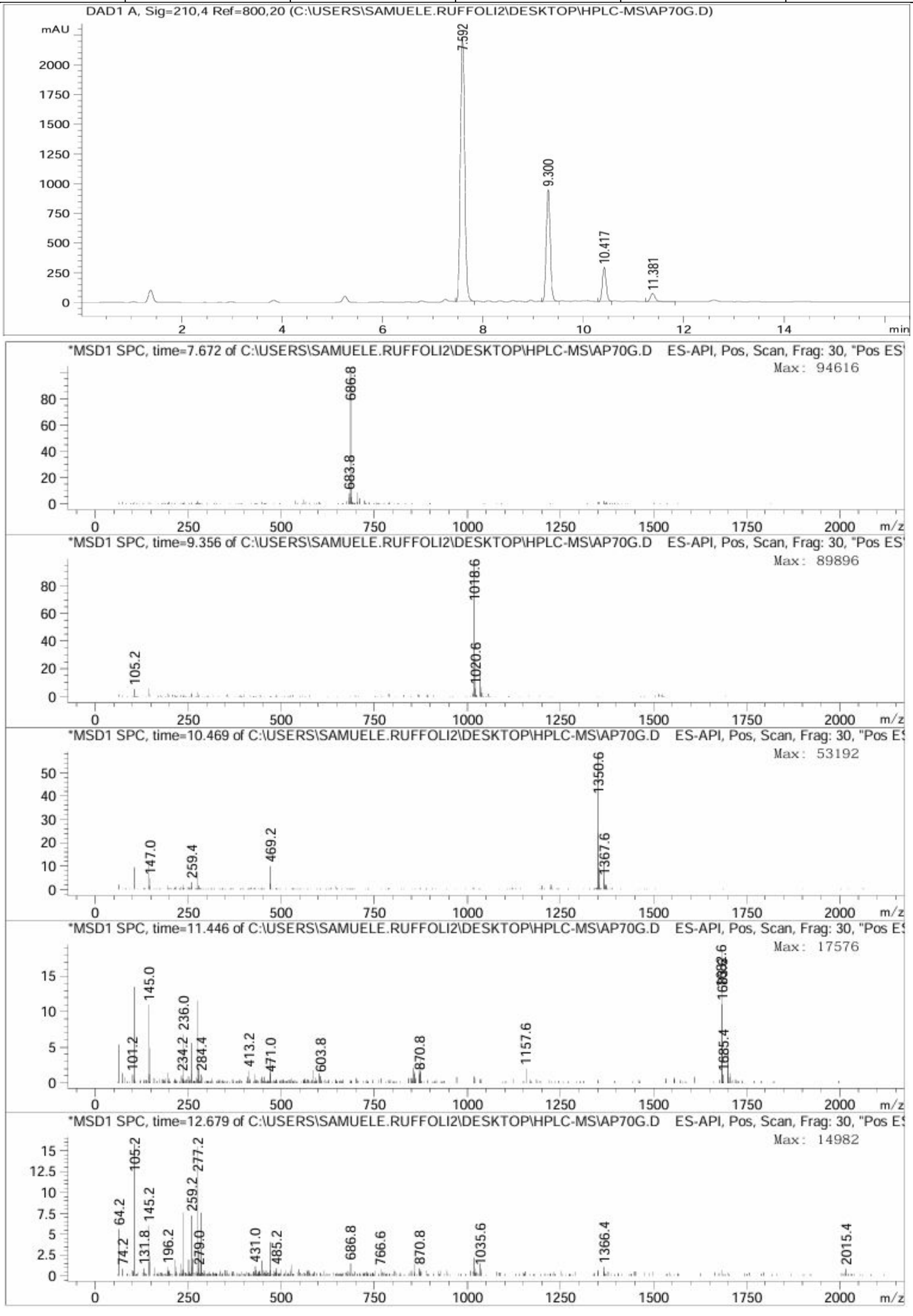

Table S3. HPLC-MS analysis of the crude reaction of macrocyclization for the formation of **3**

Peak Mc3	Macrocycle	Exact mass	Mass found	Ret. Time (min)	Area %
**1**	Dimeric	564.18	587 ($M+Na^+$)	11.056	65.5
**2**	Trimeric	846.27	869 ($M+Na^+$)	12.987	24.6
**3**	Tetrameric	1128.36	1151 ($M+Na^+$)	15.935	9.9

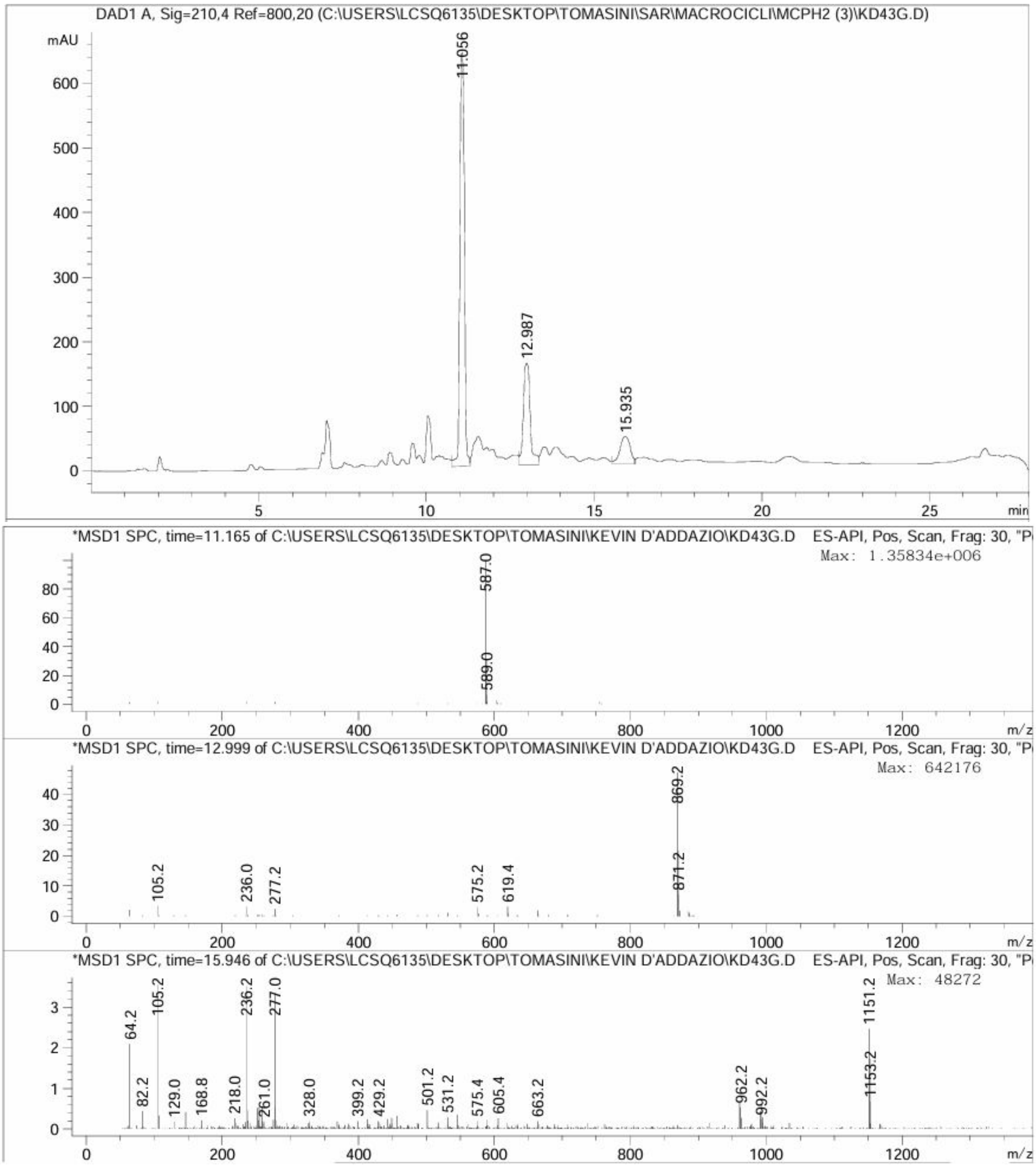

Table S4. HPLC-MS analysis of the crude reaction of macrocyclization for the formation of **4**

**Peak Mc3**	**Macrocycle**	**Exact mass**	**Mass found**	**Ret. Time (min)**	**Area %**
**1**	Dimeric	815.97	838.8 (M+Na$^+$), 854.8 (M+K$^+$)	10.06	63.7
**2**	Trimeric	1223.96	1246.8 (M+Na$^+$)	11.954	26.4
**3**	Tetrameric	1631.93	-	-	-

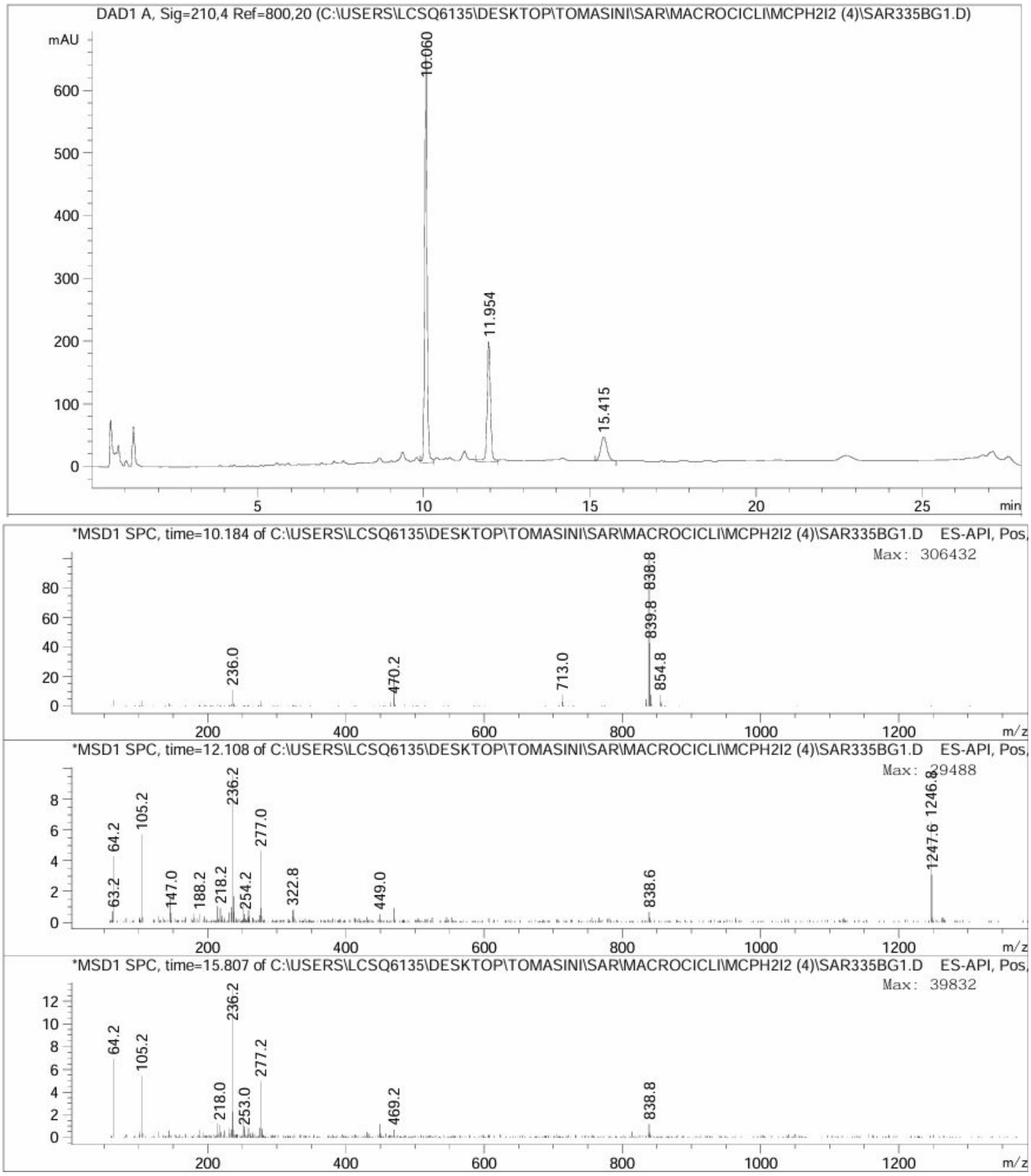

Table S5. HPLC-MS analysis of the crude reaction of macrocyclization for the formation of **5**

Peak Mc5	Macrocycle	Exact mass	Mass found	Ret. Time (min)	Area %
**1**	Dimeric	883.89	906.8 (M+Na$^+$)	10.23	72.0
**2**	Trimeric	1325.84	1348.6 (M+Na$^+$)	15.44	28.0
**3**	Tetrameric	1767.8	-	-	-

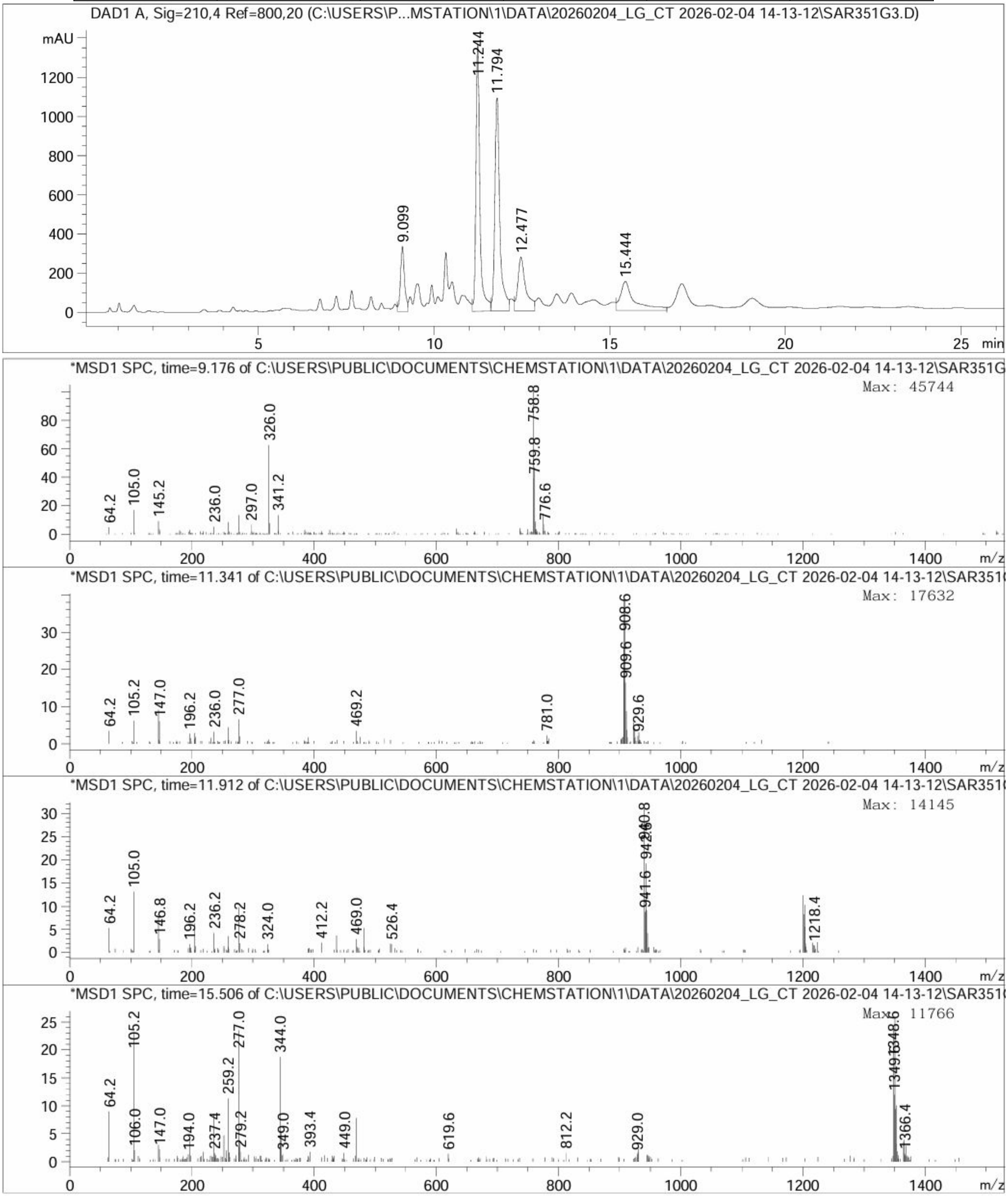

Table S6. HPLC-MS analysis of the crude reaction of macrocyclization for the formation of **6**

Peak Mc6	Macrocycle	Exact mass	Mass found	Ret. Time (min)	Area %
**1**	Dimeric	844	866.8 (M+Na$^+$), 882.8 (M+K$^+$)	10.85	69.8
**2**	Trimeric	1266	1288.8 (M+Na$^+$), 1304.8 (M+K$^+$)	14.64	21.7
**3**	Tetrameric	1184.4	1711.6(M+Na$^+$)	24.27	8.5

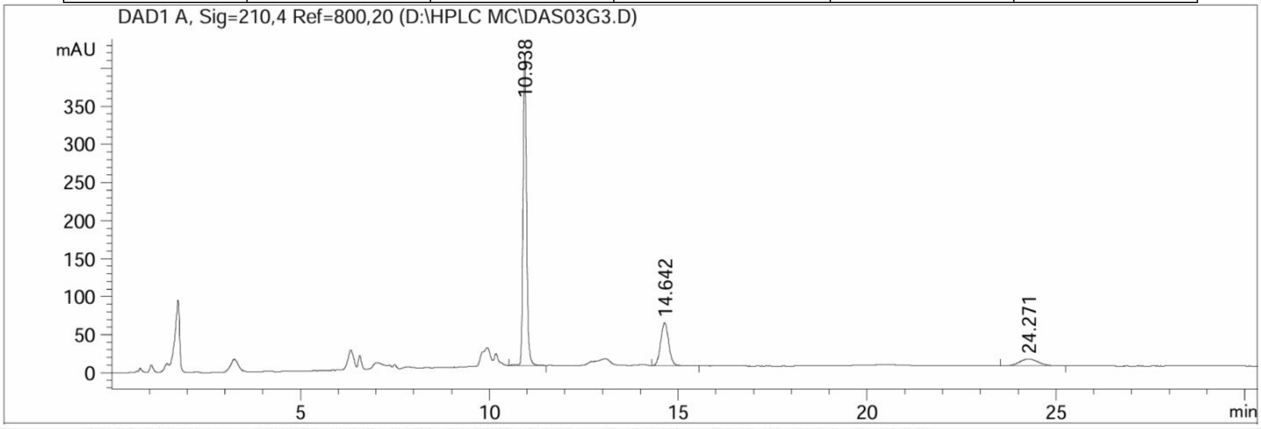

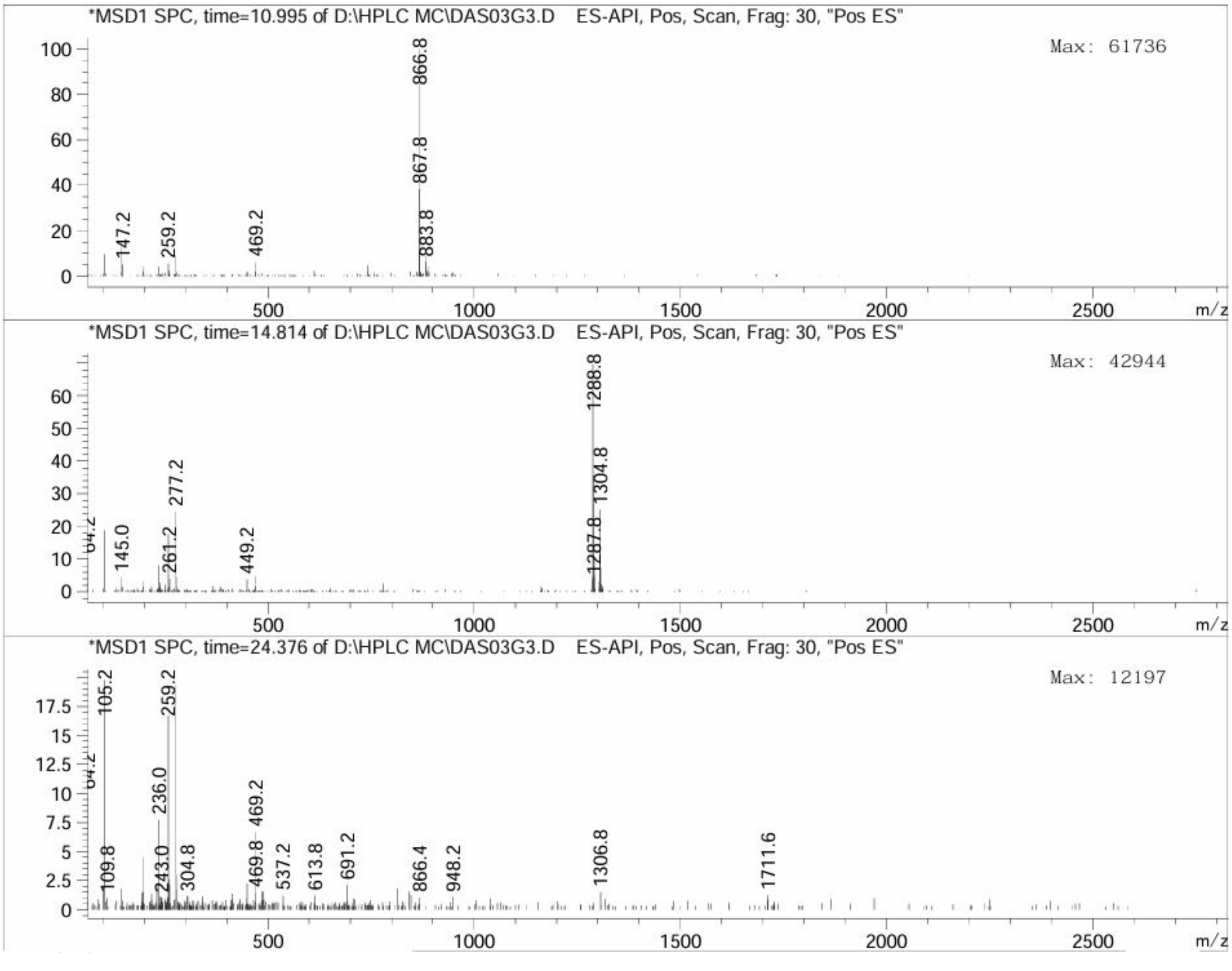

Table S7. Crystal data and refinement details for compounds **1-7** collected at room temperature (293-300K).

	**1**	**2**	**3**	**4**	**5**	**6**	**7**
**Formula**	$C_{22}H_{20}O_8$	$C_{22}H_{18}I_2O_8$	$C_{34}H_{28}O_8$	$C_{34}H_{26}I_2O_8$	$C_{34}H_{24}O_8Cl_2I_2$	$C_{36}H_{30}O_8I_2$	$C_{38}H_{28}O_8$
**FW (g/mol)**	412.38	664.16	564.56	816.35	885.23	844.40	612.60
**Cryst. Sys.**	monoclinic	monoclinic	monoclinic	triclinic	monoclinic	triclinic	triclinic
**Space Group**	$P2_1/n$	$P2_1/c$	$P2_1/n$	P-1	$P2_1/m$	P-1	P1
**a/Å**	10.6492(5)	14.3357(12)	11.5746(6)	8.0110(5)	8.7613(6)	5.2930(3)	8.31400(10)
**b/Å**	6.5607(3)	4.7954(3)	5.2517(3)	8.3985(5)	13.5025(9)	11.0118(6)	8.3336(2)
**c/Å**	15.0289(8)	16.3842(15)	23.1328(14)	11.9304(8)	13.9599(9)	14.9487(8)	11.7250(2)
**α/°**	90	90	90	86.760(5)	90	77.226(2)	71.5830(10)
**β/°**	110.647(6)	98.436(8)	96.265(5)	86.643(5)	94.732(2)	86.790(2)	86.3100(10)
**γ/°**	90	90	90	84.617(5)	90	87.157(2)	85.4330(10)
**Volume/Å^3**	982.56(9)	1114.15(16)	1397.76(14)	796.72(9)	1645.82(19)	847.77(8)	767.66(2)
Z	2	2	2	1	2	1	1
**ρ_{calc} g/cm^3**	1.394	1.980	1.341	1.701	1.786	1.654	1.325
**μ/mm^{-1}**	0.107	2.869	0.096	2.024	2.124	1.905	0.093
**measd rflns**	7651	9396	10053	6380	78333	47821	57363
**indep rflns**	2385	2673	3297	3636	3527	3496	7593
**R_1**	0.0501	0.0494	0.0852	0.0774	0.0557	0.0367	0.0699
**wR_2**	0.1357	0.0922	0.2136	0.2168	0.1742	0.1078	0.2336

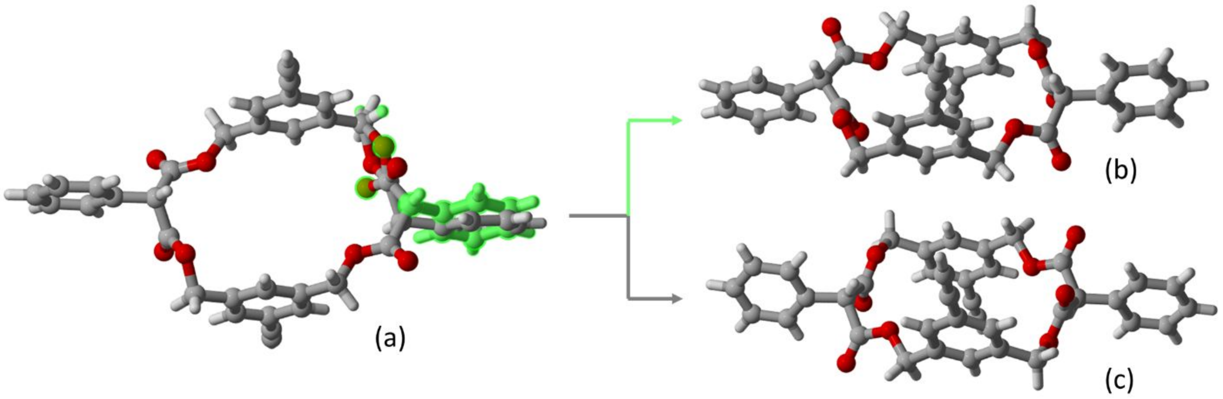

Figure S46. (a) The asymmetric unit detected in crystalline **7** with the conformational disorder observed and corresponding to the cis isomer highlighted in green. (b) molecular structure of **7-cis**, and (b) **7-trans**.

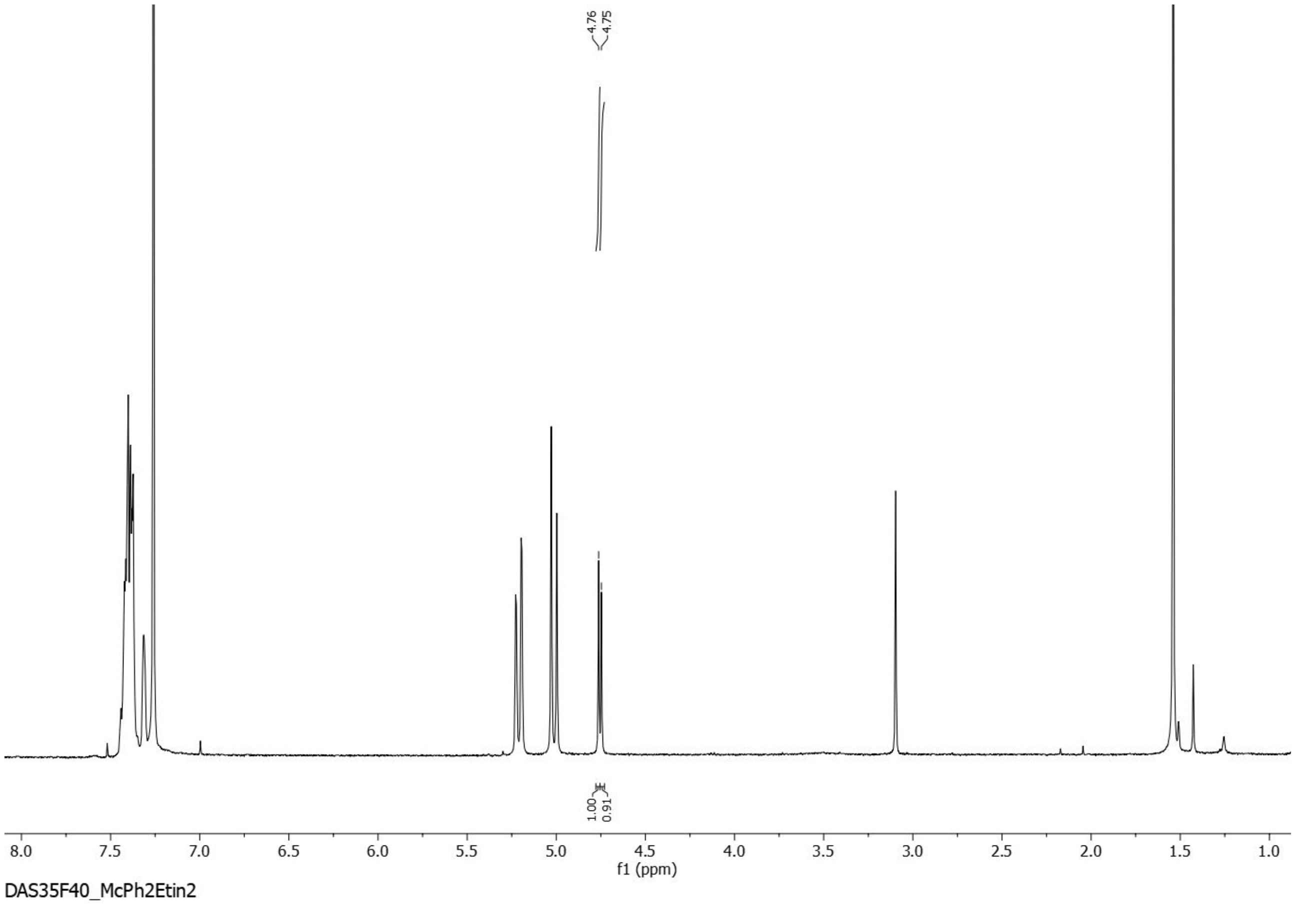

Figure S47. ^{1}H NMR spectrum of dissolved crystals obtained from **7**. Two singlets at 7.76 and 7.75 ppm, corresponding to the α-protons of the malonyl moiety, integrate in a 1:0.9 ratio, indicating the presence of two co-crystallized diastereoisomers.

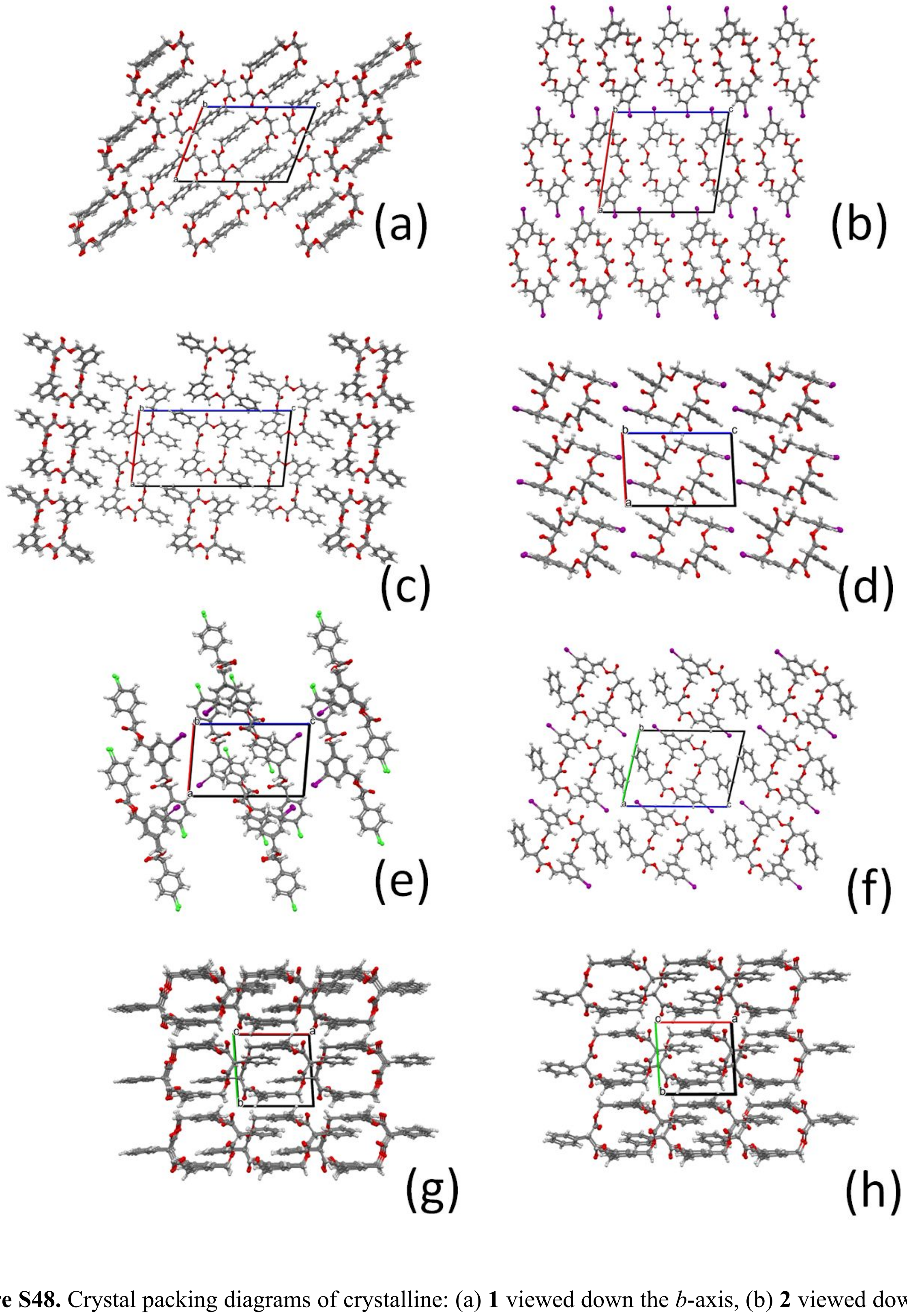

Figure S48. Crystal packing diagrams of crystalline: (a) **1** viewed down the *b*-axis, (b) **2** viewed down the *b*-axis, (c) **3** viewed down the *b*-axis, (d) **4** viewed down the *b*-axis, (e) **5** viewed down the *b*-axis, (f) **6** viewed down the *a*-axis, (g) **7**-cis viewed down the *c*-axis and, (h) **7**-trans viewed down the *c*-axis.

Table S8. Electrostatic, polarization, dispersion, repulsion terms and total interaction energy within the pairs of molecules shown in Figure 5 for crystalline: (a) **7-cis** and (b) **7-trans**.

		R (Å)[a]	$E_{ele.}$ (kJ/mol)	$E_{pol.}$(kJ/mol)	$E_{disp.}$(kJ/mol)	$E_{rep.}$(kJ/mol)	$E_{tot.}$(kJ/mol)
**7-cis**	red	8.31	-32.1	-11.9	-140.6	92.7	-107.8
	yellow	11.29	-12.3	-3.6	-51.7	18.7	-49.1
	green	8.33	-7.1	-3.8	-27.1	13.3	-25.6
	cyan	11.73	0.9	-3.2	-16.7	12.7	-8.1
	blue	13.93	-4.7	-1.5	-23.2	0.0	-26.4
	magenta	12.05	1.0	-1.7	-27.2	0.0	-23.8
**7-trans**	red	8.31	-23.2	-7.8	-136.7	74.9	-103.1
	yellow	11.29	-17.3	-4.4	-60.2	31.0	-54.9
	green	8.33	-12.9	-4.9	-27.4	14.9	-31.9
	cyan	11.73	-6.0	-3.1	-15.3	8.0	-17.1
	blue	13.93	3.7	-2.1	-23.9	0.0	-18.4
	magenta	12.05	1.3	-1.6	-26.5	0.0	-22.9

[a]= distance between molecular centroids (mean atomic positions).

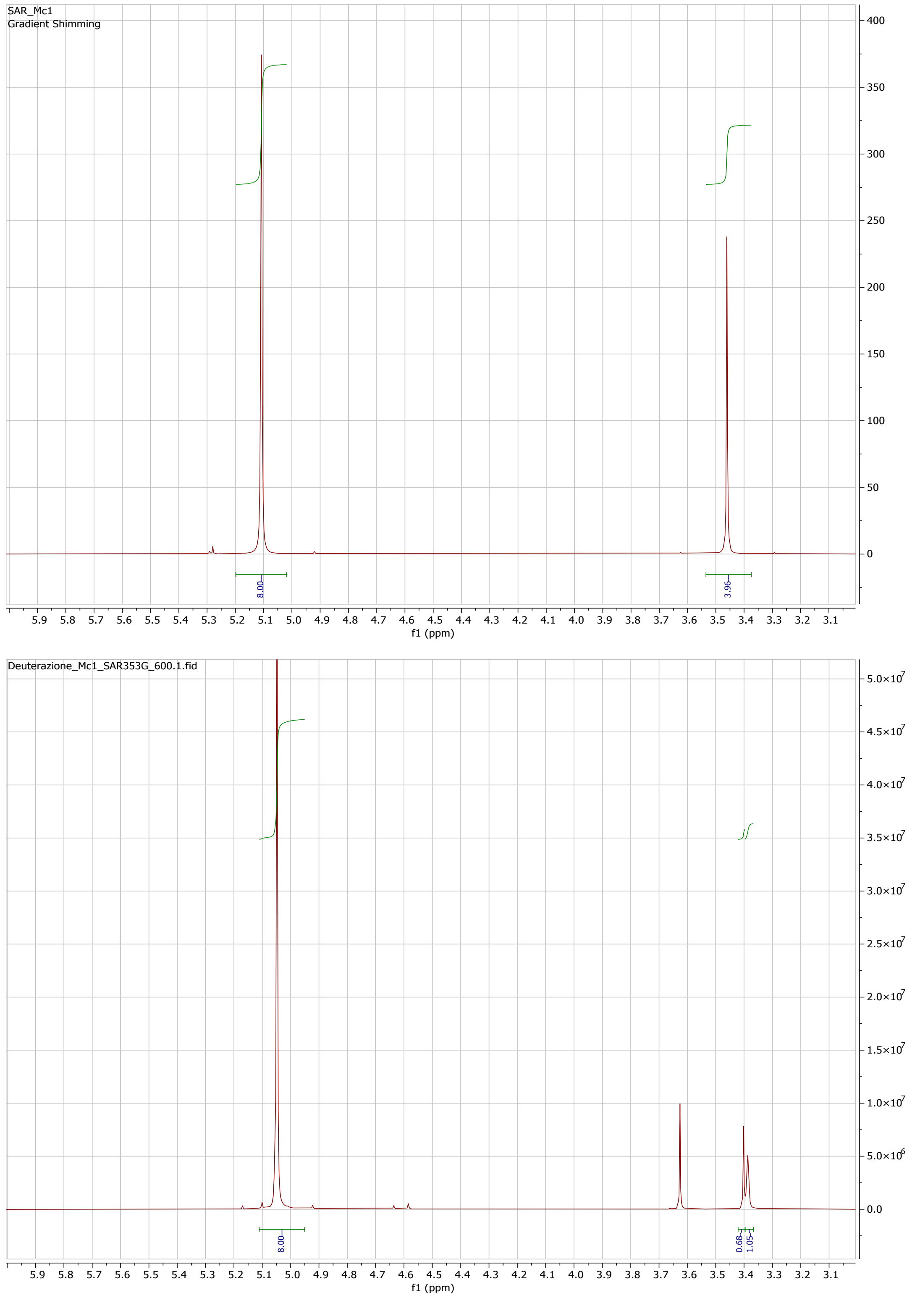

Figure S49. Details of the ^{1}H NMR spectra of **1** before (top) and after (bottom) deprotonation with *t*Bu-OK followed by deuteration with DCl in D_2O.

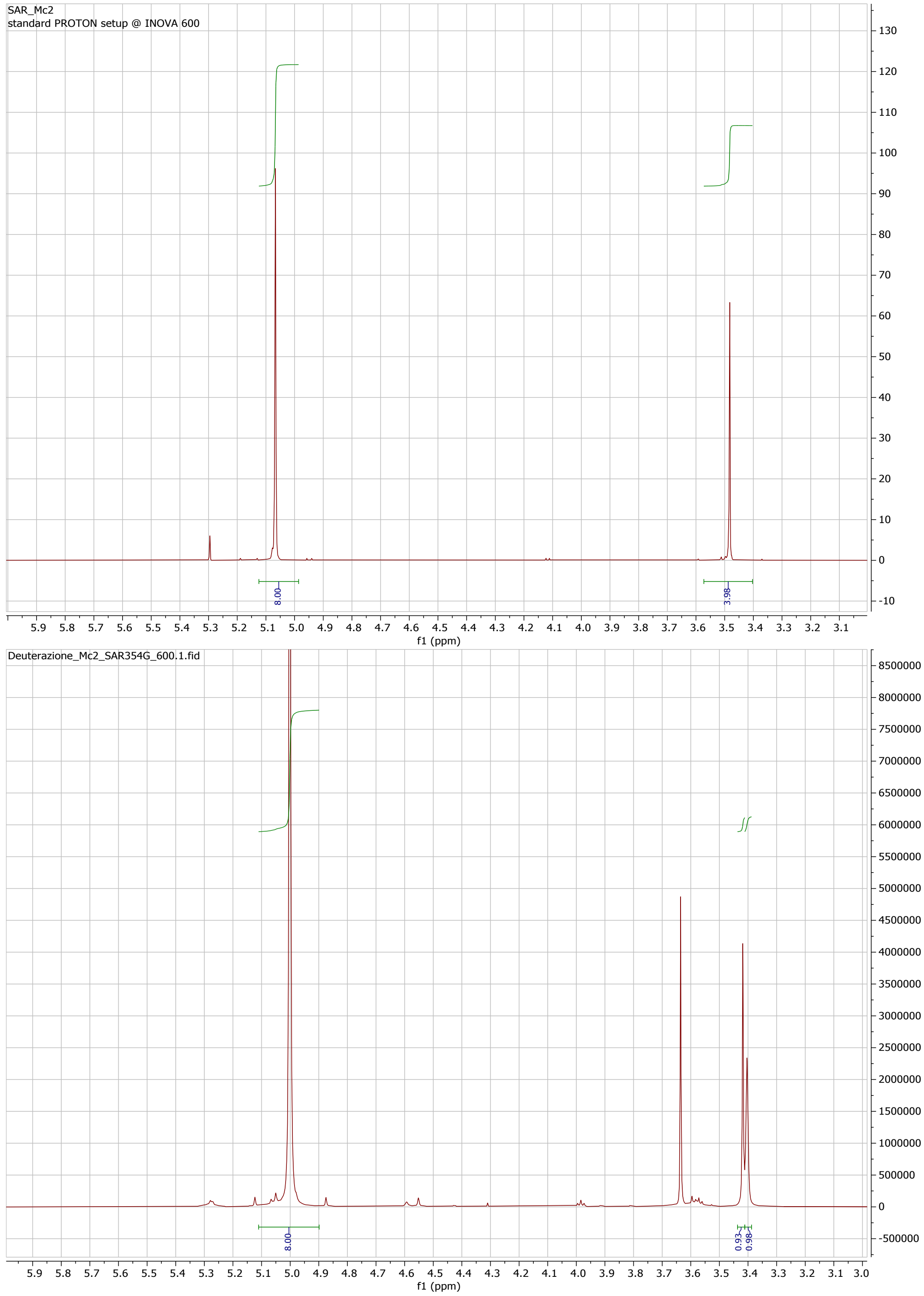

Figure S50. Details of the ^{1}H NMR spectra of **2** before (top) and after (bottom) deprotonation with *t*Bu-OK followed by deuteration with DCl in D_2O.

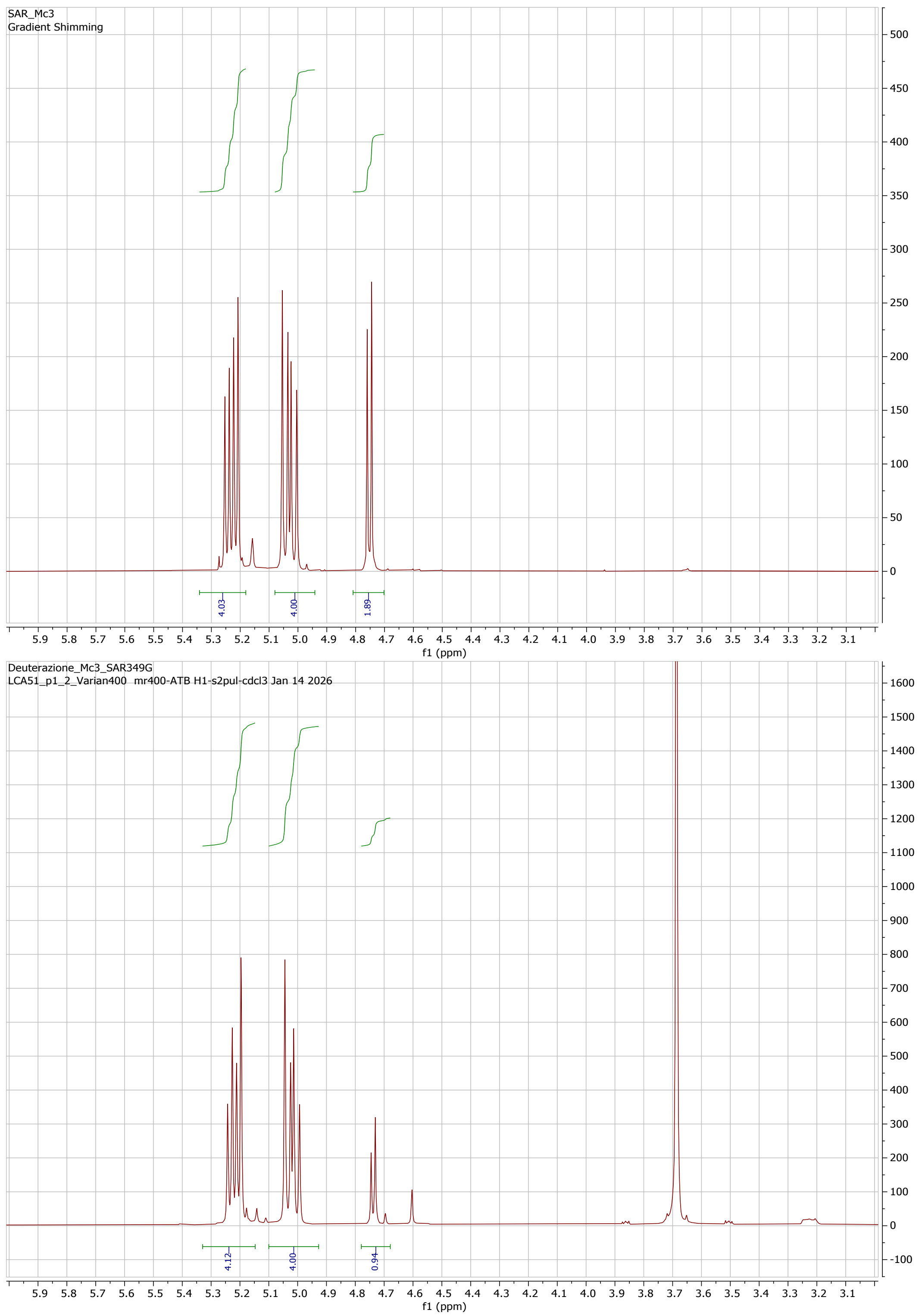

Figure S51. Details of the ^{1}H NMR spectra of **3** before (top) and after (bottom) deprotonation with *t*Bu-OK followed by deuteration with DCl in D_2O.

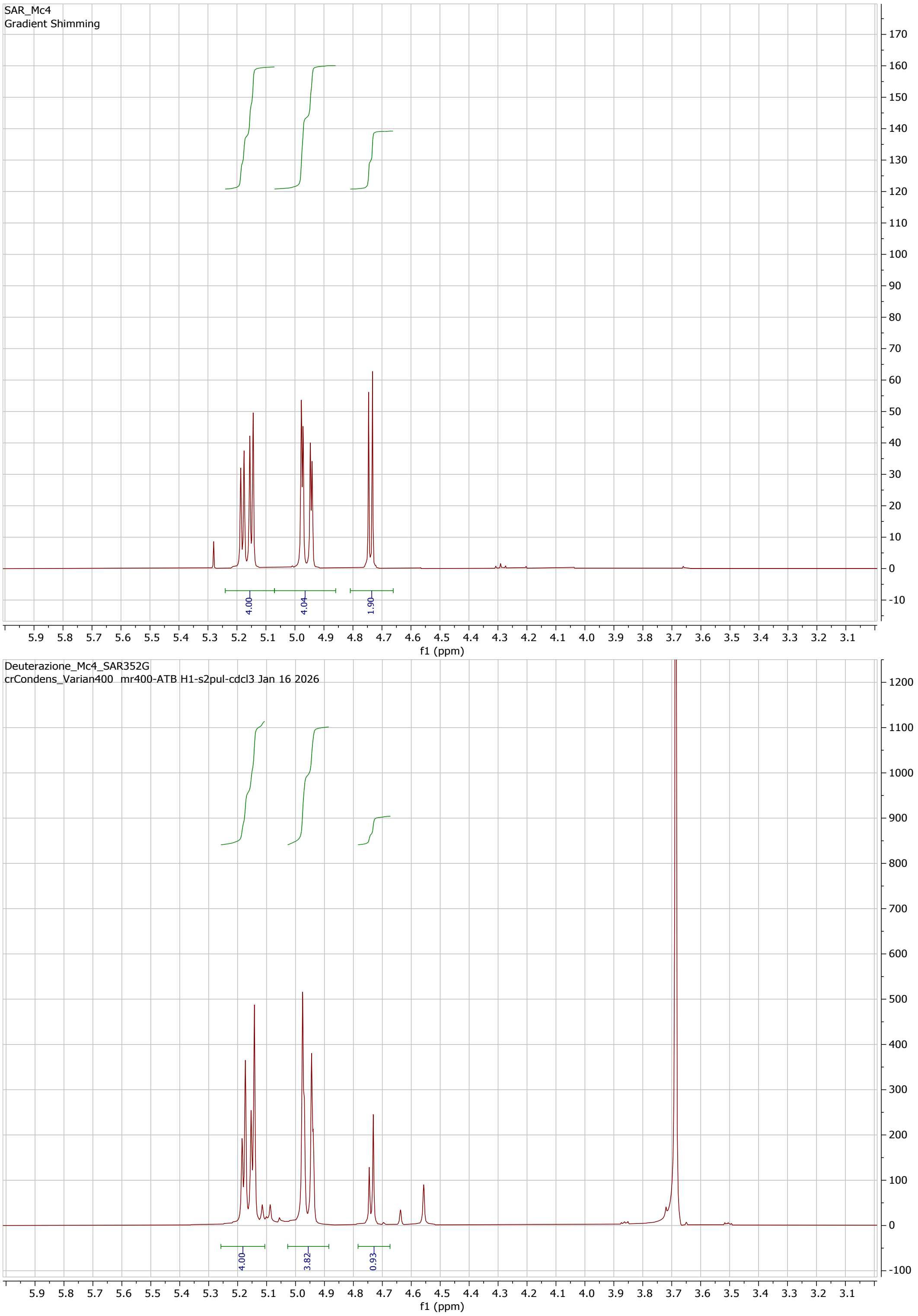

Figure S52. Details of the ^{1}H NMR spectra of **4** before (top) and after (bottom) deprotonation with *t*Bu-OK followed by deuteration with DCl in D_2O.

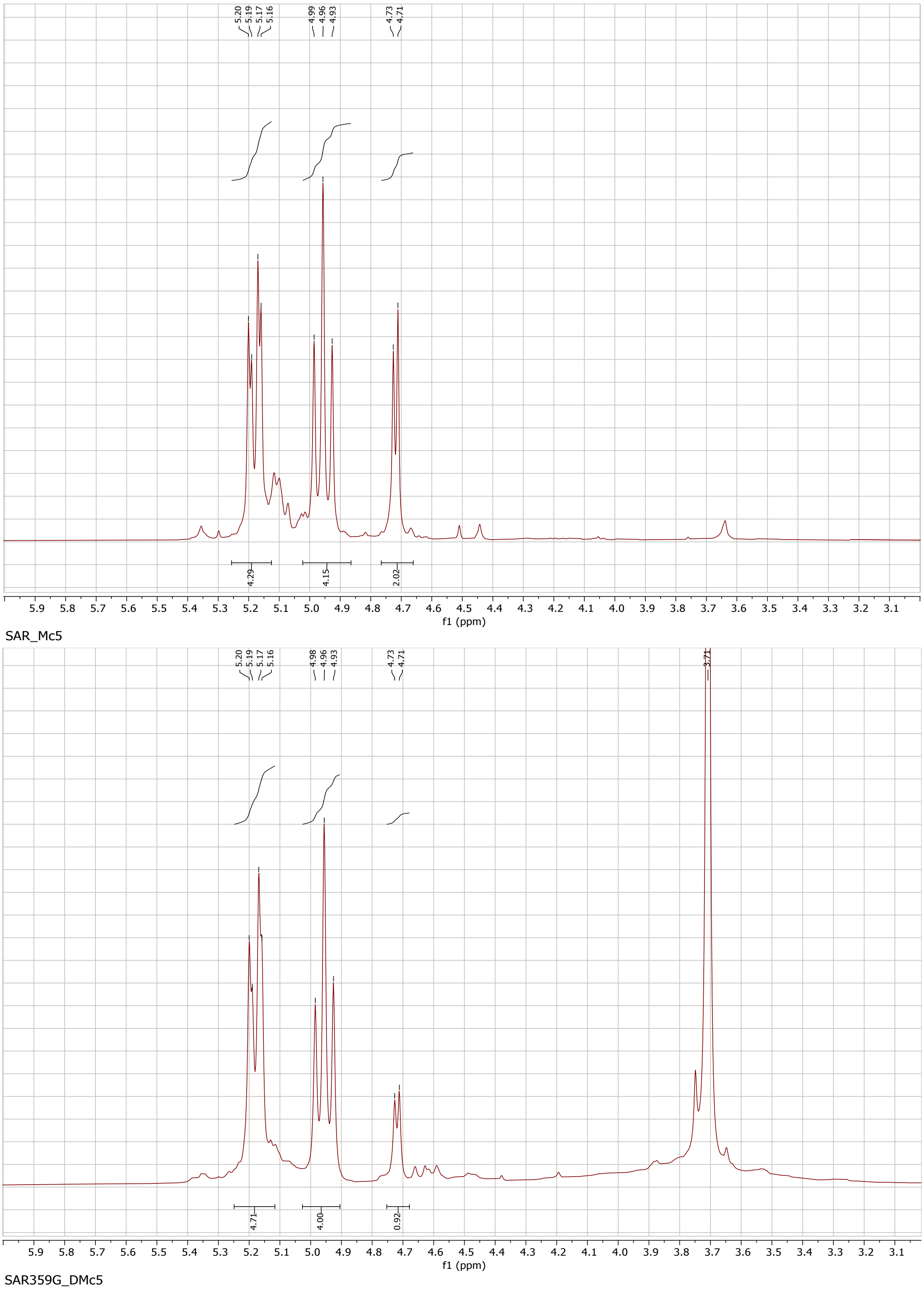

Figure S53. Details of the ^{1}H NMR spectra of **5** before (top) and after (bottom) deprotonation with *t*Bu-OK followed by deuteration with DCl in D_2O.

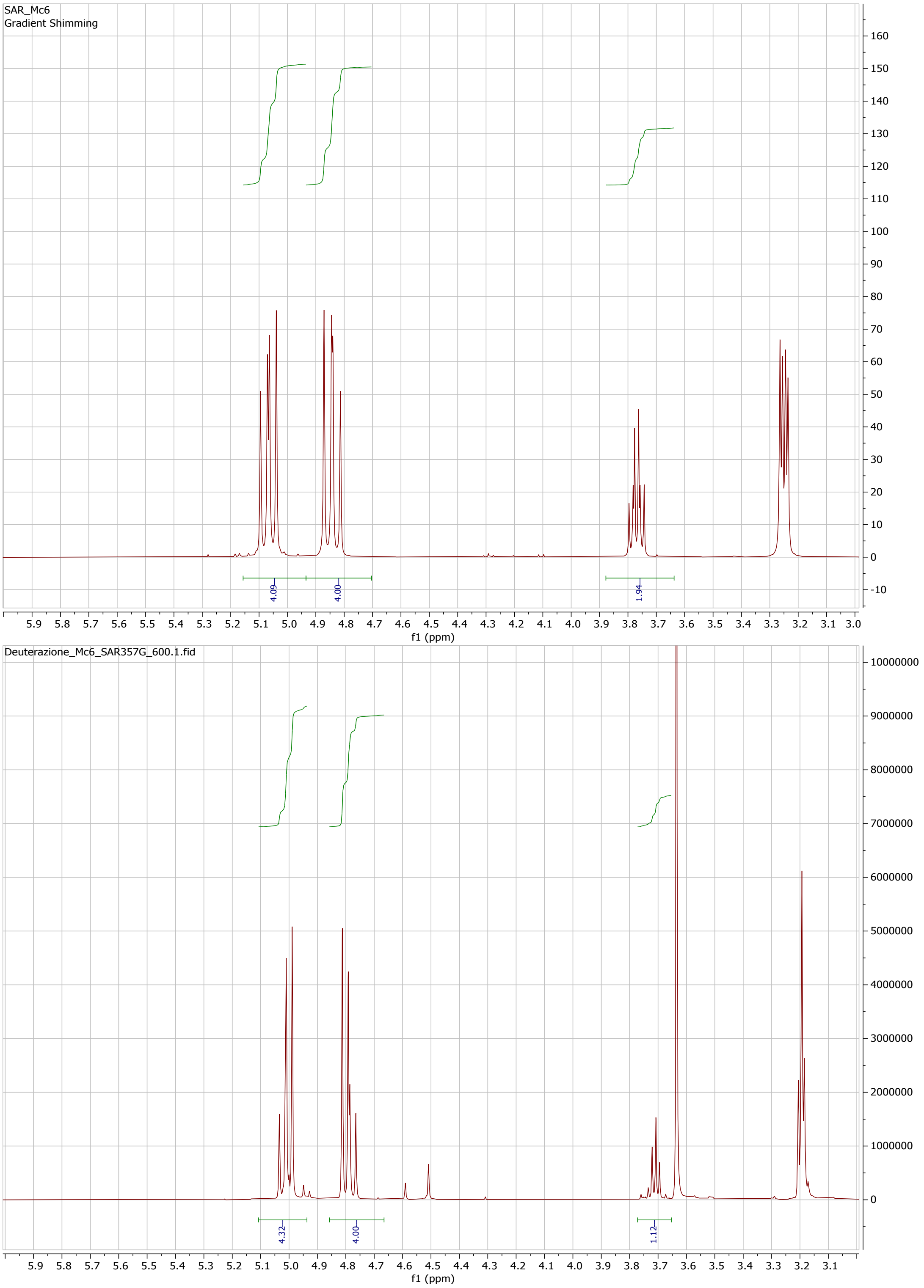

Figure S54. Details of the ^{1}H NMR spectra of **6** before (top) and after (bottom) deprotonation with *t*Bu-OK followed by deuteration with DCl in D_2O.

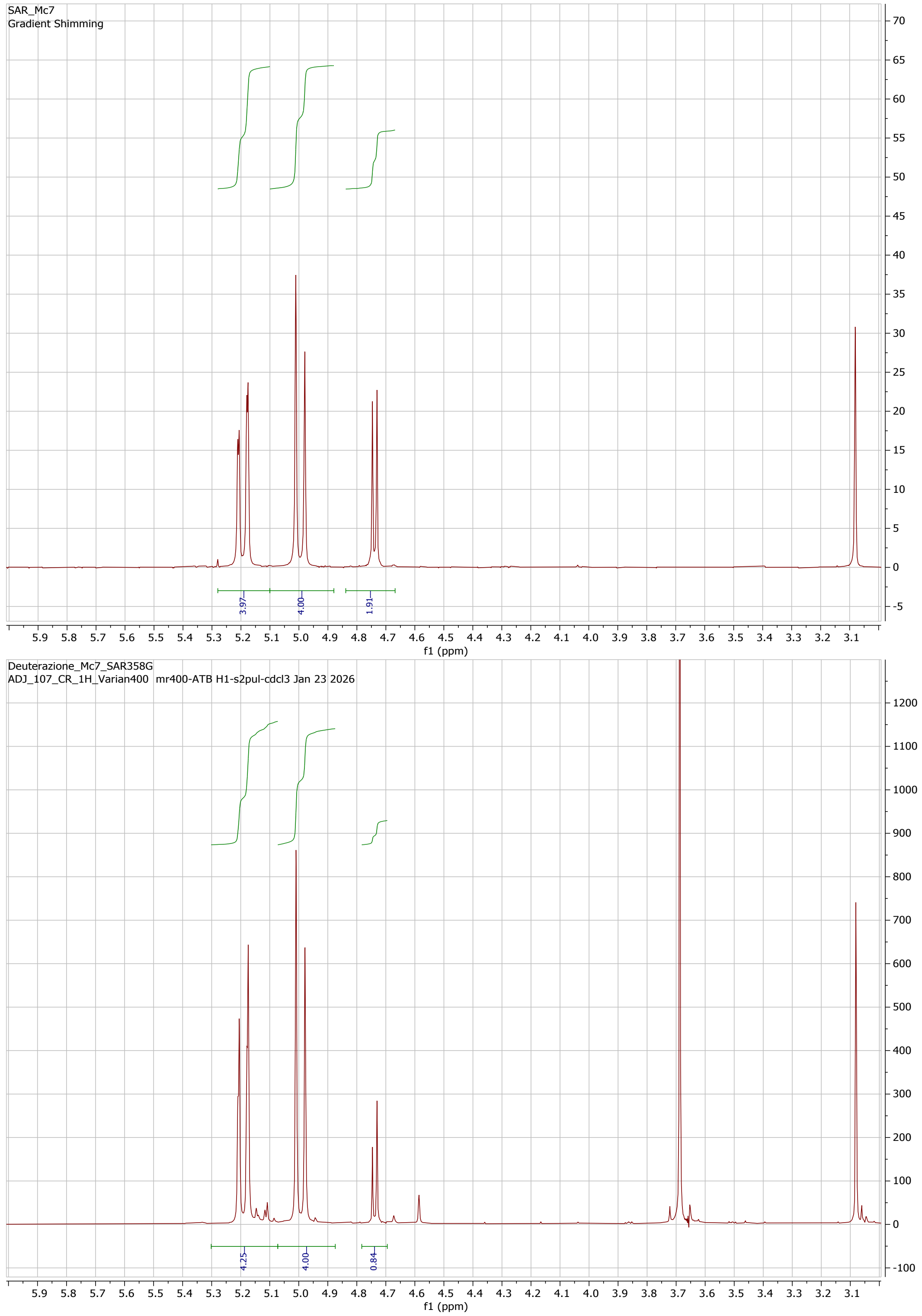

Figure S55. Details of the ^{1}H NMR spectra of **7** before (top) and after (bottom) deprotonation with *t*Bu-OK followed by deuteration with DCl in D_2O.

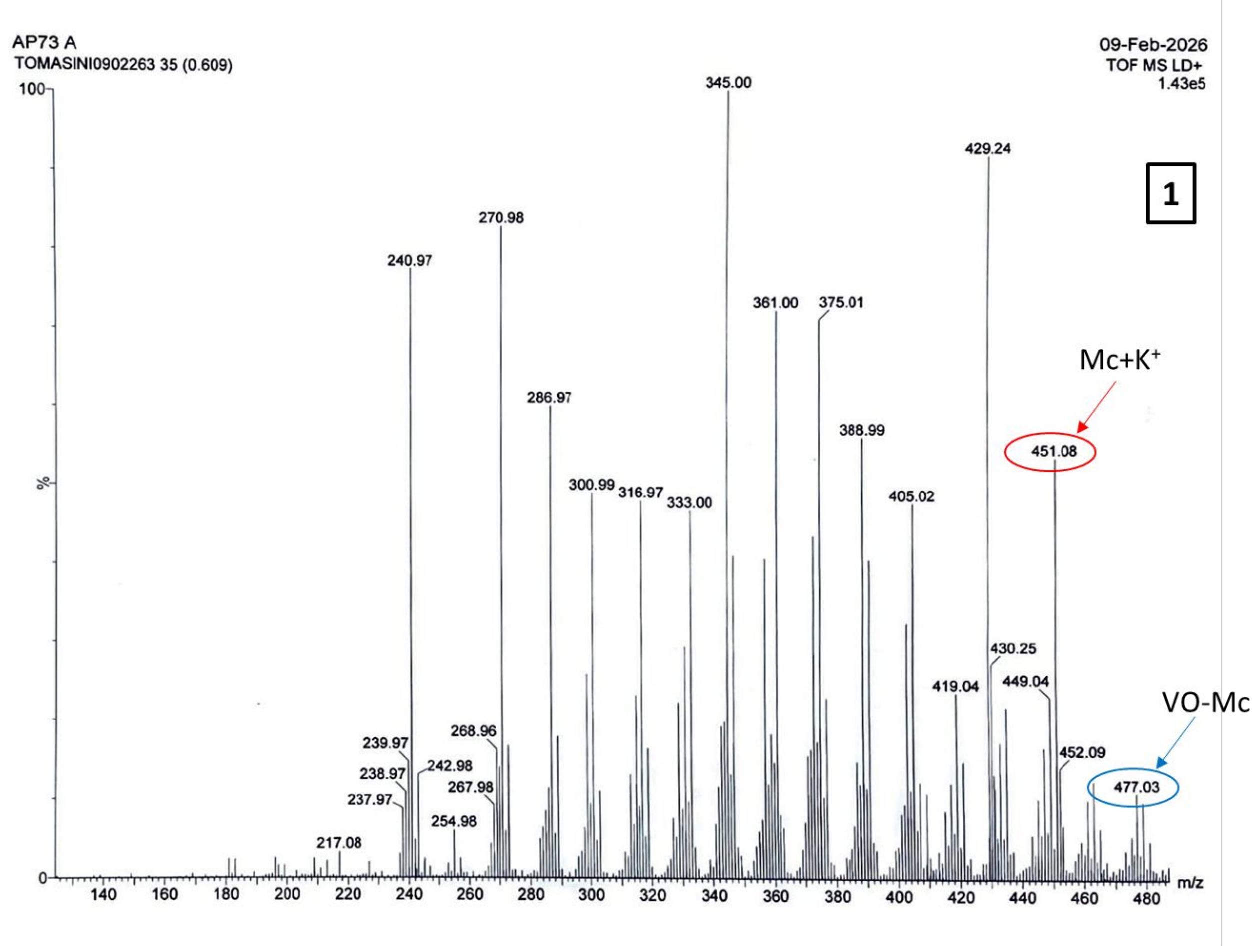

Figures S56. MALDI-TOF Analysis of the Vanadyl Complex of **1**.

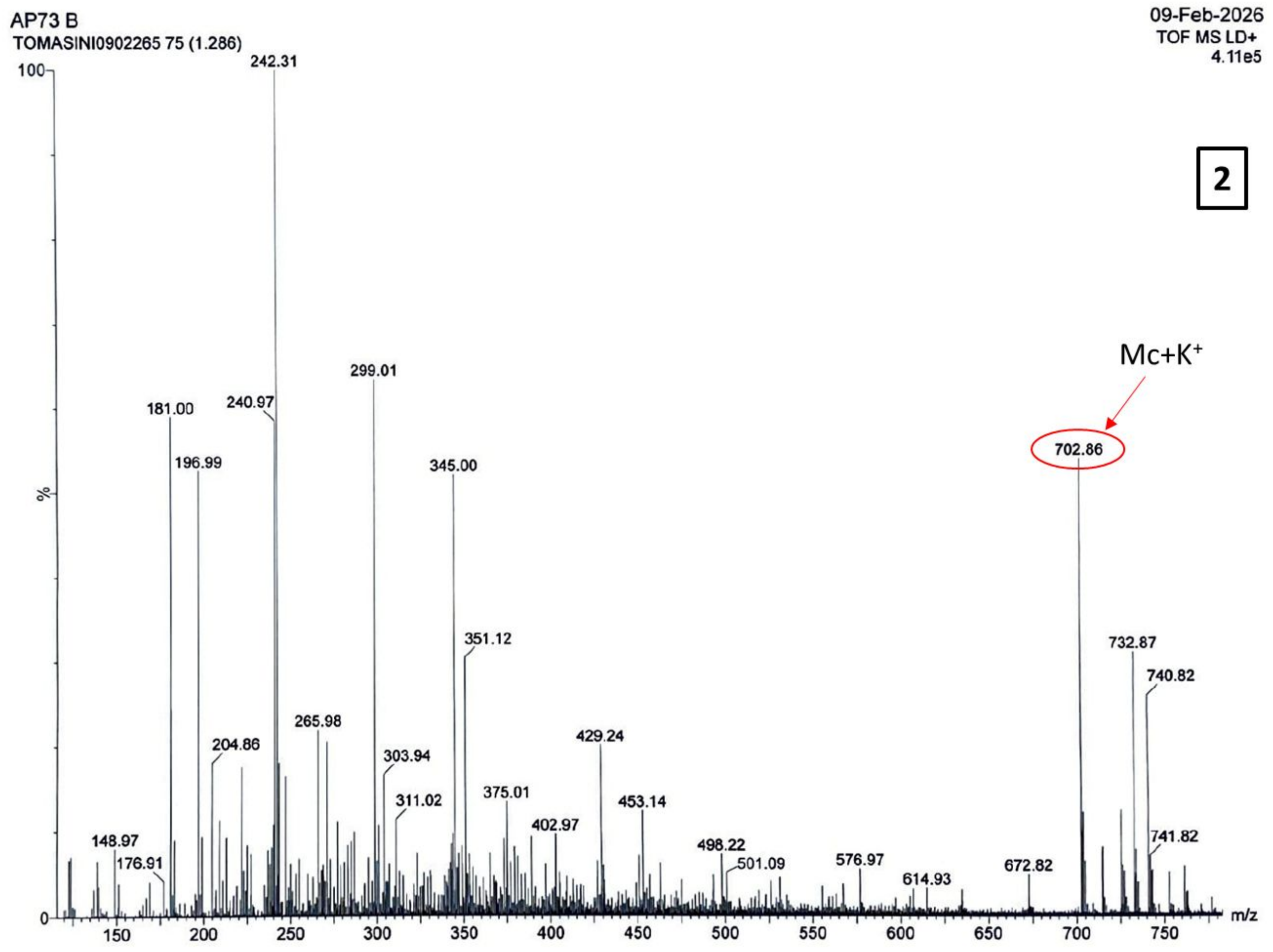

Figures S57. MALDI-TOF Analysis of the Vanadyl Complex of **2**.

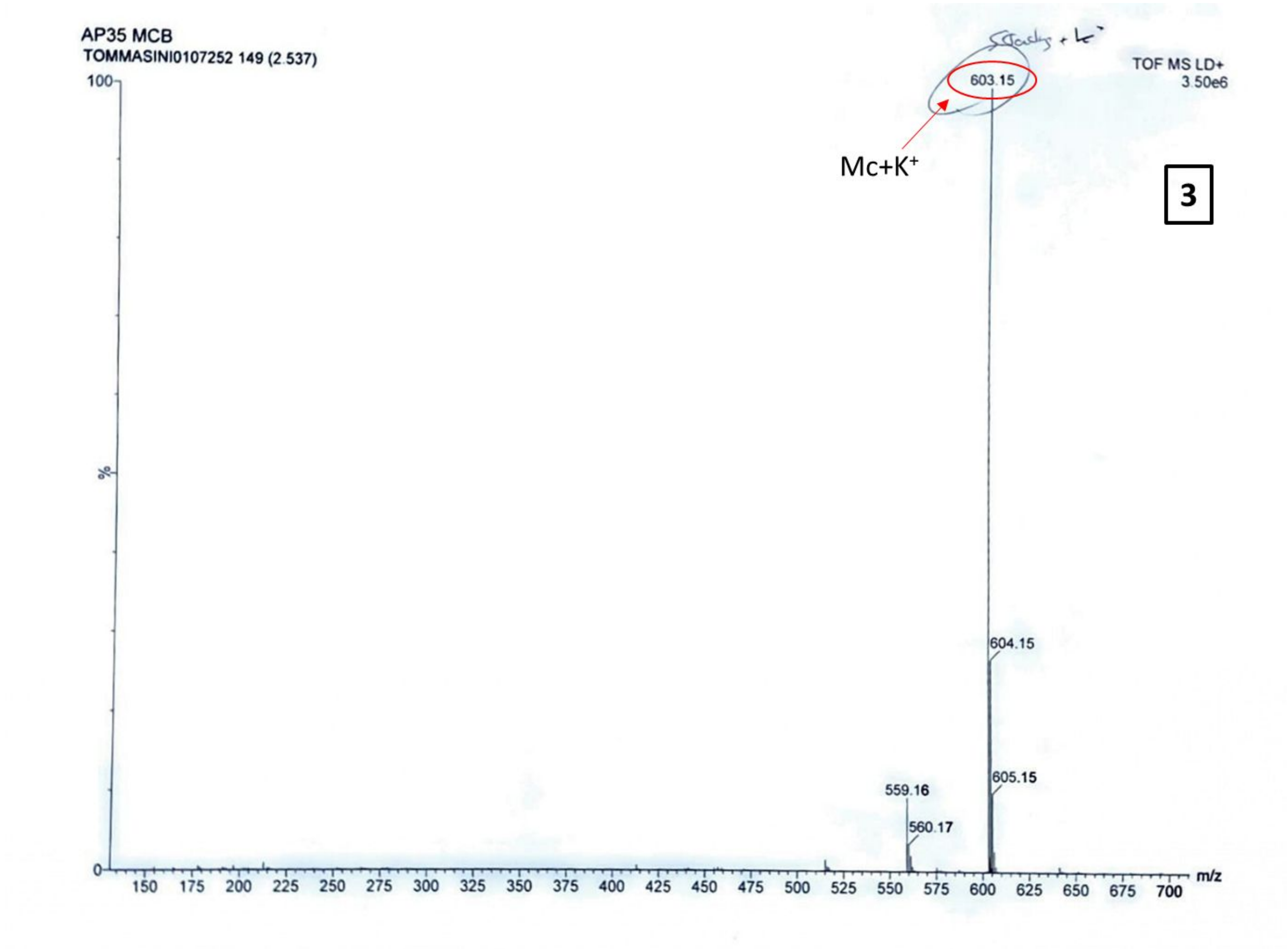

Figures S58. MALDI-TOF Analysis of the Vanadyl Complex of **3**.

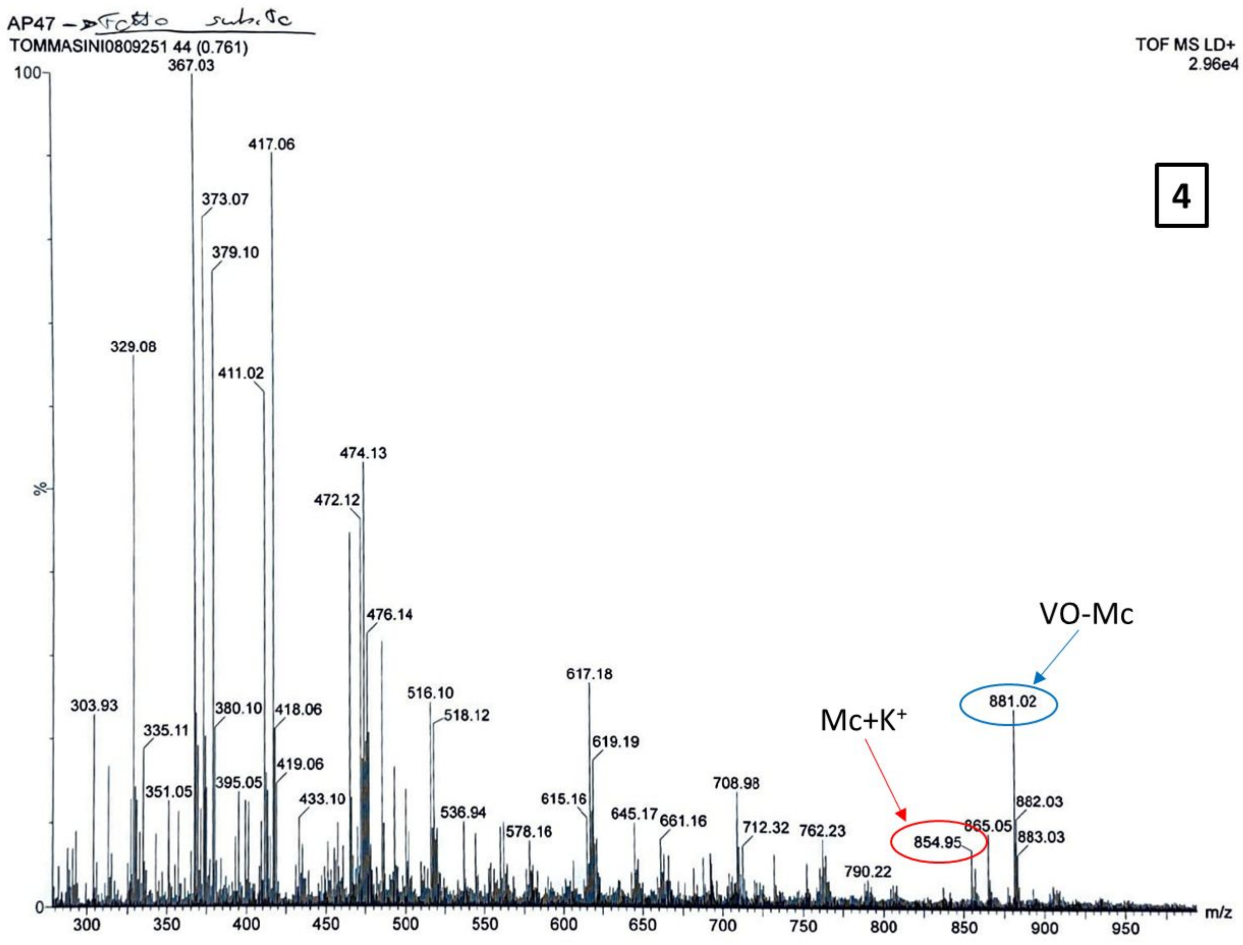

Figures S59. MALDI-TOF Analysis of the Vanadyl Complex of **4**.

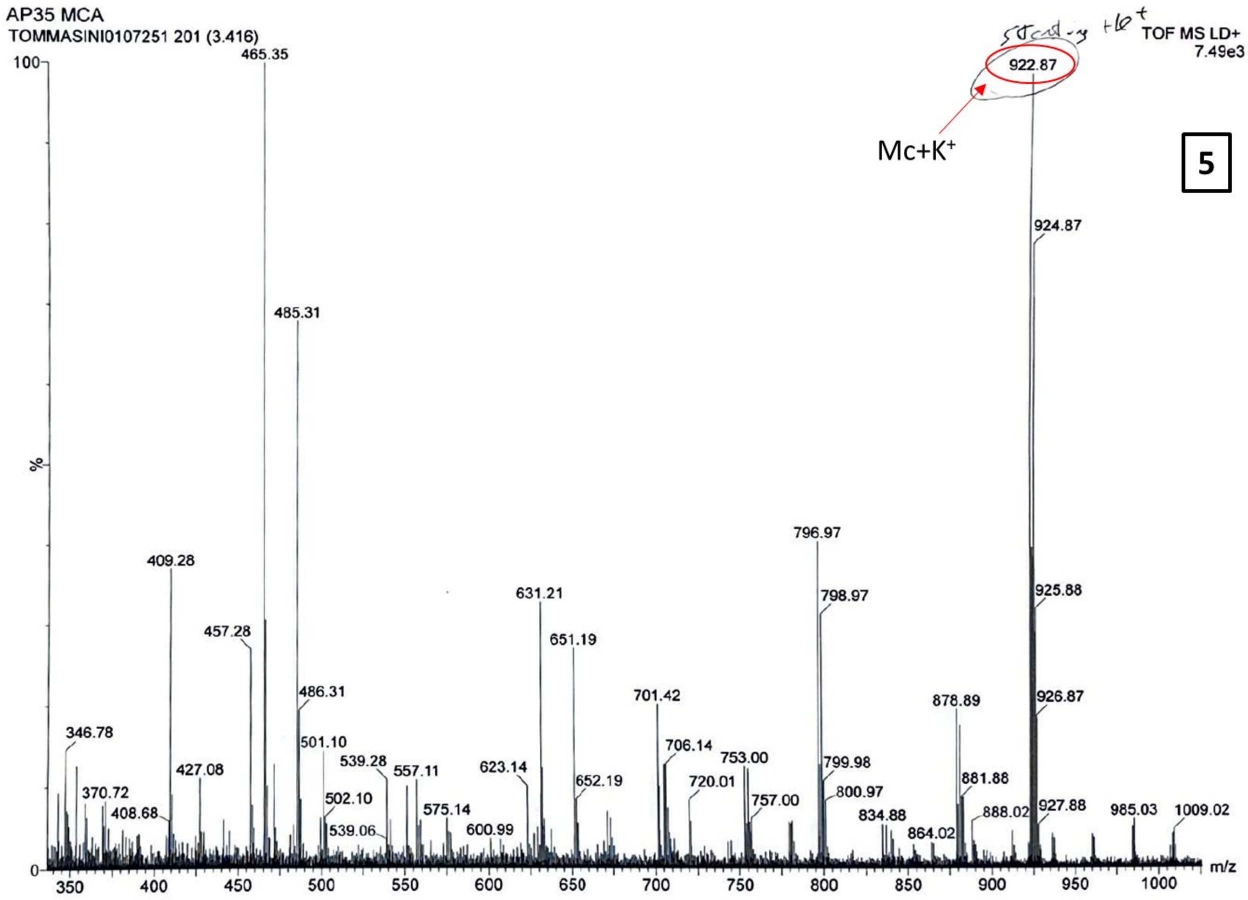

Figures S60. MALDI-TOF Analysis of the Vanadyl Complex of **5**.

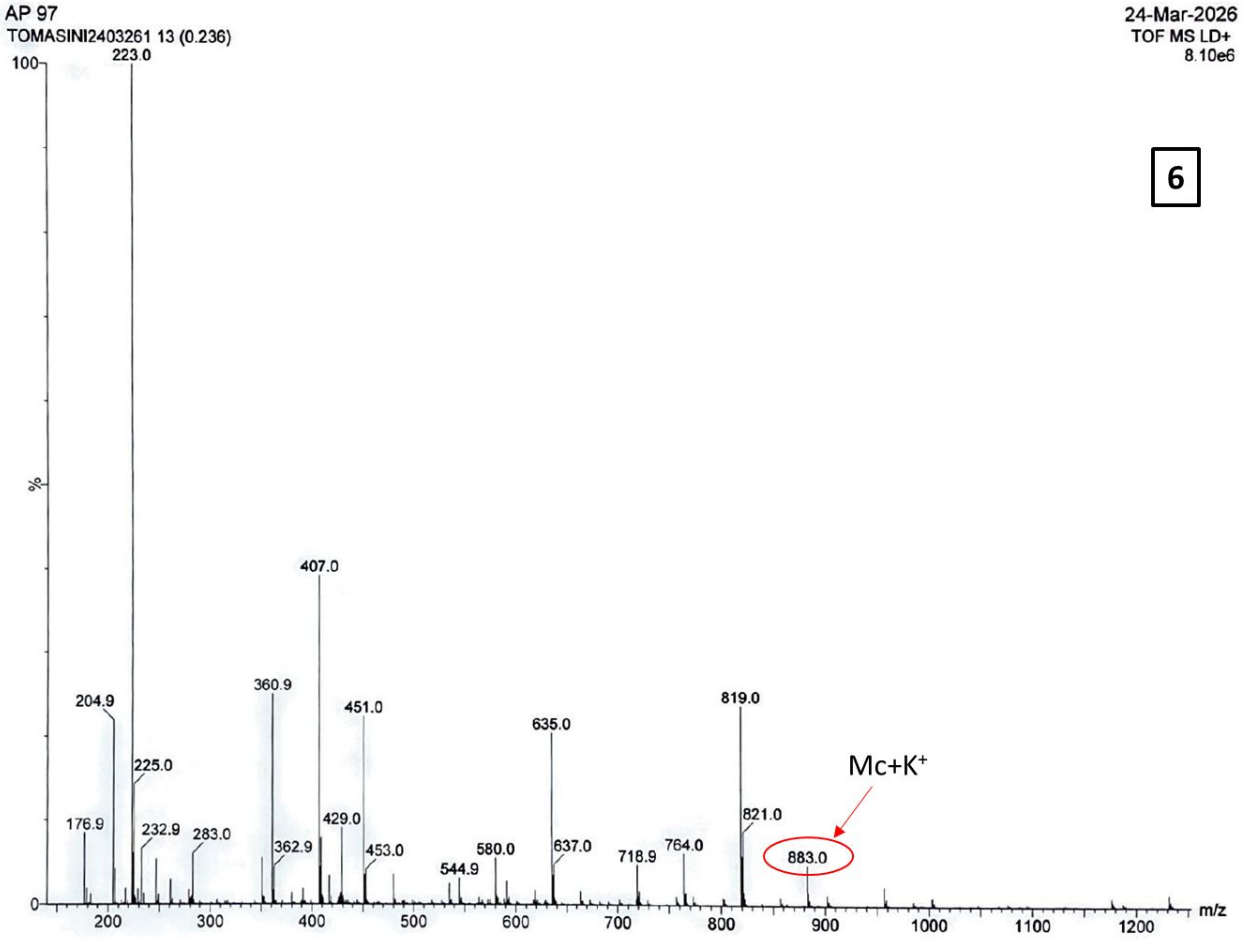

Figures S61. MALDI-TOF Analysis of the Vanadyl Complex of **6**.

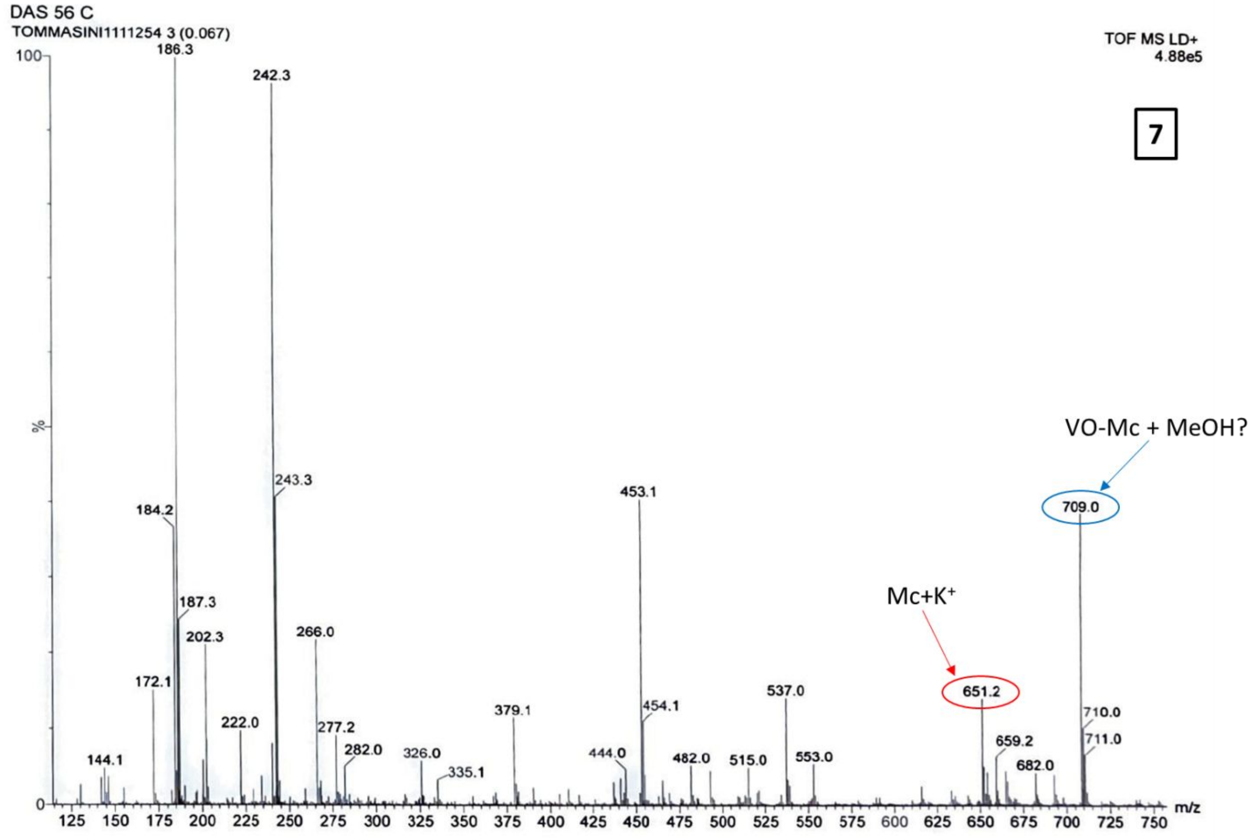

Figures S62. MALDI-TOF Analysis of the Vanadyl Complex of **7**.
